# Health Literacy in Inflammatory Bowel Disease: A Systematic Review of Health Outcomes, Predictors and Barriers

**DOI:** 10.3390/jcm14238577

**Published:** 2025-12-03

**Authors:** Caterina Mercuri, Rita Nocerino, Vincenzo Bosco, Teresa Rea, Vincenza Giordano, Michele Virgolesi, Patrizia Doldo, Silvio Simeone

**Affiliations:** 1 Clinical and Experimental Medicine Department, Magna Graecia University, 88100 Catanzaro, Italy; doldo@unicz.it (P.D.); silvio.simeone@unicz.it (S.S.); 2 Department of Translational Medical Sciences, University of Naples “Federico II”, 80131 Naples, Italy; rita.nocerino@unina.it; 3 Department of Medical and Surgical Sciences, University Hospital Mater Domini, Magna Graecia University, 88100 Catanzaro, Italy; vincenzo.bosco@unicz.it; 4 Department of Public Health, University of Naples “Federico II”, 80131 Naples, Italy; teresa.rea@unina.it (T.R.); vincenza.giordano@unina.it (V.G.); michele.virgolesi@unina.it (M.V.)

**Keywords:** health literacy, inflammatory bowel disease, treatment adherence, self-care, telemedicine, quality of life

## Abstract

**Background/Objectives**: Inflammatory bowel disease (IBD), including Crohn’s Disease and Ulcerative Colitis, requires complex treatment and active patient participation. Health literacy (HL), defined as the ability to access, understand, and apply health information, is a key factor influencing adherence, self-management, and quality of life in chronic illness. However, evidence regarding HL in IBD remains limited and fragmented. This systematic review aimed to synthesize existing literature on HL in IBD, exploring its impact on outcomes, as well as its predictors and barriers. **Methods**: A systematic search of PubMed, Scopus, CINAHL, and Cochrane Library identified studies published between January 2000 and August 2025 in English or Italian. Eligible studies examined HL among adults with IBD and its associations with clinical, behavioral, or psychosocial outcomes. Methodological quality was assessed using the QuADS tool. Due to heterogeneity across studies, a narrative synthesis was conducted. **Results**: Seventy studies were included, comprising observational, qualitative, mixed-methods, and interventional designs. Higher HL was consistently associated with better treatment adherence, self-management, communication with healthcare providers, and quality of life. Conversely, low HL was linked to poor adherence, greater disease activity, and lower psychological well-being. Predictors of low HL included older age, lower education, minority status, and socioeconomic disadvantage. Barriers included inadequate communication, lack of tailored information, cultural and linguistic challenges, and the digital divide. Interventions such as structured education, telemedicine, and digital tools showed potential to improve HL and patient engagement. **Conclusions**: HL is a crucial determinant in IBD management. Enhancing HL through integrated clinical, educational, and digital strategies is essential to improve outcomes and reduce health disparities.

## 1. Introduction

Inflammatory bowel disease (IBD) is a chronic, relapsing–remitting condition characterized by inflammation of the gastrointestinal tract, encompassing two primary subtypes: Crohn’s Disease (CD) and Ulcerative Colitis (UC) [1]. These disorders exert a profound impact on patients’ physical and psychological well-being, quality of life (QoL), and daily functioning [2]. Effective management requires long-term adherence to complex treatment regimens, regular follow-up, lifestyle adjustments, and active engagement in self-care practices [3,4]. Given its chronic nature, IBD imposes a considerable burden not only on healthcare systems but also on patients, who are expected to participate actively in the management of their condition [5,6].

In recent years, particular attention has been paid to digital health literacy (DHL), defined as the ability to access, interpret, and apply digital health information. With the rapid growth of eHealth technologies, DHL has become especially relevant for IBD, where electronic tools are increasingly used for disease monitoring, medication adherence, and patient–provider communication. The COVID-19 pandemic further accelerated the reliance on digital tools for chronic disease management, making DHL an essential competency for equitable access to care [7,8]. Patients with low DHL are less able to benefit from telemedicine, web-based education, and mobile applications, thereby exacerbating disparities in disease management [9,10].

Within this context, health literacy (HL) emerges as a critical determinant of effective disease management [11,12]. HL refers to an individual’s capacity to obtain, understand, evaluate, and apply health information to make informed health-related decisions. It encompasses a spectrum of cognitive and social skills and is recognized as a dynamic, multidimensional construct that extends beyond functional literacy, the ability to read and comprehend medical information, to include communicative and critical literacy, such as engaging in shared decision-making and assessing the credibility of information sources [13,14]. In chronic illnesses like IBD, adequate HL enables patients to navigate the healthcare system, understand treatment options, adhere to prescribed therapies, and monitor symptoms effectively [9]. Despite its recognized importance, HL remains suboptimal in many patients with IBD and is influenced by factors such as sociodemographic characteristics, disease severity, and access to care [9,15,16,17,18,19]. Limited HL has been associated with worse outcomes in chronic diseases, including lower treatment adherence, reduced self-management capabilities, and diminished QoL [20,21].

Furthermore, sociodemographic determinants, including lower educational attainment, limited income, minority ethnicity, and older age, have been consistently linked to limited HL in IBD. These inequities restrict access to reliable health information, reduce participation in shared decision-making, and ultimately contribute to poorer clinical outcomes and increased psychological burden in disadvantaged groups [17,18].

Although the role of HL in chronic disease management is well established, its specific impact on IBD outcomes has not yet been comprehensively synthesized. Existing studies have explored HL in IBD across various domains, including medication adherence [22], digital engagement [10], dietary information needs [23] and patient–provider communication [24], yet the evidence remains fragmented. A more integrated understanding of how HL influences health outcomes in IBD, along with the identification of key predictors and barriers, is lacking. This systematic review aims to fill this gap by synthesizing the current literature on HL in IBD, with particular attention to its impact on patient outcomes and to the factors that facilitate or hinder the development of adequate HL.

## 2. Objectives

### 2.1. Primary Aim

To assess the impact of HL on health outcomes in individuals with IBD, including treatment adherence, self-management behaviors, QoL, and clinical indicators such as disease activity and relapse.

### 2.2. Secondary Aims

To identify key predictors of adequate HL among patients with IBD and to explore the main barriers, structural, communicative, cognitive, or digital, that hinder effective HL in this population.

## 3. Materials and Methods

### 3.1. Study Design

This systematic review was conducted between June and September 2025 in accordance with the PRISMA 2020 (Preferred Reporting Items for Systematic Reviews and Meta-Analyses) guidelines [25]. The review protocol was registered in PROSPERO (registration ID: CRD420251126437) on 14 August 2025.

### 3.2. Search Methods

The research question was formulated using the PEO framework (Population, Exposure, Outcome), as reported in Table S1 (Supplementary Materials). The population included adult patients (aged ≥ 18 years) with IBD. The exposure was the level of HL and its determinants, and the outcomes concerned treatment adherence, self-management, quality of life, and related barriers.

The electronic search was conducted in the following databases: PubMed, Scopus, CINAHL Complete, and Cochrane Library.

The search string, adapted for each database, was the following:

(“health literacy” OR “health knowledge” OR “health understanding” OR “patient education” OR “health communication” OR “self-care” OR “disease management”)

AND (“inflammatory bowel disease” OR “IBD” OR “Crohn’s disease” OR “ulcerative colitis”) AND (“health outcomes” OR “treatment adherence” OR “self-management” OR “quality of life” OR “risk factors” OR “predictors” OR “barriers” OR “communication difficulties” OR “information access” OR “communication barriers”)

The search was limited to articles published in English or Italian between January 2000 and August 2025. We selected a 25-year timeframe (1999–2024) to ensure comparability across studies, as the conceptualization and measurement of health literacy have significantly evolved since the late 1990s. This time restriction was therefore applied to balance comprehensiveness with methodological consistency. No restrictions were applied with respect to the study design.

### 3.3. Inclusion and Exclusion Criteria

To ensure consistency and transparency in the selection process, predefined eligibility criteria were established before screening. These criteria aimed to capture studies directly addressing HL in IBD while excluding those not relevant to the research objectives.

Inclusion criteria:

(a) Articles in English or Italian;
(b) Studies on adult populations (≥18 years);
(c) Peer-reviewed original research articles of any design (observational, qualitative, mixed-methods, randomized controlled trials, systematic reviews);
(d) Publications within the last 25 years;
(e) Studies analyzing the impact of HL in patients with IBD in terms of clinical or behavioral outcomes, predictors, and barriers;
(f) Studies conducted in hospital-based or clinical settings, from all-income countries.

Exclusion criteria:

(a) Pediatric populations;
(b) Non–peer-reviewed articles (editorials, letters, conference abstracts without full text);
(c) Studies not specifically involving IBD patients;
(d) Studies not directly assessing HL or lacking sufficient data for outcome analysis.

Previously published systematic reviews were included to support contextual interpretation of the field and to facilitate backward citation searching, in order to identify additional eligible primary studies and avoid missing relevant data [26,27].

### 3.4. Search Outcome

The search initially identified 2376 records: PubMed (n = 726), CINAHL Complete (n = 180), Cochrane (n = 41), and Scopus (n = 1379). All records were uploaded and managed through Rayyan, Intelligent Systematic Review software to facilitate the screening process and reduce the risk of bias (online version accessed in 2025). After removal of 752 duplicates, 1624 records were screened by title and abstract. This phase was conducted independently and in a blinded manner by two reviewers (CM and RN); any conflicts were resolved through consensus discussion. Of the 1624 records, 1421 were excluded as not relevant. A total of 203 full-text articles were assessed for eligibility, of which 9 could not be retrieved and 124 were excluded for not meeting the inclusion criteria. Ultimately, 70 studies were included in the final review.

The entire process of identification, screening, eligibility, and inclusion is illustrated in the PRISMA 2020 flow diagram (Figure S1) [25].

### 3.5. Quality Appraisal

The methodological quality of the included studies was assessed using the QuADS (Quality Assessment with Diverse Studies) tool, selected for its reliability and validity in analyzing different research designs, including quantitative, qualitative, mixed and multi-method studies. The tool comprises 13 assessment criteria, each with a score between 0 (minimum) and 3 (maximum), for a maximum total score of 39 [28,29]. The assessment was carried out independently by two reviewers, with any discrepancies resolved through discussion and consensus. A final percentage score was calculated for each article by comparing the total score achieved with the theoretical maximum score ([final score = total study score/39 × 100%]).

Overall, the methodological quality of the included studies was heterogeneous. Some RCTs and prospective studies achieved high scores (>90%), indicating good methodological soundness. In contrast, a considerable number of observational studies and surveys scored in the medium range (approximately 69–80%), mainly due to limitations such as small sample sizes, reliance on non-validated tools, and restricted generalizability. The detailed scores for each study are reported in Table S2.

Moreover, a visual summary of the distribution of QuADS percentage scores across the four quality ranges is presented in Figure S2.

### 3.6. Data Synthesis

Given the substantial heterogeneity of study designs, populations, measurement tools, and reported outcomes, a quantitative meta-analysis was not feasible. Instead, a narrative synthesis was performed in line with PRISMA 2020 recommendations, aiming to integrate the available evidence and highlight convergences, divergences, and knowledge gaps [25].

The included studies were grouped into the following main thematic domains concerning health literacy in IBD:

1. Impact on treatment adherence;
2. Influence on self-management behaviors;
3. Effects on quality of life and psychological well-being;
4. Associations with clinical and healthcare outcomes (e.g., disease activity, hospitalizations, therapeutic escalation);
5. Predictors and barriers to HL.

Within each domain, findings were compared descriptively, considering sample characteristics and contextual factors. Whenever possible, evidence from more robust study designs (e.g., RCTs, longitudinal studies) was contrasted with observational and qualitative research to provide a balanced critical interpretation.

To improve interpretability and facilitate comparison across heterogeneous study designs, extracted outcomes were grouped a priori into five thematic domains (treatment adherence; self-management behaviors; quality of life and psychological wellbeing; clinical and healthcare outcomes; predictors and barriers/determinants). The assignment to domains was performed by reading the outcome measures and main findings reported in each study. Differences in classification were resolved by discussion until agreement.

Quantitative values were not pooled because outcome measures differed substantially in scale, units of measurement and reporting format across studies. Heterogeneous effect metrics (e.g., psychometric scores, utilization counts, correlations) were therefore summarized directionally, whereas prevalence estimates were reported only when comparable across ≥2 studies.

## 4. Results

A total of 70 studies were included in the synthesis. Given the heterogeneity of populations, study designs and reported outcomes, the results are presented stratified into five thematic domains (treatment adherence; self-management behaviors; quality of life and psychological wellbeing; clinical and healthcare outcomes; predictors and barriers/determinants). This re-organization was applied consistently to enhance readability and interpretability. The complete stratified data extraction table, organized according to the five thematic domains, is reported in the Supplementary Materials (Table S3A–E).

Although heterogeneity did not allow for pooling effect sizes in a meta-analytic model, several studies reported quantitative estimates. In particular, the prevalence of low health literacy ranged from 17.0% to 47.5% across included samples (n = 70 studies), with older age and African American race being consistently associated with lower HL levels. Moreover, individual studies reporting effect metrics showed that lower HL was associated with poorer QoL scores and worse self-management behaviors, supporting the observed narrative patterns (Table S4).

### 4.1. Overview of Included Studies

A total of 70 studies were included in this systematic review, comprising a heterogeneous body of literature encompassing qualitative studies, cross-sectional observational studies, randomized controlled trials (RCTs), and systematic reviews The studies were conducted across a wide range of geographical contexts including Europe, North America, Asia, and Australia. Sample sizes varied from small qualitative interviews with fewer than 30 participants to large-scale survey-based investigations involving over 2000 patients and healthcare professionals.

Across the included studies, considerable heterogeneity emerged in terms of study populations, measurement tools, and outcome definitions, which limits the comparability of findings and requires cautious interpretation [30].

In addition, most studies relied on generic HL assessment tools rather than disease-specific instruments, which may have limited their capacity to capture the nuances of HL in the IBD context [31].

Several studies evaluated HL in specific contexts such as DHL [9], pregnancy-related issues knowledge in IBD [21], and medication-specific knowledge [20]. Others focused on broader constructs such as the impact of HL on treatment behaviors [32] and psychosocial functioning [6]. Interventions included educational programs [22], telemedicine [33] and group-based psychoeducational sessions [34]. A detailed description of the characteristics of the included studies is reported in Table S3A–E.

### 4.2. Impact of HL on Treatment Adherence

HL emerged as a significant determinant of medication adherence in patients with IBD, although the relationship was complex and sometimes mediated by additional factors such as patient beliefs, age, or psychological status. Although several studies confirmed a positive association between adequate HL and medication adherence, this relationship was not universal. Some cohorts reported no significant correlation, suggesting that psychological distress, perceived treatment burden, or the quality of patient–provider interaction may mediate this link [22,32]. In a systematic review by Knowles et al. (2020) [32] nonadherence rates were found to be high, ranging from 65–90% in adolescents, and 55–70% in adults. Factors influencing adherence included patients’ understanding of their condition, communication quality with healthcare providers, and mental health status. The review emphasized the need for multicomponent interventions, including education and motivational interviewing, particularly among younger patients, to improve HL and thereby adherence. In a cross-sectional study by Wang et al. (2020) [22], a structured educational program significantly improved adherence to azathioprine maintenance therapy in patients with CD. Notably, low medication knowledge and limited belief in treatment necessity were among the most prominent predictors of nonadherence. Following the educational intervention, improvements were observed in medication knowledge and adherence, as well as in clinical outcomes such as reduced relapse rates.

Similarly, Tae et al. (2016) [20] found that limited knowledge about prescribed medications was significantly associated with nonadherence, which in turn correlated with higher relapse risk. Younger age and longer intervals between outpatient visits were also associated with reduced adherence, highlighting the relevance of ongoing patient education.

Conversely, a study by Moradkhani et al. (2011) [35] reported that although increased disease-related knowledge correlated with better coping strategies, it did not translate into significantly improved adherence. This suggests that while HL supports psychological adaptation, it may require complementary interventions to influence actual health behaviors.

Pittet et al. (2014) [36] reported an unexpected finding: IBD patients who actively sought information were more likely to be noncompliant. This was interpreted as a potential indicator of distrust in healthcare providers or dissatisfaction with previously received information, underlining the importance of not only quantity but also the quality and trustworthiness of information sources.

Overall, the evidence indicates that HL plays a pivotal role in promoting adherence, especially when reinforced through structured and interactive educational interventions. However, HL alone may be insufficient in certain contexts, and its effects are most pronounced when integrated within a broader, supportive healthcare relationship (Table S3A).

### 4.3. Impact of HL on Self-Management Behaviors

HL has been shown to positively influence self-management behaviors in individuals with IBD, particularly when accompanied by supportive interventions aimed at increasing disease-related knowledge and self-efficacy. In the structural equation modeling study by Zhu et al. (2025) [6] both self-efficacy and self-management behaviors emerged as significant mediators between disease activity and disease control. Higher levels of HL contributed to better self-management and perceived control of the disease, reinforcing the value of HL-enhancing strategies in fostering autonomous disease management. Quantitative analyses revealed marked disparities between HL clusters: patients with lower HL reported significantly lower self-efficacy and engagement in health-promoting behaviors, whereas higher HL was associated with greater confidence in disease monitoring and lifestyle adjustment [9].

Maurud et al. (2025) [9] identified two clusters of patients based on HL and DHL. Patients with lower HL/DHL demonstrated reduced self-efficacy and poorer health status. These findings suggest that HL not only facilitates access to information but also underpins key behavioral and psychological capacities that are necessary for self-management.

The development and evaluation of educational resources also reflect HL’s centrality to self-management. Norouzkhani et al. (2023) [37] reported that a patient-centered educational book, validated through patient and expert feedback, was highly rated for usability and understandability. While its effect on actual behavior change was not measured, the study underscored that patients with higher HL were better able to navigate the educational material and expressed greater confidence in managing their condition.

In a feasibility study of a Home Telemanagement System (HAT), Cross et al. (2007) [33] observed that patients engaging in self-monitoring activities and receiving structured feedback reported improved disease knowledge and trends toward improved disease activity and QoL. These behaviors were facilitated by regular prompts and symptom tracking—activities that presuppose a baseline level of HL and motivation.

Additionally, the mixed-methods study by Maurud et al. (2024) [38] highlighted that both patients and healthcare professionals prioritize practical, understandable, and accessible information as essential to supporting self-management in daily life. Personalization and clarity, two hallmarks of effective HL interventions, were identified as critical to the success of digital tools.

In summary, HL facilitates the acquisition and application of skills necessary for effective self-management in IBD. When patients understand their condition, trust the information provided, and possess the self-efficacy to act on it, they are more likely to engage in proactive behaviors that promote disease control and reduce the need for escalated care (Table S3B).

### 4.4. Impact of HL on QoL

The relationship between HL and QoL in patients with IBD is supported by multiple studies, suggesting that greater knowledge and comprehension of the disease positively influence psychological well-being, disease acceptance, and daily functioning.

In the cross-sectional study by Maurud et al. (2025) [9] patients with lower HL and digital HL reported significantly poorer health status and QoL, alongside reduced self-efficacy and longer disease duration. This cluster of patients was also more likely to experience challenges in accessing and interpreting health information, which may further compromise QoL by limiting their ability to effectively engage with disease management strategies.

Reusch et al. (2016) [39] assessed the impact of a psychoeducational self-management program on QoL and psychosocial well-being. Although no significant differences were observed between the intervention and control groups, likely due to both groups receiving similar information, the study reported improvements over time in QoL and disease-related concerns, suggesting a general benefit from educational exposure and engagement.

Similarly, Jackson et al. (2016) [5] found that eHealth interventions, including telemonitoring, digital symptom tracking, and video education, were associated with improvements in patient-reported outcomes such as QoL, anxiety, and disease activity. The effectiveness of these interventions was often mediated by patients’ ability to understand and apply the information provided, highlighting HL as a core component of their success.

In the development and evaluation of a digital educational booklet, Norouzkhani et al. (2023) [37] reported high satisfaction and perceived usefulness among patients, suggesting that tailored, easy-to-understand resources may indirectly support QoL by improving patients’ confidence in managing their condition.

Moreover, McMaster et al. (2012) [34] showed that participants in a structured support group reported high satisfaction and perceived emotional support, which contributed to better coping and QoL. The combination of shared experiences and educational components likely enhanced HL, fostering a greater sense of control and well-being.

Overall, the evidence suggests that HL contributes to QoL both directly, by improving patients’ understanding and acceptance of their condition, and indirectly, through enhanced self-efficacy, reduced anxiety, and improved interactions with healthcare providers (Table S3C).

### 4.5. Impact of HL on Health-Related Outcomes

HL has been shown to influence several clinical and health-related outcomes in patients with IBD, including disease activity, need for therapeutic escalation, relapse rates, and healthcare utilization. However, not all studies confirmed a direct effect. For example, some investigations reported no significant association between HL and disease activity, suggesting that contextual factors such as access to care, social support, and comorbidities may attenuate or amplify the role of HL [40]. Consistently, Park et al. (2020) [41] demonstrated that higher disease-related knowledge was associated with a reduced need for therapeutic escalation, further underscoring the link between HL and clinically meaningful outcomes [41]. Park et al. (2020) [41] conducted a prospective cohort study demonstrating that patients with higher scores on the IBD-KNOW questionnaire had a significantly lower risk of requiring step-up therapy (e.g., corticosteroids, immunomodulators, or biologics). This suggests that greater disease-specific HL may reduce disease progression and the need for more aggressive treatments, potentially leading to lower healthcare costs and improved clinical stability.

Tae et al. (2016) [20] also found a significant association between poor medication knowledge and higher relapse risk over an 18-month follow-up period. Patients with limited HL were more likely to misunderstand the importance of consistent therapy, which contributed to disease flare-ups and suboptimal outcomes.

In the randomized study by Wang et al. (2020) [22] patients who participated in an educational program about Azathioprine (AZA) showed not only improved adherence but also a significantly lower rate of endoscopic relapse compared to those who did not. The improvement in health outcomes was mediated by an increase in treatment-specific HL, particularly in understanding AZA’s role in maintenance therapy.

Cross et al. (2007) [33] reported that participants using a telemanagement system demonstrated trends toward improved disease activity and QoL, though statistical significance was not reached. Importantly, these improvements coincided with increased patient knowledge and engagement, suggesting that HL-enhancing interventions may contribute to better disease control, even if indirectly.

Jackson et al. (2016) [5] supported these findings, showing that eHealth tools tailored to patients’ HL levels were associated with improved disease indices, medication adherence, and fewer disease flares. However, the authors emphasized that the reliability of self-reported data and the heterogeneity of the interventions were potential limitations.

In summary, multiple studies support the role of HL in improving health-related outcomes in IBD. By enhancing patients’ understanding of their disease and treatment, HL may reduce relapse risk, delay therapeutic escalation, and promote more stable disease control (Table S3D).

### 4.6. Determinants and Predictors of Health Literacy in IBD

The determinants of HL among patients with IBD can be broadly categorized into sociodemographic, clinical, and contextual factors.

Sociodemographic determinants. Several studies have demonstrated that age, education, and socioeconomic status strongly influence HL. For instance, younger age, higher educational attainment, and higher income were significantly associated with improved disease-specific knowledge in Chinese patients, as shown by Xie et al. (2023) [42] in their validation of an IBD knowledge questionnaire. Similarly, Selinger et al. (2012) [21] observed that women with IBD who were Caucasian, of higher socioeconomic background, had children, or were members of patient associations scored better in pregnancy-related knowledge, underlining the role of social and structural factors in shaping HL. These findings indicate that HL is unequally distributed and reflects broader health disparities.

Clinical determinants. Disease-related variables also affect HL levels. Maurud et al. (2025) [9] identified disease activity and treatment with biologics as strong predictors of lower HL and digital HL, with patients in the low HL cluster also reporting longer disease duration and worse self-efficacy. In contrast, patients with CD were sometimes found to have higher disease knowledge compared to those with ulcerative colitis, possibly reflecting the greater complexity of CD management [42]. Frequency of outpatient visits also emerged as a positive predictor of HL, as highlighted by Tae et al. (2016) [20], since regular follow-up creates more opportunities for education and patient–provider interaction.

Contextual determinants. The healthcare environment and organization of care also play a significant role. Laube et al. (2020) [43] showed that women followed in specialized IBD-pregnancy clinics demonstrated higher HL compared to those managed in general IBD clinics, illustrating the impact of tailored care models. Membership in patient advocacy groups and participation in structured educational programs were also identified as independent predictors of improved HL [22]. Together, these findings highlight that HL in IBD is not static but shaped by a dynamic interplay of personal, clinical, and contextual factors (Table S3E).

Furthermore, a visual synthesis of the main predictors and barriers influencing health literacy and its impact on self-management, knowledge, and health behaviors is presented in Figure S3.

### 4.7. Barriers to Effective HL in IBD

Barriers to achieving effective HL in IBD can be grouped into sociodemographic, clinical-behavioral, and structural or organizational domains.

Sociodemographic barriers. Sociodemographic barriers are consistently reported as key determinants of inadequate health literacy in patients with inflammatory bowel disease. Evidence shows that older age and African American race are strongly associated with low HL, with African Americans presenting significantly higher rates of limited HL compared to white patients (47.5% vs. 17.0%) [17]. Similarly, lower educational attainment and low family income have been identified as independent predictors of poor disease-related knowledge, highlighting the vulnerability of patients from disadvantaged socioeconomic backgrounds [19]. Further, a large Italian cohort demonstrated that inadequate food literacy, linked to sociodemographic characteristics, correlated with poorer health status and greater limitations in daily activities, reinforcing the role of literacy inequalities in shaping patient outcomes [18]. In addition, mapping studies of HL and digital HL in IBD confirmed that clinical factors, disease duration, and demographic conditions interact with health literacy levels, with lower HL/DHL clusters showing worse health status and self-efficacy [38]. Collectively, these findings indicate that disparities tied to education, income, ethnicity, and disease-related characteristics compromise patients’ ability to access reliable information and fully engage in informed decision-making.

Older age, minority ethnicity, lower educational attainment, and reduced income consistently predicted lower HL levels across diverse cohorts. These findings align with qualitative reports describing difficulties in accessing trustworthy information and engaging in shared decision-making. In addition to individual characteristics, system-level barriers such as limited consultation time, insufficient educational resources, and digital exclusion were also frequently reported [16,17,18].

Clinical and behavioral barriers. Psychological distress, including anxiety and depression, was repeatedly associated with lower adherence and reduced HL, as found by Wang et al. (2020) [22] in their study on azathioprine adherence. Moreover, distrust in healthcare providers or reliance on poor-quality information sources may paradoxically worsen HL: Pittet et al. (2014) [36] showed that patients actively seeking information were paradoxically more likely to be non-adherent, possibly due to dissatisfaction with medical advice.

Structural and organizational barriers. Time constraints during consultations, insufficient access to specialized services, and lack of personalized educational materials were commonly reported barriers. For example, Watanabe et al. (2022) [44] showed that in Japan, physicians tended to prioritize treatment-related topics over quality-of-life concerns, leading to a mismatch in expectations. These findings reinforce that structural and communication barriers remain central obstacles to effective HL in IBD (Table S3E).

## 5. Discussion

The management of IBD requires the active involvement of patients, who are called upon to navigate complex treatment regimens, recognize the early signs of flare-ups and implement appropriate self-management behaviors. In this context, HL emerges as a key determinant, not only for the ability to understand health information, but also for its impact on clinical, psychological and social outcomes. This systematic review helps to fill a previously fragmented knowledge gap, demonstrating that an adequate level of HL is associated with better treatment adherence, more robust self-management and a better quality of life in patients with IBD. These findings can be better understood when framed within established HL models. The tripartite model proposed by Nutbeam et al. (2021) [12] identifies three progressive levels of health literacy: functional, communicative, and critical. These dimensions represent gradual development of skills needed to understand and use health information, participate in shared decision-making, and ultimately influence the determinants of health [12]. Similarly, Sørensen’s et al. (2015) [14] integrated model conceptualizes HL as encompassing access, understanding, appraisal, and application of health information across healthcare, disease prevention, and health promotion domains. Situating IBD-related evidence within these frameworks emphasizes the multidimensional and context-dependent nature of HL. A recurring theme highlighted across the studies is the strong link between health literacy and treatment adherence. Several studies have shown that higher levels of HL are associated with better adherence to drug treatments, reducing the risk of suspensions, forgetfulness or treatment abandonment. For example, Selinger et al. (2012) [21] reported that patients with higher scores on the CCPKnow questionnaire, indicative of greater knowledge of the disease, showed better treatment adherence and more effective clinical control. Similarly, Wang et al. (2020) [22] highlighted that knowledge of the disease is a significant predictor of correct medication intake and reduced complications. This relationship underscores the importance of patient education and the dissemination of accurate health information, which are vital for promoting engagement and improving health behaviors among patients with IBD [9,45].

In fact, structured educational interventions have been shown to be effective in improving adherence. Fiorindi et al. (2022) [18] showed that targeted education programs increase patient awareness, with a positive impact on medication adherence. Similarly, Chowdhary et al. (2021) [46] highlighted that short training activities focused on specific aspects of disease management help to strengthen therapeutic consistency.

Research suggests that educational interventions tailored to individual patient needs tend to be more effective, highlighting the need for personalized approaches to patient education [37,47,48].

Integrating patient education with modern technology, such as social media platforms, can improve the accessibility and appeal of educational materials, thereby potentially improving adherence rates [46,49].

A recent RCT showed that the use of social media platforms, such as Instagram, can be an effective channel for health education: frequent interaction with dedicated content led to a significant increase in patient knowledge and activation [50]. At the same time, telemedicine has established itself as a valuable tool for delivering educational interventions and promoting active engagement, facilitating more effective self-management through real-time communication between patients and healthcare professionals [51]. Several digital solutions have shown promising results in improving treatment adherence and patient engagement. Among these, web-based platforms such as Constant-Care [52] and telemonitoring systems such as TELE-IBD [52] and HAT [53] have shown benefits in terms of adherence and disease knowledge. Similarly, mobile applications such as HealthPROMISE [54] have been shown to strengthen follow-up and engagement, while more recent protocols, the ASSIST study [55] evaluated the Tappt web application, which allows users to set personalized medication reminders and access dedicated educational resources, demonstrating its potential to improve patient engagement and treatment adherence. However, the effectiveness of digital health literacy interventions is strongly context-dependent. In low-resource settings, socioeconomic constraints, limited internet availability, lack of device access, and reduced digital infrastructure may substantially restrict the scalability, uptake and real-world impact of such tools. Moreover, language barriers, affordability issues and differing cultural meanings of health information may undermine the transferability of results from high-income countries. These contextual limitations highlight that digital HL-based interventions are not automatically equitable and that specific implementation strategies are required to ensure effectiveness across diverse settings.

From an implementation perspective, the practical adoption of HL-oriented interventions varies considerably across healthcare settings. In high-resource contexts, digital tools such as telemonitoring platforms and app-based reminders can be integrated into existing care pathways with relative ease, showing promising effects in improving treatment adherence, patient activation and symptom monitoring. However, scalability and equity remain contingent on contextual factors such as stable internet access, affordability, availability of multilingual interfaces and flexibility for patients with limited digital skills. In low-resource settings, adoption may require hybrid approaches combining digital components with simplified print-based materials, caregiver involvement or community-mediated education. These considerations highlight that the effectiveness of digital interventions must be interpreted alongside their accessibility profiles, and that implementation strategies need to be carefully adapted to the structural and sociocultural characteristics of different healthcare systems.

Literature has shown that low levels of health literacy are associated with lower treatment adherence. Moradkhani et al. (2011) [35] and Matthias et al. (2021) [56] reported that poor understanding of the implications of therapies and insufficient clear information on side effects can result in treatment abandonment or irregularity. Similarly, Hajlaoui et al. (2023) [57] observed that patients with limited HL have more frequently active disease and lower levels of self-efficacy, factors that contribute to further reducing adherence. In line with these findings, other studies have documented how low HL in patients with IBD is associated with unfavorable clinical outcomes, including higher rates of hospitalization and lower adherence to treatment regimens [17,58]

In addition to treatment adherence, HL plays a key role in the development of appropriate self-care behaviors in patients with IBD. High HL enables patients to recognize symptoms, monitor the progression of their condition, adopt healthy lifestyle choices and communicate more effectively with healthcare professionals. Several observational studies have documented this relationship. Rubin et al. (2021) [59] reported that patients with higher HL demonstrate a greater ability to manage symptoms and seek early healthcare in the event of a flare-up. Lesnovska et al. (2014) [60], through a qualitative study, highlighted how better HL promotes not only the management of drug therapy, but also psychological well-being and the adoption of effective coping strategies. Qualitative evidence also offers valuable insights into patients’ lived experiences and the subjective meaning of HL in everyday disease management. Across qualitative studies, patients frequently reported feelings of uncertainty, emotional burden associated with fluctuating symptoms, and difficulties in translating generic information into personalized actions. These narratives reveal that HL is not only a cognitive skill, but also a process rooted in emotional regulation, trust in healthcare providers, and the ability to negotiate ambiguity in chronic illness. Such experiential dimensions complement quantitative findings and highlight that enhancing HL requires interventions that address not only knowledge, but also communication, reassurance and shared decision-making dynamics.

Self-care management in patients with IBD also includes monitoring changes in physical and emotional status in order to take the most appropriate action. According to the IBD-SELF protocol [61], self-care is divided into three main dimensions: maintenance, monitoring and management. In this context, stress management is a crucial aspect, as high levels of stress are frequently associated with increased disease activity.

Specific tools for assessing knowledge, such as IBD-KNOW [41] and CCPKnow [21] have also demonstrated that higher scores are associated with greater adoption of self-care practices, such as attention to diet, symptom monitoring and prevention of complications. In particular, CCPKnow, developed to assess knowledge about the disease and pregnancy, has shown that a higher level of information correlates with more appropriate management behaviors [21].

The relationship between health literacy and self-management in patients with IBD is particularly evident in the context of nutritional management. Food literacy, understood as the ability to find, understand and apply food information, is in fact a crucial factor in adapting dietary choices to the needs of the disease [62]. Limited knowledge can prevent patients from recognizing potentially aggravating foods or selecting those with protective effects, while adequate nutrition education promotes self-care and contributes to improved health outcomes. About that, the scoping review by Norouzkhani et al. (2023) [37] showed that patients with IBD have complex information needs, especially regarding diet, medication and side effects, and that a lack of adequate information is a significant barrier to effective disease management. Furthermore, patients with lower levels of HL report greater difficulty in recognizing early signs of flare-ups, adhering to dietary recommendations, and developing effective coping strategies; these limitations are associated with an increased risk of complications and a poorer quality of life [21,56].

In this context, HL is a key resource not only for clinical aspects but also for psychosocial aspects [63,64]. Interventional studies confirm this role: Berding et al. (2016) [65] observed that a targeted educational program improves knowledge about the disease and promotes more effective coping strategies. Interventions aimed at strengthening these skills, through health education, personalized counselling and digital tools, therefore represent a concrete opportunity to increase patient empowerment and optimize clinical outcomes [66,67].

The relationship between health literacy and QoL emerged consistently from the included literature. Adequate HL allows patients with IBD not only to better understand their clinical condition, but also to reduce anxiety related to the uncertainty of the disease, improving psychological well-being and social participation.

Observational and qualitative studies have documented that higher HL is associated with higher levels of QoL. Lesnovska et al. (2014) [60], through interviews with IBD patients, highlighted how greater knowledge of the disease promotes a sense of control and reduces social stigma, with positive impacts on daily life. Knowles et al. (2020) [32] confirmed that patients with higher HL report better QoL both physically and mentally, emphasizing the role of understanding treatments and the ability to communicate effectively with healthcare professionals.

Ali Ibrahim (2024) [68] highlighted that the implementation of an educational program in patients with IBD leads to a significant improvement in both quality of life and health-promoting behaviors, confirming the value of educational interventions in strengthening empowerment and self-management.

Knowledge assessment tools, such as IBD-KNOW [41], have also established direct associations between higher scores and more favorable QoL outcomes. Furthermore, Fiorindi et al. (2022) [18] demonstrated that targeted educational programs not only increase knowledge but also have beneficial effects on the perception of overall well-being, reducing stress and uncertainty related to disease management.

Conversely, insufficient levels of HL have been linked to a poorer QoL. Patients with lower literacy skills report feelings of helplessness, difficulty understanding the progression of their disease, and greater psychological vulnerability [21,56]. Studies have shown that patients with poor health literacy often encounter difficulties in understanding their disease, medication management and treatment protocols, thus compromising their health-related quality of life [17,18,38,69,70]. Psychological factors, such as fear of disease progression, may also mediate the relationship between HL and quality of life, amplifying the subjective burden of the condition [71]. Finally, the management of IBD often requires complex clinical decisions, which are particularly difficult in the presence of inadequate HL, with negative repercussions on clinical outcomes and overall well-being [11,72].

Overall, this evidence confirms that HL is a crucial determinant of quality of life in patients with IBD. Improving HL levels not only promotes treatment adherence and self-management, but also reduces the psychological burden of the disease and promotes an approach that is truly centered on the patient’s overall well-being.

Analysis in the literature clearly highlights the factors that promote the development of adequate HL. Among the most relevant are level of education and prior knowledge, which enable patients to deal with therapeutic complexity with greater awareness [41]. Young age and familiarity with digital tools are also an advantage, as they facilitate access to and use of available information resources [39]. Structured educational interventions, such as training and counselling programs, play a decisive role, as they have been shown to improve knowledge and self-management behaviors [18,46]. At the same time, digital technologies—including telemedicine and mobile applications such as TELE-IBD [52] and HealthPROMISE [54] have proven to be promising tools for supporting monitoring and communication, enhancing the therapeutic relationship. The latter, when characterized by clear communication and empathy, is in turn an additional facilitating factor [59,60]. Finally, family and caregiver support helps to reinforce understanding of information and support treatment adherence [61].

Conversely, numerous factors hinder the development of adequate HL in patients with IBD. A review estimated that the overall prevalence of poor health literacy may reach 24% among patients with IBD [17]. Key negative determinants include advanced age and low socioeconomic status, both of which are associated with greater difficulty in understanding and using information, resulting in reduced self-management skills [21,56]. In particular, it has been observed that each additional year of age increases the likelihood of low HL by 15% [73]. Disparities are particularly marked among older adults and minority groups, where cultural factors, language barriers and different beliefs about health contribute to lower levels of HL [17,74]. With regard to gender, no substantial differences have emerged, although some studies have found a higher prevalence of inadequate HL among male patients [73].

Additionally, to sociodemographic factors, various relational and contextual elements also hinder the development of adequate HL. Inadequate doctor-patient communication, characterized by overly technical language and limited time, limits understanding and active participation in care decisions [21,35]. Added to this is the scarcity or inconsistency of available information on crucial aspects such as diet, fertility and pregnancy, which fuels uncertainty and concern in patients [21,56]. The picture is further complicated by the digital divide, which penalizes those who do not have adequate access or technological skills [75,76,77] and by linguistic and cultural barriers, which reduce the accessibility of educational materials for patients from migrant backgrounds or belonging to minorities [78]. Finally, the psychological burden of the disease, marked by anxiety, depression and stigma, can further compromise the ability to process and use information, hindering effective self-management [60].

From a clinical perspective, our findings underscore the importance of systematically assessing HL in IBD care. Tailored educational programs, adapted to patients’ HL levels, could improve medication adherence and disease-related knowledge. Moreover, integrating telehealth solutions and digital tools may bridge gaps for patients with adequate digital HL while targeted support should be designed for those with limited DHL. Dietary counseling and structured self-management interventions, aligned with patients’ literacy capacities, represent additional strategies to enhance engagement and outcomes [9].

However, the effectiveness of educational or digital interventions may be reduced when moving from theory to their application in clinical practice. In this context, difficulties arise due to unequal access to resources, variability in healthcare professionals’ training, and the absence of structured follow-up pathways. These factors contribute to limiting their real impact. In addition, the effectiveness of such approaches may be conditioned by the variability of patients’ socioeconomic and psychological conditions, which play a decisive role in determining their ability to truly benefit from educational or digital interventions.

Overall, these findings are consistent with those observed in other chronic conditions, such as diabetes and heart disease, where higher levels of HL are associated with better clinical and behavioral outcomes [12,14]. However, IBD presents unique challenges: the complexity of treatment regimens, the unpredictability of symptoms and the high psychological burden make HL an even more crucial determinant in ensuring a truly effective therapeutic approach. Therefore, this evidence confirms that HL is not only a cross-cutting factor but also a strategic objective in IBD care pathways. Improving it means intervening on multiple levels, like clinical, educational and digital and should be a priority for both clinical practice and future research.

Future research should focus on designing and testing HL-sensitive interventions in IBD, ideally through randomized controlled trials with sufficient power to assess patient-reported and clinical outcomes. Longitudinal studies are also needed to clarify the causal pathways linking HL with disease course, adherence, and quality of life. Furthermore, there is an urgent need for the development and validation of HL measurement tools specific to IBD, as current studies rely on generic instruments that may not fully capture disease-related literacy needs [40].

This review also revealed important methodological limitations across the included studies. Most investigations were cross-sectional with relatively small sample sizes, limiting causal inferences. A wide heterogeneity was observed in HL measurement, with frequent use of non-validated tools and inconsistent definitions, complicating the comparison of results. Cultural variability was rarely considered, even though HL is highly context-dependent. Addressing these limitations in future research will be essential to strengthen the evidence base. Furthermore, broader conceptual frameworks outside the included studies have also highlighted the intrinsic limitations of applying generic HL instruments to disease-specific contexts such as IBD [79,80]. These works emphasize that standard HL tools often fail to capture condition-specific informational needs, and may therefore underestimate the real impact of literacy gaps in chronic immune-mediated diseases. This conceptual limitation reinforces the need for disease-tailored HL measures and supports the interpretation that heterogeneity in the current literature partly reflects measurement constraints, rather than the absence of true associations.

### 5.1. Strengths and Limitations

This review helps to fill a gap in the literature by providing a systematic synthesis of the available evidence on health literacy (HL) in patients with chronic inflammatory bowel disease. To date, it is one of the few reviews that specifically addresses this topic, providing a comprehensive and multidimensional view of an area that has so far been explored only in a fragmentary manner.

However, certain limitations must be considered. Firstly, the methodological heterogeneity of the included studies, characterized by different designs, prevented a quantitative meta-analysis from being performed. The overall methodological quality, assessed using QUADS, was variable: although some RCTs and prospective studies reported high scores, a significant proportion of studies were in the medium-low range, with sampling limitations, instruments that were not always validated, and reduced generalizability.

In addition, several types of methodological bias were recurrent across the included studies, further influencing the reliability of the synthesized evidence. Selection bias emerged as one of the major sources of distortion, often related to convenience sampling, preferential inclusion of studies with favorable results, or incomplete reporting of recruitment criteria. Unmeasured confounding was a significant limitation in observational studies, exacerbated by inadequate adjustment strategies such as the limited use of propensity score methods. Moreover, the lack or inadequacy of blinding procedures increased the risk of performance and detection bias, potentially leading to subjective influences in outcome assessment. Selective outcome reporting was also frequent, as many studies lacked prospective protocol registration, hindering the identification of unreported or secondary outcomes. Finally, the methodological heterogeneity of study designs, operational definitions, and measurement tools contributed to indirectness and limited comparability between studies. Collectively, these sources of bias resulted in a generally low to very low certainty of evidence, highlighting the need for more robust methodological standards and greater transparency in future research.

From a geographical point of view, most studies come from Europe and North America, while contexts such as Asia, Latin America and Africa are less represented. This limits the transferability of the results to settings with limited resources or less developed health systems.

These imbalances have important implications for global health equity. Regions with limited data availability often have lower digital infrastructure, reduced access to specialist gastroenterology care and greater socioeconomic barriers, which may amplify the impact of limited HL on diagnostic delays, treatment discontinuity and health outcomes. Therefore, the lack of representation from Asia, Latin America and Africa may lead to an underestimation of the true burden and consequences of limited HL in global IBD care. Expanding research in these settings is crucial to ensure more equitable evidence generation and to support the development of context-appropriate educational and digital interventions.

Furthermore, the samples studied often consist of young or well-educated patients, with an underrepresentation of older people, migrants and vulnerable populations, in whom linguistic, cultural and socio-economic barriers can significantly affect HL. Addressing HL disparities in these underrepresented groups requires tailored strategies that go beyond standard educational approaches. Simplified and culturally adapted resources, multilingual content, the use of visual formats and pictograms, as well as the active involvement of caregivers and community mediators, may facilitate comprehension and engagement. In addition, interventions should consider barriers such as limited digital access or lower familiarity with technology, providing support for digital navigation and alternative non-digital options when necessary. These approaches are essential to promote equity and ensure that health literacy interventions are inclusive and effective across diverse patient populations.

A possible linguistic bias should also be noted, as the research was limited to publications in English and Italian, with the risk of excluding relevant studies in other languages. Another limitation lies in the underrepresentation of qualitative evidence synthesis, which could provide deeper insights into patients’ lived experiences of HL challenges. Finally, the scarcity of RCTs specifically dedicated to HL interventions in IBD represents an important gap that reduces the strength of the conclusions.

### 5.2. Implications and Recommendations for Further Research

The clinical implications of this review are significant. The routine use of validated tools, such as IBD-KNOW and CCPKnow, could facilitate the early identification of patients with low HL, allowing for timely and targeted interventions.

Integrating these tools into daily clinical practice, for example during outpatient visits or follow-up assessments, would allow for tailored communication and the calibration of educational interventions, ensuring a truly patient-centered approach.

Structured educational programs, personalized counselling, and digital solutions, including telemedicine, mobile applications, and interactive reminders, are promising strategies for strengthening treatment adherence, supporting self-management, and improving quality of life.

From a clinical perspective, embedding digital programs within hospital information systems or patient portals could enable continuous monitoring, reducing the risk of unrecognized relapses and improving therapeutic appropriateness. Moreover, such tools could facilitate two-way communication, fostering shared decision-making and greater patient empowerment.

Particular attention should be paid to the most vulnerable groups, such as elderly patients, individuals with low socioeconomic status, and linguistic and cultural minorities, who are at higher risk of limited HL and therefore require interventions tailored to their specific needs.

In these cases, adapting educational content with multilingual resources, visual aids, and simplified materials, as well as actively involving caregivers, is a clinically crucial step to reduce inequalities and ensure equitable access to care.

From the research perspective, some clear priorities emerge. It is essential to develop standardized and shared measurement tools that allow for the consistent assessment of HL in patients with IBD and the comparison of results across different contexts. At the same time, high-quality methodological studies, particularly randomized controlled trials and longitudinal studies, are needed to clarify the causal links between HL and clinical outcomes. Another area of interest is digital health literacy, which deserves further study, while ensuring equitable access to digital health technologies. Finally, future research should include caregivers and family members, evaluating shared self-care models that can provide broader support for the daily management of the disease.

## 6. Conclusions

Health literacy emerges as a crucial determinant in the management of Inflammatory Bowel Disease, directly influencing therapeutic adherence, self-management and quality of life. Improving health literacy levels not only empowers patients, but also reduces the clinical, psychological and social burden of the disease. Educational interventions, accessible digital solutions and clear and empathetic healthcare communication are key strategies for integrating HL into clinical practice. In this perspective, the development of shared assessment tools and the implementation of high-quality studies are fundamental steps in consolidating evidence and guiding clinical and healthcare policy decisions. Systematically integrating HL into multidisciplinary IBD care pathways is therefore a priority for truly patient-centered care.

## Data Availability

Data are contained within the article.

## Supplementary Materials

The following supporting information can be downloaded at: https://www.mdpi.com/article/10.3390/jcm14238577/s1, Table S1. Research question based on the PEO framework. Table S2. QuADS Tool (Quality Assessment with Diverse Studies). Table S3A. Treatment Adherence. Table S3B. Self-Management Behaviors. Table S3C. Quality of Life and Psychological Well-Being. Table S3D. Clinical and Healthcare Outcomes. Table S3E. Predictors, Barriers, and Determinants. Table S4. Summary of Key Findings by Outcome Domain. Figure S1. PRISMA 2020 flow diagram for systematic reviews. Figure S2. Distribution of QuADS percentage scores. Figure S3. Predictors and Barriers of Health Literacy in IBD. Supplementary File S1. PRISMA 2020 Abstract Checklist. Supplementary File S2. PRISMA 2020 Checklist. References [81,82,83,84,85,86,87,88,89,90,91,92,93,94,95,96,97,98,99,100,101,102,103,104,105,106,107,108,109,110] are cite in the Supplementary Materials.

## Abbreviations

AZA	Azathioprine
CD	Crohn’s Disease
DHL	Digital Health Literacy
HAT	Home Telemanagement System
HL	Health Literacy
IBD	Inflammatory Bowel Disease
PRISMA 2020	Preferred Reporting Items for Systematic Reviews and Meta-Analyses
QoL	Quality of Life
QuADS	Quality Assessment with Diverse Studies
UC	Ulcerative Colitis
