# Supplementary material for: Health Literacy in Inflammatory Bowel Disease: A Systematic Review of Health Outcomes, Predictors and Barriers
Source: J Clin Med. 2025 Dec 3;14(23):8577. doi: 10.3390/jcm14238577 (PMC12693092; doi:10.3390/jcm14238577)
Supplement: Supplementary file 1 [file jcm-14-08577-s001.zip › Table S1. Research question based on the PEO framework..pdf]

Table S1. Research question based on the PEO framework.

P (Population)	E (Exposure)	O (Outcome)
Adult patients (≥18 years) with inflammatory bowel disease (IBD: Crohn’s disease and ulcerative colitis)	Level of Health Literacy (HL) and its determinants/predictors	Clinical and behavioral outcomes: treatment adherence, self-management, quality of life; predictors and barriers
