# Supplementary material for: Health Literacy in Inflammatory Bowel Disease: A Systematic Review of Health Outcomes, Predictors and Barriers
Source: J Clin Med. 2025 Dec 3;14(23):8577. doi: 10.3390/jcm14238577 (PMC12693092; doi:10.3390/jcm14238577)
Supplement: Supplementary file 1 [file jcm-14-08577-s001.zip › Table S4. Summary of key findings by outcome domain..pdf]

Table S4. Summary of key findings by outcome domain.

**Outcome domain**	**Main direction of evidence**	**Nature of data reported**	**Representative quantitative range (where available)**
Treatment adherence	Lower HL consistently associated with poorer adherence and suboptimal medication-taking behaviours	heterogeneous metrics (mainly directionality; no comparable units of measurement)	–
Self-management behaviours	Lower HL associated with reduced disease-related knowledge, lower self-efficacy and poorer coping strategies	heterogeneous metrics (directional findings)	–
Quality of life & psychological wellbeing	Lower HL associated with worse QoL scores, higher anxiety/depression, poorer psychosocial adjustment	heterogeneous psychometric scales (not directly comparable)	–
Clinical & healthcare outcomes	Lower HL associated with worse clinical status, more flares/relapses and higher healthcare utilisation	mixed metrics (utilisation / activity scores) not comparable	–
Predictors & barriers / determinants	Age, race, socio-economic inequalities frequently identified as predictors of low HL	prevalence measures comparable across studies	**17.0%–47.5%**

HL = health literacy. Directional quantitative trends were retained to reflect heterogeneity of outcome measures; prevalence is reported where comparable across studies.
