# Supplementary material for: Health Literacy in Inflammatory Bowel Disease: A Systematic Review of Health Outcomes, Predictors and Barriers
Source: J Clin Med. 2025 Dec 3;14(23):8577. doi: 10.3390/jcm14238577 (PMC12693092; doi:10.3390/jcm14238577)
Supplement: Supplementary file 1 [file jcm-14-08577-s001.zip › Tables S2 and S3A-E.pdf]

Table S2 QuADS Tool (Quality Assessment with Diverse Studies)

Study (Author, Year)	Theoretical underpinning	Research aims	Research setting	Sample	Data collection	Rationale for choice of data collection tool(s)	Data analysis	User involvement	Ethical considerations	Results - findings	Strengths & limitations	Evidence of value of research	Transferability / generalisability	Total (max 39)	% Score
Jackson [5]	3	3	3	3	3	3	3	3	2	3	3	2	3	38	97%
Mcdermott [30]	3	2	2	2	2	3	2	2	2	2	3	2	2	29	97%
Rubin [31]	3	3	3	2	3	3	3	3	2	3	3	3	3	37	95%
Hueppe [32]	3	3	3	2	3	3	2	3	3	3	3	3	3	37	95%
Cross [33]	3	3	3	2	3	3	2	3	3	2	3	3	3	36	92%
Kennedy [34]	3	3	3	2	3	3	3	3	2	3	2	3	3	36	92%
Elkjaer [35]	3	3	3	2	3	3	2	3	3	2	3	3	3	36	92%
Abutaleb [36]	3	3	3	2	3	3	2	3	2	3	2	3	3	35	90%
Xin Hu [37]	3	3	3	2	3	3	2	3	2	3	2	3	3	35	90%
Zhu (2025) [6]	3	3	3	3	2	2	3	3	2	3	3	2	3	35	90%
Lee et al. [38]	3	3	3	2	2	2	3	3	3	3	2	3	3	35	90%
Pittet [39]	3	3	3	2	2	3	2	3	3	3	3	3	3	35	90%
Gravilescu [40]	3	3	3	2	3	3	3	2	3	3	2	2	3	35	88%
Eaden [41]	3	3	3	2	2	3	2	3	3	2	2	3	3	34	87%
Wang [22]	3	3	2	3	2	2	3	2	3	2	3	2	3	33	85%
Miglioretto [23]	3	3	2	3	3	2	3	2	3	2	2	1	3	33	85%
Park [42]	3	3	3	2	2	2	3	2	2	3	3	2	3	33	85%
Simian [43]	3	3	2	2	3	3	2	2	2	2	3	3	3	33	84%
Atreja [44]	3	3	3	2	2	2	2	3	2	2	2	3	3	32	82%
Norton [45]	3	3	3	2	2	2	2	3	2	2	2	3	3	32	82%
Maurud [9]	3	3	3	2	2	2	2	3	2	2	2	3	3	32	82%
Watanabe [46]	3	3	3	2	2	2	2	2	2	3	3	2	3	32	82%
Fortinsky [47]	3	3	2	2	3	2	2	2	2	3	2	2	3	32	82%
Selinger [21]	3	2	2	2	2	2	2	3	3	2	2	3	3	31	79%
McMaster [48]	3	2	3	2	2	2	3	2	2	3	2	2	3	31	79%
Reich [10]	3	3	3	2	2	2	2	2	2	3	2	2	3	31	79%
Napolitano [49]	3	2	2	2	2	2	2	3	3	2	2	3	3	31	79%
Tormey 2019 [50]	3	3	3	2	2	2	2	2	2	3	2	2	3	31	79%
Deshpande [51]	3	3	2	2	2	3	3	2	2	2	2	2	2	31	79%
Ryan [52]	3	3	2	2	2	2	2	2	2	3	2	2	3	30	77%
Xie [53]	3	2	2	2	2	2	2	2	2	2	3	3	3	30	77%
Gold [54]	3	3	3	2	2	2	2	2	2	3	2	2	2	30	77%
Danion [55]	3	3	3	2	2	2	2	2	2	3	2	2	2	30	77%
Cross [56]	3	3	3	2	2	2	2	2	2	3	2	2	2	30	77%
Con, De Cruz [57]	3	3	3	2	2	2	2	2	2	3	2	2	2	30	77%
Knowles [58]	3	3	3	2	2	2	2	2	2	3	2	2	2	30	77%
Dos Santos Marques [17]	3	3	3	2	2	2	2	2	2	3	2	2	2	30	77%

Cross [59]	3	3	3	2	2	2	2	2	2	3	2	2	2	30	77%
Long [60]	3	2	2	2	2	3	2	2	2	2	3	2	3	29	74%
Gordon [61]	3	2	2	2	2	3	2	2	2	2	3	2	3	29	74%
Tormey 2016 [62]	3	3	2	2	2	2	2	2	2	3	2	2	2	29	74%
Hou [63]	3	2	2	2	2	3	2	2	2	2	2	2	2	29	74%
Ajibululu [64]	3	3	2	2	2	2	2	2	2	3	2	2	2	29	74%
Asadi [65]	3	3	2	2	2	2	2	2	2	3	2	2	2	29	74%
Borgaonkar [66]	3	3	2	2	2	2	2	2	2	3	2	2	2	29	74%
Carels [67]	3	2	2	2	2	2	2	2	2	3	2	2	29	30	74%
Al-Ani [68]	3	2	2	2	2	2	2	2	2	2	2	2	3	28	72%
Laube [69]	3	2	3	2	2	2	2	2	2	2	2	2	2	28	72%
Lesnovska [70]	3	2	2	2	2	2	2	2	2	3	2	2	2	28	72%
Wood, Dallas [71]	3	2	2	2	2	3	2	2	2	2	2	2	2	27	72%
Bernstein [72]	3	2	2	2	2	3	2	2	2	2	2	2	2	28	72%
Khan [73]	3	2	2	2	2	2	2	2	2	2	2	2	3	28	72%
He [74]	3	2	2	2	2	2	2	2	2	2	2	2	3	28	72%
Derbey [75]	3	3	2	2	2	2	2	2	2	2	2	2	2	28	72%
Daher [76]	3	3	2	2	2	2	2	2	2	2	2	2	2	28	72%
Conley [77]	3	3	2	2	2	2	2	2	2	2	2	2	2	28	72%
Maurud [78]	3	3	2	2	2	2	2	2	2	2	2	2	2	28	72%
Abkarian [79]	3	3	2	2	2	2	2	2	2	2	2	2	2	28	72%
Nielsen [80]	3	2	2	2	2	2	2	2	2	2	2	2	2	27	69%
Chowdhary [81]	3	2	2	2	2	2	2	2	2	2	2	2	2	27	69%
Tae [20]	3	2	2	2	2	2	2	2	2	2	2	2	2	27	69%
Reusch [82]	3	2	2	2	2	2	2	2	2	2	2	2	2	27	69%
Norouzkhani [83]	3	2	2	2	2	2	2	2	2	2	2	2	2	27	69%
Fiorindi [18]	3	2	2	2	2	2	2	2	2	2	2	2	2	27	69%
Matthias [84]	3	2	2	2	2	2	2	2	2	2	2	2	2	27	69%
Moradkhani [85]	3	2	2	2	2	2	2	2	2	2	2	2	2	27	69%
Patil [86]	3	2	2	2	2	2	2	2	2	2	2	2	2	27	69%
Reich [87]	3	2	2	2	2	2	2	2	2	2	2	2	2	27	69%
Leong [88]	3	2	2	2	2	2	2	2	2	2	2	2	2	27	69%
Li Guanwei [19]	3	2	2	2	2	2	2	2	2	2	2	2	2	27	69%

Table S3A Treatment Adherence

Title	Author	Study objectives	Study design	Methodology	Intervention	Main findings	Outcome measured	Research question	Limitations	Domain
What do people with inflammatory bowel disease want to know about diet? The dietary information needs of people with inflammatory bowel disease and perceptions of healthcare providers	Chiara Miglioretto [23]	- Explore the perceived dietary information needs of individuals with IBD. - Describe the diet-related perceptions of HCPs providing care to this cohort.	Qualitative study; Non-controlled; Observational; Semi-structured interviews; Thematic analysis; Prospective data collection; Purposive sampling	- Semi-structured interviews with 13 HCPs and 29 people with IBD. - Interviews conducted online via Zoom or face-to-face. - Framework method for thematic analysis. - Adherence to Consolidated Criteria for Reporting Qualitative Research. - De-identified data stored securely. - Iterative thematic analysis process.	Not mentioned (the study is qualitative and does not involve any specific interventions)	- A lack of dietary guidance at diagnosis negatively impacts the patient's journey with food and eating. - The study supports a paradigm shift towards holistic person-centred care for consistent access to dietetics services. - The need for dietary information is accentuated by the lack of guidance at diagnosis, and doctors are perceived as gatekeepers	Perceived dietary information needs of individuals with IBD, perceptions of HCPs, and enablers and barriers to communication	What are the perceived dietary information needs of individuals with Inflammatory Bowel Disease (IBD), and how do healthcare providers perceive these needs?	- Small number of gastroenterologists participated - Self-reported remission by participants with IBD may introduce recall bias - Small sample size affects generalizability - Potential for respondent bias due to participants' strong interest in diet	1. Treatment adherence

						to accessing dietetics care.				
Medication Adherence Across the Life Span in Inflammatory Bowel Disease	Knowles, Simon R [58]	- Review the differences and similarities between adolescent and adult cohorts in relation to nonadherence rates, predictors, and consequences. - Consider modifiable factors and interventions to improve adherence. - Provide an IBD-specific evidence-based review of the literature on treatment adherence.	Systematic review	The methodology involves a comprehensive review of existing literature on IBD adherence, focusing on differences and similarities between adolescent and adult cohorts. It synthesizes findings from various studies to identify rates, predictors, and consequences of nonadherence, and discusses practical adherence-promoting strategies. The paper builds upon systematic reviews like Lenti and Selinger (2017) and highlights the role of nurses in	- Educational approaches for enhancing adherence in adults and adolescents. - Involvement of nurses in adherence promotion strategies, including informal recommendations and complex multicomponent interventions. - Problem-solving skills training for adolescents. - Motivational interviewing strategies as part of multicomponent interventions. - Open and trusting patient-doctor communication. - Paying attention to general mental health factors. - The "SIMPLE"	- Adolescents with IBD tend to have higher adherence rates compared to adults, with a significant difference observed in early adolescence. - Nonadherence is a significant issue in both adolescents and adults with IBD, with rates estimated at 65%-90% for adolescents and 55%-70% for adults. - Adherence is crucial for managing IBD, as nonadherence is linked to increased disease	Medication adherence rates; Consequences of nonadherence (impaired health outcomes, increased disease activity, risk of relapse)	What are the differences and similarities in medication adherence rates, predictors, and consequences between adolescent and adult cohorts with inflammatory bowel disease (IBD)?	- Heterogeneity of available data - Variability in measuring adherence and interpreting scores - Less evidence available for adolescents compared to adults - Need for further research on barriers for adults - Need for further research on intentional vs. unintentional nonadherence in adolescents - Need for further research on interaction of thinking styles, psychological disorders, and coping strategies on medication adherence	1. Treatment adherence

				implementing these strategies.	mnemonic for establishing good communication, information, simple regimen, overcoming barriers, focusing on the modifiable, and multimethod evaluation.	activity and a higher risk of relapse.				
Patients' Educational Program Could Improve Azathioprine Adherence in Crohn's Disease Maintenance Therapy	Lei Wang [22]	- Determine the risk factors of nonadherence to azathioprine (AZA) maintenance therapy for Crohn's disease (CD). - Evaluate the influence of patients' educational program on adherence to AZA maintenance therapy.	Single-center, cross-sectional, observational study	- Enrolled patients receiving AZA for CD maintenance therapy and collected demographic, clinical, and usage data. - Used univariate and multivariate analyses to identify risk factors for nonadherence. - Assessed adherence using the Medication Adherence Report Scale (MARS). - Evaluated beliefs about AZA using the	- AZA educational program - Duration: October 2018 to September 2019 (approximately 1 year) - Components: face-to-face classes, online classes, WeChat push - Conducted by: physicians - Content: medical treatment and surveillance of CD under AZA treatment - Participants: nonadherent patients	- Nonadherence to azathioprine (AZA) maintenance therapy for Crohn's disease (CD) occurred in 43.9% of patients. - Risk factors for nonadherence included young age, high education level, alcoholism, anxiety, depression, and low necessity	Adherence to AZA maintenance therapy, Endoscopic relapse rate, Clinical relapse rate	What are the risk factors for nonadherence to azathioprine maintenance therapy in Crohn's disease patients, and how do patients' educational programs influence adherence?	- Single-center study - Lack of long-term follow-up - Low participation rate in the educational program among nonadherent patients - Need for multicenter research with larger patient numbers and longer follow-up	1. Treatment adherence

				Beliefs about Medicines Questionnaire (BMQs). - Assessed AZA knowledge with a 10-item questionnaire. - Conducted an educational program and compared outcomes between educational and noneducational groups. - Performed statistical analysis using SPSS 22.0, including logistic regression.		belief and AZA knowledge. - An educational program improved necessity belief, AZA knowledge, and adherence, and reduced concern belief and endoscopic relapse rates.				
Disease-Specific Knowledge, Coping, and Adherence in Patients with Inflammatory Bowel Disease	Moradkhani, Anilga [85]	- Investigate the relationship between IBD-related knowledge and the use of coping strategies. - Investigate the relationship	- Non-randomized - Non-controlled - Observational study - Convenience sampling - Online recruitment - Single platform	- Participants were recruited online from IBD support groups using convenience sampling. - Standardized questionnaires were used to assess IBD knowledge	Not mentioned (the study does not describe any specific intervention being tested or administered to participants)	- Greater IBD knowledge is associated with more adaptive coping strategies, including active coping, instrumental support, planning, and	Coping strategies (active coping, planning, instrumental support, emotional support), Medication adherence	What is the relationship between IBD-related knowledge and the use of coping strategies and medication adherence in	- Potential for uncontrolled confounding variables (e.g., endoscopic severity, psychological distress) - Lack of control for all clinico-demographic variables	1. Treatment adherence

		between IBD-related knowledge and medication adherence.	(online) - Non-retrospective	(CCKNOW), coping strategies (Brief COPE scale), and medication adherence (Morisky Adherence Scale). - A 17-item questionnaire was used to collect demographic and clinical data. - Pearson correlation was used to examine relationships between IBD knowledge and coping strategies and medication adherence. - Multivariate regression and one-way ANOVA tests were used to assess potential confounding variables. - Statistical analyses were		emotional support. - There is no significant relationship between IBD knowledge and medication adherence. - Providing disease-related patient education may enhance coping in patients with IBD.		individuals with IBD?	associated with coping - Small sample size - Use of general scales not specific to IBD (Brief COPE, Morisky adherence scale) - Lack of a validated disease activity measure - Reliance on self-reported data without confirmation of physician-diagnosed IBD - Selection bias due to online recruitment method	

				performed using SPSS.						
Mapping conditional health literacy and digital health literacy in patients with inflammatory bowel disease to optimise availability of digital health information: a cross-sectional study	Sigurd Maurud [9]	- Examine IBD patients' health literacy and digital health literacy covariance with clinical, demographic and patient-reported outcomes. - Examine levels of HL and DHL in IBD patients and their associations with disease-specific and social conditions. - Provide insights to inform initiatives focused on improving health outcomes and reducing health disparities in IBD care.	Cross-sectional study, observational, single-site	- Cross-sectional study design - Canonical correlation analysis (CCA) to identify covariance between health literacy and digital health literacy dimensions and clinical, demographic, and patient-reported characteristics - Hierarchical clustering analysis to identify subgroups - Bivariate tests and logistic regression for cluster membership analysis - Use of specific questionnaires for health literacy, digital health literacy, quality of life,	- 5-ASA: 19.47% of participants (69 UC patients, 5 CD patients) - Biological treatment: 78.42% of participants (112 UC patients, 186 CD patients) - Immunosuppressants: 4.21% of participants (6 UC patients, 10 CD patients) - Corticosteroids: 8.68% of participants (24 UC patients, 9 CD patients)	- The study identified two patient clusters with distinct levels of health literacy and digital health literacy, with the lower HL/DHL group having lower self-efficacy, health status, and longer disease duration. - Disease activity and biological treatment were the strongest predictors of cluster membership, indicating their significant role in determining HL and DHL levels. - The	Health literacy (HL) and digital health literacy (DHL) covariance with clinical, demographic, and patient-reported outcomes	How do health literacy and digital health literacy in patients with inflammatory bowel disease relate to their clinical, demographic, and patient-reported outcomes?	- Imputation may have distorted the natural data structure. - Sample from a tertiary centre may not be representative of the entire IBD population. - Overrepresentation of CD patients in the sample. - Potential underrepresentation of UC patients due to less need for healthcare follow-up. - Self-reported medication adherence may overestimate actual adherence. - Cross-sectional design does not allow for causal inferences. - Clustering approach	1. Treatment adherence

				self-efficacy, illness perception, and medication adherence - Data supplementation with demographic and clinical information from electronic health records - Imputation of missing values using k-nearest neighbors - Standardization of continuous variables		findings emphasize the necessity of addressing clinical characteristics alongside HL and DHL in the dissemination of IBD health information to improve patient outcomes.			provides implicative rather than absolute results. - Further research needed for generalizability and understanding patient needs.	
Patients' knowledge of pregnancy-related issues in inflammatory bowel disease and validation of a novel assessment tool ('CCPKnow')	C. P. Selinger [21]	- Develop and validate the Crohn's and Colitis Pregnancy Knowledge Score (CCPKnow) questionnaire. - Evaluate the use of CCPKnow in a mixed community and hospital-based cohort	Observational study; mixed community and hospital-based setting; self-administered questionnaire; no randomization or control group; postal survey component	- Developed CCPKnow using feedback from patients, nurses, and gastroenterologists. - Validated discriminatory ability using four groups of hospital staff with different IBD education levels. - Evaluated internal	Administration of the Crohn's and Colitis Pregnancy Knowledge Score (CCPKnow) questionnaire to female patients with IBD to assess knowledge of pregnancy-related issues.	- The CCPKnow tool demonstrated excellent test characteristics and reliability in assessing patient knowledge of pregnancy-related issues in IBD. - Nearly half of	Crohn's and Colitis Pregnancy Knowledge Score (CCPKnow)	What is the level of knowledge about pregnancy-related issues among women with inflammatory bowel disease (IBD), and how does this knowledge	- Selection bias due to low postal return rate (28%) - Overrepresentation of patients with good knowledge - Potential underestimation of poor knowledge in unselected women with IBD - Future research needed	1. Treatment adherence

		to test the hypothesis that a large proportion of women with IBD have poor knowledge of pregnancy-related issues.		consistency using Cronbach's alpha and readability using Flesch Reading Ease and Flesch-Kincaid Grade Level. - Assessed construct validity by correlating with CCKnow in a cohort of women with IBD. - Recruited 145 women with IBD from hospital clinics and office-based gastroenterologists. - Collected patient demographics and disease specifics. - Used statistical analyses including student's t-test, ANOVA, and multiple linear regression.		the women with IBD had poor knowledge about pregnancy-related issues, indicating a pressing need for better education. - Better knowledge was associated with factors such as Caucasian ethnicity, higher income, having a partner, having children, and Crohn's and Colitis Association membership.		impact their decisions and behaviors regarding fertility, pregnancy, and medication use?	on patient adherence to medication during pregnancy - Future comparative studies on practitioner knowledge	

Future Directions Current Landscape of Telemedicine Practice in Inflammatory Bowel Disease	Patil, Seema A [86]	- Increase access to specialty IBD care - Facilitate early intervention for symptoms and signs of active inflammation - Reduce outpatient clinic burdens - Improve adherence to medication - Augment patient education - Decrease cost of care - Address geographic access issues - Centralize access to multiple providers - Understand patient engagement with telemedicine systems - Evaluate effectiveness	- IBD HAT: Pilot study (not controlled) - UC HAT: Randomized controlled trial - TELE-IBD: Multicenter randomized controlled trial - Constant Care: Randomized controlled trial - myIBDcoach: Randomized controlled trial - Pilot study in adolescents: Pilot study (not controlled) - eIBD program: Retrospective study - University of Maryland telehealth visits: Retrospective study -	- Remote monitoring methods include hardware, web-based programs, and mobile device applications. - Specific systems: IBD HAT, UC HAT, TELE-IBD, Constant Care, eIBD, myIBDcoach. - Trial designs: Randomized controlled trials, pilot studies. - Data collection: Patient symptoms, medication adherence, disease activity, quality of life.	- IBD Home Automated Telemanagement (IBD HAT): Weekly testing over 6 months. - TELE-IBD: Text messaging, weekly or every other week monitoring. - Constant Care: Web-based system, tested in a randomized controlled trial. - eIBD: Web-based program for inputting disease activity and quality of life information. - myIBDcoach: Web-based and HTML application formats for telemonitoring. - Telehealth visits: Alternative to office visits for follow-up care.	- Telemedicine is associated with improved quality of life, patient knowledge, and reduced healthcare utilization in IBD patients. - Specific telemedicine systems like IBD HAT and TELE-IBD have shown improvements in disease activity and patient satisfaction. - Telemedicine can address geographic and cost barriers to specialty care, improving access and efficiency.	- Disease activity (measured by Seo index) - Disease-specific quality of life - Adherence to medication - Patient satisfaction - Healthcare utilization (outpatient visits, hospitalizations) - IBD-related hospitalizations	How can telemedicine be optimized to improve outcomes for patients with inflammatory bowel disease by addressing barriers and enhancing access to specialty care, patient self-management, and education?	- Methodological concerns: Early studies were uncontrolled and had short durations. - High attrition rates in several studies. - Lack of long-term studies on remote monitoring. - Limited understanding of which patients benefit most from remote monitoring and which systems are most effective. - Barriers to widespread adoption: provider resistance, licensure issues, reimbursement challenges, liability concerns, and informed	1. Treatment adherence

		of different telemedicine systems	Dartmouth-Hitchcock IBD program: Study design not explicitly mentioned						consent requirements.	
Development and validation of a questionnaire to test Chinese patients' knowledge of inflammatory bowel disease	Huabing Xie [53]	- Develop and validate a questionnaire to assess IBD-related knowledge. - Analyze the factors affecting patients' knowledge of IBD. - Develop a short IBD knowledge questionnaire with different versions for CD and UC. - Investigate the factors that influence IBD patient knowledge.	- Multi-site - Prospective - Cross-sectional - Observational	- Developed and validated a questionnaire to assess IBD-related knowledge among Chinese patients. - Used the Delphi method for expert consultation to evaluate and modify the questionnaire. - Conducted a pilot study with 41 subjects to refine the questionnaire based on patient feedback. - Recruited 709 patients across multiple IBD centers using an online platform. - Performed statistical	Not applicable (the paper focuses on the development and validation of a questionnaire, not on a specific intervention applied to participants)	- The IBD knowledge questionnaires developed in the study have good reliability and validity for assessing patient knowledge of the disease. - Factors such as disease type, age, BMI, education level, income, treatment cost, disease duration, and frequency of visits significantly affect IBD patients' knowledge. - CD patients were found to	- Reliability of the IBD knowledge questionnaire (internal consistency, split-half reliability, test-retest reliability) - Validity of the IBD knowledge questionnaire (correlation between doctor score, IBD-KNOW score, and the first version of the questionnaires) - Total scores of the UC and CD knowledge questionnaires (points)	How can we develop and validate a questionnaire to assess inflammatory bowel disease (IBD)-related knowledge and identify factors affecting patient knowledge?	- The questionnaire was conducted online, and patients not in the IBD patient group were not included, which may impact representability. - Lack of comprehensive information on IBD, such as items related to surgery, reproduction, and family planning.	1. Treatment adherence

				analysis using SPSS 26.0 to assess reliability and validity. - Used multiple linear regression to identify factors influencing patient knowledge.		have better knowledge of IBD compared to UC patients. - Face-to-face education, books/manuals, and special lectures are the main sources of IBD knowledge for patients. - There is a significant association between medication adherence and disease knowledge among IBD patients.				
Feasibility and Acceptance of a Home Telemanagement System in Patients with Inflammatory Bowel Disease: A 6-	Cross, Raymond K [33]	- Assess the feasibility of a home telemanagement system (HAT) for IBD over a 6-month period. - Assess patient acceptance of	- Pilot study - Non-controlled - Non-randomized - Non-double-blind - Non-placebo-controlled - Multi-site (University	- Recruitment of adult IBD patients from specific medical centers. - Initial 30-40 minute training session at home to operate the HAT. - Weekly self-testing	- Home Telemanagement System (HAT) consisting of a laptop and a scale - Weekly self-testing sessions for 6 months - Responding to questions about IBD symptoms	- The Home Telemanagement System (HAT) was found to be feasible and accepted by patients with Inflammatory Bowel Disease	- Adherence with weekly self-testing - Patient acceptance (qualitative assessment using attitudinal surveys) - Clinical	How feasible and acceptable is a home telemanagement system for patients with inflammator	- Lack of a control group - Inability to blind patients to the intervention - Use of a non-validated modified disease activity index for UC and	1. Treatment adherence

Month Pilot Study		the HAT for IBD over a 6-month period.	of Maryland IBD Program and the gastroenterology clinic of the Veteran's Affairs Medical Center, Baltimore)	sessions over 6 months, alongside usual IBD care. - Assessment of feasibility through adherence to self-testing. - Evaluation of patient acceptance using attitudinal surveys. - Collection of demographic and clinical data at baseline and 6 months. - Use of descriptive statistics and paired t-tests for analysis.	and medication side effects - Daily educational prompts and questions - Secure web portal for clinician review and clinical alerts - Initial 30-40 minute instruction session - Continued usual IBD care alongside HAT sessions	(IBD), with high adherence rates and positive patient feedback. - Improvements in disease activity and quality of life were observed, although not statistically significant, indicating a trend towards better outcomes. - Patient knowledge about IBD significantly increased during the study.	disease activity (Harvey Bradshaw index and laboratory data) - Disease-specific quality of life (Short Inflammatory Bowel Disease Questionnaire) - Patient IBD knowledge (Crohn's and Colitis Knowledge Questionnaire) - Patient satisfaction with health-care services (Client Satisfaction Questionnaire)	y bowel disease?	indeterminate colitis - Unable to compare costs with routine care - Possible regression to the mean	
Trusted evidence. Informed decisions. Better health. Cochrane	Morris Gordon [61]	- To identify the different types of educational interventions for IBD. - To	Systematic review of randomized controlled trials (RCTs) comparing	- Conducted a systematic review of 14 studies with 2708 participants. -	- Seminars - Information booklets - Text messages - E-learning - Multi-professional	- Patient education combined with standard care is probably	quality of life (QOL), functioning in typical life activities, health-	What are the different types of educational interventions for	- Risk of bias across all studies - Limited homogeneous reporting of	1. Treatment adherence

Database of Systematic Reviews [Intervention Review] Patient education interventions for the management of inflammatory bowel disease		determine how these interventions are delivered. - To assess the effectiveness and safety of these interventions for people with IBD.	patient education interventions to standard care or no intervention.	Used Review Manager Web for data analysis. - Assessed risk of bias and evidence certainty using GRADE methodology. - Employed random-effects models for data synthesis. - Planned subgroup and sensitivity analyses. - Followed Cochrane Handbook guidelines.	group-based program - Guidebooks - Staff-delivered program based on an illustrated book - Standardized program followed by group session - Lectures alternating with group therapy - Educational sessions based on an IBD guidebook - Internet blog access and text messages - Structured education program - Interactive videos - 3-week educational program with 2-hour sessions - 12-hour program over four weeks - 6-month program - Video presentation (5 minutes) - Educational booklet -	equivalent to standard care in reducing disease activity, flare-ups/relapse, and improving quality of life in patients with IBD. - There is no significant benefit of patient education over standard care for these outcomes. - Further research on these outcomes is not indicated due to sufficient current evidence.	related quality of life (HRQoL) using IBDQ, coping ability using Sense of Coherence scale (SOC), health service resource use, patient satisfaction	inflammatory bowel disease (IBD), how are they delivered, and what is their effectiveness and safety in managing IBD?	outcomes - Lack of detailed information about educational components and standard care - Need for further research on specific areas like healthcare access and medication adherence - Inability to perform subgroup and sensitivity analyses due to lack of data	

					Behavioral interventions (therapeutic scheme and pharmaceutical guidance) - SMS messages (reminder and motivational)					
EHealth Technologies in Inflammatory Bowel Disease: A Systematic Review	Jackson, Belinda D. [5]	- Evaluate the impact of eHealth technologies on conventional clinical indices and patient reported outcome measures (PROs) in IBD. - Assess the effectiveness, cost-effectiveness and feasibility of using eHealth technologies to facilitate the self-management of individuals with IBD. - Provide	Systematic review of 6 randomized controlled trials and 9 observational studies	- Conducted a systematic review of literature on eHealth technologies in IBD. - Used multiple databases (Medline, EMBASE, Cochrane Controlled Trials Register) and included abstracts from international conferences and internet publications. - Employed specific search terms related to eHealth and IBD. - Two reviewers independently	- Web-based interventions - Telemedicine using video-link - Virtual clinics with annual blood tests and questionnaires - Smartphone apps with fecal calprotectin kits - Email communication	- EHealth technologies improve clinical indices and patient-reported outcomes in IBD, including reduced relapse duration, improved disease activity, medication adherence, quality of life, and healthcare costs. - Virtual clinics are cost-effective and facilitate remote	disease activity, quality of life, medication adherence, work productivity, cost-efficacy, depression, anxiety, and IBD knowledge	What is the impact of eHealth technologies on conventional clinical indices and patient-reported outcomes in inflammatory bowel disease (IBD), and how effective, cost-effective, and feasible are they for facilitating self-management?	- Methodological shortcomings: heterogeneity of outcome measures, lack of clinician/patient input, lack of validation against conventional clinical indices and PROs, limited cost-benefit analyses - Reliability issues with self-reporting of symptoms - Lack of formal evaluation of self-management using validated indices - Need for high	1. Treatment adherence

		recommendations for their design and optimal use for patient care.		identified eligible studies and resolved discrepancies through discussion. - Assessed risk of bias in RCTs using Cochrane handbook criteria and in observational studies using the Effective Public Health Practice Project tool. - Categorized studies by type of eHealth technology used. - Initially identified 695 articles, with 112 remaining after removing duplicates and screening titles and abstracts. - Excluded 86 articles based on predefined quality parameters.		management of up to 20% of IBD patients. - The success of eHealth interventions relies on high adherence and low attrition rates, with self-reporting reliability being a critical factor.			adherence and low attrition rates - Lack of structured framework for planning and implementing eHealth interventions - Important PROs like fatigue and disability not assessed - Need for validation of social media metrics as PROs - Impact of email communication on disease activity and PROs not assessed - Limited effectiveness of telemedicine due to small sample sizes and heterogeneous outcomes - Mobile applications lack validated clinical indices and decision support -	

									Medication adherence and patient knowledge outcomes not reported for mobile applications	
Self-care in patients affected by inflammatory bowel disease and caregiver contribution to self-care (IBD-SELF): a protocol for a longitudinal observational study	Daniele Napolitano [49]	- Describe patient engagement in self-care and caregiver contributions. - Identify patient, caregiver, and dyadic predictors of self-care and investigate the mediating effects of self-efficacy. - Determine whether patient self-care and caregiver contribution influence outcomes for both dyad members. - Assess the impact of	- Multicentre - Longitudinal - Observational - 1:1 randomization strategy for balancing sample sizes between UC and CD groups	- Multicentre longitudinal observational study - Recruitment of 250 patients with IBD and their caregivers from 9 IBD units in Italy - Data collection at baseline, 6 months, and 12 months - Use of multivariable regressions, path analyses, and structural equation models - Dyadic analyses to control for interdependence - Pilot testing for questionnaire manageability - Eligibility	Not applicable (the study is observational and does not involve any specific intervention)	- The study aims to enhance understanding of the self-care process in the patient-caregiver dyad in IBD, aiding future educational interventions. - It investigates patient self-care and caregiver contributions to self-care in IBD, focusing on their impact on outcomes. - The study's findings are expected to improve educational	- Disease Severity (CRP ≤0.5 mg/dL and FCP level ≤250 μg/g) - Endoscopic measures (Mayo Endoscopic Score for UC and SES-CD for CD) - Number of hospital admissions - Quality of life (QoL) using Short Form-12 Questionnaire - Resilience using Connor-Davidson Resiliency Scale (CD-RISC 25) -	What are the dynamics and predictors of patient self-care and caregiver contributions in the context of inflammatory bowel disease (IBD), and how do these factors influence outcomes for both patients and caregivers?	- The study will only recruit participants from healthcare settings in Italy, limiting the capture of cultural differences from other countries. - The evaluation of data at specific time points (6-12 months) may lead to missed follow-ups. - The analysis of clinical scores requires outpatient visits, which may not always coincide with the study's timing, potentially affecting	1. Treatment adherence

		doctor and nurse involvement on patient-caregiver self-care and overall outcomes in IBD management.		criteria include age and diagnosis requirements - Data collection during outpatient visits with verbal and written consent		programs for IBD management by understanding self-care's role in symptom management, quality of life, and self-efficacy.	Positive feelings from caregiving using Positive Aspects of Caregiving scale		compliance with questionnaires.	
Higher levels of disease-related knowledge reduce medical acceleration in patients with inflammatory bowel disease	Park, Jihye [42]	To determine whether higher levels of disease-related knowledge reduce medical acceleration in patients with inflammatory bowel disease (IBD).	Prospective observational cohort study, single-site	- Used the validated IBD-KNOW questionnaire to assess disease-related knowledge. - Collected clinical data prospectively to analyze factors related to step-up therapy. - Defined step-up therapy as the new use of corticosteroids, immunomodulators, or biologics. - Conducted statistical analyses using means, medians,	- Corticosteroids: 57.7% in the continuous therapy group and 68.9% in the step-up therapy group. - Immunomodulators (including azathioprine, 6-mercaptopurine, methotrexate, cyclosporine): 44.7% in the continuous therapy group and 46.7% in the step-up therapy group. - Biologics (including anti-tumor necrosis factor-alpha (TNFα) and anti-$\alpha 4\beta 7$-integrin	- Higher levels of disease-related knowledge in patients with IBD are associated with a reduced need for step-up therapy. - The IBD-KNOW score is independently predictive of step-up therapy, indicating that knowledge is a significant factor in determining	Step-up therapy; Medical acceleration	Does higher disease-related knowledge reduce medical acceleration in patients with inflammatory bowel disease?	- Assessment of disease-related knowledge was conducted at only a single time-point. - Relatively short follow-up duration did not allow for evaluation of long-term outcomes. - Drug compliance and adherence were not investigated. - Average disease duration of the cohort was relatively shorter.	1. Treatment adherence

				and proportions. - Used Kaplan-Meier and Cox proportional hazards analyses to evaluate the impact of IBD-KNOW scores on medical acceleration. - Collected baseline clinical characteristics from electronic medical records.	agents): 21.2% in the continuous therapy group and 18.9% in the step-up therapy group. - Biologics dose escalation: part of step-up therapy.	treatment needs. - Patients with higher IBD-KNOW scores are less likely to require step-up therapy, potentially reducing healthcare costs.				
Patients' information-seeking activity is associated with treatment compliance in inflammatory bowel disease patients	Valérie Pittet [39]	- To examine the association between information-seeking activity and treatment compliance among IBD patients. - To compare information sources and concerns between compliant and	Cross-sectional study; Observational study; Part of a national clinical cohort (SIBDC); Includes qualitative survey; Non-controlled; Non-randomized; Non-blinded	- Used data from the Swiss IBD cohort study and a qualitative survey. - Clinical data gathered through clinical reporting forms during medical visits. - Self-administered questionnaire sent to patients by mail. - Qualitative survey conducted to investigate	- Antibiotics or topical steroids (metronidazole, ciprofloxacin, topical steroids, budesonide) - 5-aminosalicylates (5-ASA) (oral and/or topical 5-ASA, sulfasalazine) - Oral steroids (prednisone, systemic steroids) - Immunosuppressive drugs (methotrexate, azathioprine, 6-	- IBD patients noncompliant with treatment were more likely to seek disease-related information than compliant patients. - The odds ratio for noncompliance among information seekers was significantly	Noncompliance to treatment (binary variable: compliant vs. noncompliant)	What is the association between information-seeking activity and treatment compliance among inflammatory bowel disease patients?	- Use of self-reported patient questionnaires for compliance measurement - Potential recall bias in self-reported data - Lack of objective measures like serum drug levels for all treatments - Selection bias due to high non-response rate - Differences in characteristics	1. Treatment adherence

		noncompliant patients.		information sources and themes. - Focused on noncompliance to treatment as the main outcome. - Studied information-seeking during disease activity and remission. - Themes of information sought collected through open-ended questions. - Sources of information identified using a pre-established list. - Statistical analysis conducted using STATA software. - Ethical approvals obtained from regional Swiss Ethics Committees.	mercaptopurine) - Biologicals (infliximab, adalimumab, certolizumab) - Three-quarters of patients were under immunosuppressive drugs or 5-ASA - One out of five patients was under biological therapy - About 42% of patients took at least two concomitant medications	higher, indicating a strong association between information-seeking and noncompliance. - Compliant patients more frequently consulted general practitioners and used books or television for information, while noncompliant patients were more interested in tips for daily disease management.			between respondents and non-respondents - Potential unaccounted confounders related to patient perceptions of drug benefits and risks	

What do patients need to know? Living with inflammatory bowel disease	Katarina P Lesnovska [70]	To explore the need for knowledge as expressed by patients diagnosed with inflammatory bowel disease.	- Study type: Qualitative observational study - Method: Inductive qualitative interviews - Analysis: Qualitative inductive content analysis - Participants: 30 patients selected from an outpatient clinic in south-eastern Sweden - Selection criteria: Maximum variation in age, sex, diagnosis, duration of IBD, and history of previous surgery - Single-site study - No randomization, blinding, or control	- Inductive qualitative interviews with 30 patients. - Content analysis to explore knowledge needs. - Interviews conducted in hospital or at patients' homes, digitally recorded and transcribed verbatim. - Ethical approval from Regional Ethics Committee and adherence to Helsinki Declaration.	- Medical maintenance treatment: 29 patients (97%) - Surgical procedures: 11 patients (37%) - Stoma: 1 patient (3%) - Telephone contact with a nurse: Not specified frequency or duration	- The study identified three main categories of knowledge needs for IBD patients: understanding the disease course, managing everyday life, and difficulties with understanding information. - Patients primarily needed knowledge about the disease course to manage their daily lives effectively. - There was significant variation in knowledge needs among patients, with the greatest needs observed	- Knowledge related to the course of the disease - Knowledge related to managing everyday life - Difficulty understanding and assimilating information	What are the knowledge needs of patients diagnosed with inflammatory bowel disease?	- Confusion between the concepts of "knowledge" and "information" during interviews - Lack of clarification on the difference between "knowledge" and "information" at the start of interviews	1. Treatment adherence

			groups mentioned			immediately after diagnosis and during relapse.				
Importance of Patients' Knowledge of Their Prescribed Medication in Improving Treatment Adherence in Inflammatory Bowel Disease	Chung Hyun Tae [20]	- Investigate the association between treatment nonadherence and patients' knowledge of the prescribed medication among individuals with inflammatory bowel disease (IBD). - Evaluate the impact of nonadherence on relapse.	Prospective observational cohort study; nested case-control method	- Prospective observational cohort study - Self-reported survey at baseline for demographic data, knowledge of prescribed medication, and adherence factors - Adherence measured using Morisky Medication Adherence Scale-4 - Knowledge of prescribed medication assessed using a valid tool - Disease activity measured using Crohn's Disease Activity Index (CDAI) and partial Mayo score (PMS) - Statistical	- 5-aminosalicylates: 65.2% of participants - Corticosteroids: 16.7% of participants - Immunomodulators: 40.6% of participants - Biological agents: 13.0% of participants - Median of 2 treatments prescribed per patient	- Nonadherence was observed in 36.2% of the subjects and was significantly associated with younger age, longer intervals between outpatient clinic visits, and limited knowledge of prescribed medication. - These factors increased the risk of clinical relapse by 2.9-fold. - The study emphasizes the importance of patient knowledge and regular	Nonadherence to treatment, Risk of clinical relapse (relative risk)	What is the association between patients' knowledge of their prescribed medication and treatment adherence in inflammatory bowel disease (IBD), and how does this association impact the risk of relapse?	- Adherence was categorized into two groups (nonadherent vs. adherent) using an arbitrary cutoff, which may not accurately reflect the continuous nature of adherence. - The use of a self-administered questionnaire may lead to overestimation of adherence due to social desirability bias.	1. Treatment adherence

				analysis included t-tests and chi-square tests - Follow-up for 18 months to assess relapse		follow-up visits in improving treatment adherence and reducing relapse risk.				
E-health empowers patients with ulcerative colitis: a randomised controlled trial of the web-guided 'Constant-care' approach	Margarita Elkjaer [35]	- To investigate the feasibility of a self-administered, web-based e-health treatment program for patients with ulcerative colitis. - To determine if the e-health approach improves patient compliance, knowledge, QoL, disease outcomes, and safety. - To investigate if the e-health approach decreases healthcare costs	Randomized controlled trial (RCT), multi-site (Denmark and Ireland), historical control group included	- Randomized controlled trial with 333 patients with mild/moderate ulcerative colitis. - Patients randomized into web-group using web-based e-health treatment or control group with usual care. - Historical control group included for comparison. - Web-group received disease-specific education and self-treatment guidance via website. - Daily logging and disease activity scoring during relapses. -	- Web-guided approach via http://www.constant-care.dk for 12 months - Acute systemic treatment with 4 g daily of 5-ASA for up to 28 days, with option to extend for another 28 days - Additional treatment with topical 5-ASA and/or prednisolone as chosen by the patient	- The web-guided 'Constant-care' approach was preferred by 88% of web patients and increased adherence to acute treatment by 31% in Denmark and 44% in Ireland. - Median relapse duration was significantly shorter in the web group (18 days) compared to the control group (77 days). - The approach was found to be	- Feasibility of the web-based approach - Patient compliance - Knowledge - Quality of life (QoL) - Disease outcomes - Safety - Healthcare costs	Can a web-based e-health treatment program improve patient compliance, knowledge, quality of life, disease outcomes, and safety, while reducing healthcare costs for patients with ulcerative colitis?	- Uncertainty about long-term effects on the natural disease course of ulcerative colitis - Low compliance with faecal samples in Irish patients, limiting validation of over-treatment - Potential misunderstanding due to study design regarding faecal sample importance - Variations in healthcare systems and patient mentality affecting generalizability	1. Treatment adherence

		primarily in Denmark.		Intention to treat analysis using log-rank, chi-square, Fisher's exact, and Student t-tests. - Aims: feasibility, compliance, knowledge, quality of life, disease outcomes, safety, healthcare costs.		feasible, safe, and cost-effective, reducing outpatient visits and resulting in a saving of 189 euros per patient per year.				
'Simpleness': a qualitative description study exploring patient perspectives on the barriers and facilitators of using digital health tools to self-manage inflammatory bowel disease	Lekan Ajibulu [64]	To explore patient perspectives on the barriers and facilitators associated with using digital health tools for IBD self-management, focusing on the adoption of a tool called MyIBDToolkit.	Qualitative description approach; observational study; semi-structured interviews; thematic saturation; participatory design with Patient Advisory Council (PAC) involvement	- Qualitative description approach - Virtual semi-structured interviews - Thematic analysis with member checking - Adherence to COREQ checklist - Participant recruitment from IBD clinics and online - Interviews conducted until thematic saturation -	MyIBDToolkit, a digital health tool for IBD self-management, demonstrated during interviews with features like symptom tracking, mental health resources, and dietary education. No specific frequency, duration, or dose mentioned.	- Patients view digital health tools like MyIBDToolkit as beneficial for disease monitoring and care coordination. - Significant barriers include data entry burden, privacy concerns, and variability in healthcare provider use. - Addressing these barriers	Patient perspectives on barriers and facilitators of using MyIBDToolkit for IBD self-management, focusing on usability and acceptability.	What are the potential barriers and facilitators for using MyIBDToolkit for the management of IBD?	- The predominance of participants of European ancestry limits the generalizability of the findings to more diverse populations. - The study did not include individuals with IBD who receive care at general gastroenterology clinics, limiting relevance for those without	1. Treatment adherence

				Transcription and verification of recordings - Line-by-line coding using NVivo 14 - Trustworthiness ensured through member checking and peer debriefing		and incorporating patient feedback is crucial for enhancing the effectiveness and acceptability of digital health tools in IBD management.			access to IBD specialists. - Potential for interviewer and responder bias in face-to-face interviews.	
Does patient knowledge affect the colorectal cancer risk in ulcerative colitis?	Eaden, Jayne [41]	- Investigate the relationship between knowledge about ulcerative colitis and its cancer risk. - Examine the relationship between knowledge about IBD and the development of colorectal cancer using the CCKNOW score.	Retrospective case-control observational study	- Retrospective study design - Mailed 24-item CCKNOW questionnaire to cases (colorectal cancer complicating ulcerative colitis) and controls (ulcerative colitis without cancer) - Controls matched for age and sex - Independent two-sample t-tests for mean score comparison -	The CCKNOW questionnaire was administered once to participants.	- The CCKNOW scores were comparable in cases and controls, indicating no significant difference in knowledge about ulcerative colitis and its cancer risk between those who developed colorectal cancer and those who did not. - Membership	CCKNOW score; Correlation between CCKNOW score and stage of colon cancer at diagnosis	Is there an association between patient knowledge about ulcerative colitis and the risk of developing colorectal cancer?	- small sample size - potential bias due to lack of data on deceased patients - need for further data on disease duration and severity - retrospective design may not accurately reflect knowledge levels at diagnosis - need for prospective studies to prevent learning effects - suggestion to	1. Treatment adherence

				Logistic regression for association analysis - Response rates: 60% for cases, 44% for controls		in the National Association of Crohn's and Colitis (NACC) was associated with higher CCKNOW scores, but adjusting for this factor did not reveal an association between CCKNOW scores and cancer development. - There was no correlation between the CCKNOW score and the stage of colon cancer at diagnosis.			use compliance measures like missed appointments	
TELEmedicine for Patients with Inflammatory Bowel Disease (TELE-IBD): Design and implementati	Raymond K Cross [56]	- Compare disease activity and quality of life over 1 year in a randomized trial of IBD patients receiving	- Multicenter - Randomized - Clinical trial - Parallel design - Controlled - Stratified randomizatio	- Multicenter, randomized clinical trial over 12 months. - Participants divided into intervention groups (weekly or every other	- TELE-IBD system: weekly or every other week assessment of symptoms, side effects, weight, and delivery of medication prompts and	- The study aims to determine if telemedicine decreases disease activity and improves quality of life	Disease activity and quality of life scores	How can telemedicine improve disease activity and quality of life for patients with inflammator	- Focus on patients from tertiary referral centers may limit generalizability to community settings. - Potential for	1. Treatment adherence

on of randomized clinical trial		standard care versus telemedicine. - Determine if telemedicine decreases disease activity and improves quality of life compared to standard IBD care.	n by disease type and disease activity	week telemedicine) and a control group (standard care). - Primary outcomes: changes in disease activity and quality of life. - Recruitment from University of Maryland Baltimore, University of Pittsburgh Medical Center, and Vanderbilt University. - Inclusion criteria: documented IBD with recent flare-ups. - Stratified randomization by disease type and activity. - Regular self-testing and monitoring of symptoms, side effects, and medication adherence.	education via texts to the participant's mobile phone. - 45-minute training session at baseline visit to operate the TELE-IBD system. - Customized alerts and action plans based on participant responses. - Educational tips: twice weekly (weekly self-assessment group) or every week (every other week self-assessment group). - Standard care in addition to telemedicine intervention.	compared to standard IBD care. - Telemedicine is hypothesized to result in decreased disease activity and improved quality of life by enhancing adherence, monitoring, and self-management. - The study expects that better control of IBD through telemedicine will lead to reduced urgent care visits, hospitalizations, and healthcare costs.		y bowel disease compared to standard care?	higher attrition rates could impact ability to detect significant differences. - Exclusion of mucosal healing due to cost constraints. - Telemedicine might be more effective in community settings. - High attrition rates in previous telemedicine trials.	

Challenges in the design of a Home Telemanagement Trial for patients with ulcerative colitis	Cross, Raymond [59]	- Primary objectives: Decrease disease activity, improve quality of life, promote medical adherence, and decrease utilization of healthcare resources. - Secondary objectives: Evaluate differences in urgent care visits, hospitalizations, and length of stay. - Tertiary objectives: Assess impact on depression scores, patient knowledge, patient satisfaction, and patient-provider discordance.	- Randomized - Controlled - Randomized controlled trial - Not double-blind (subjects not blinded, staff masked) - Stratified (based on disease activity at baseline) - Random permuted block design with randomly varied block sizes - Not multi-site (conducted at University of Maryland School of Medicine and Veterans Affairs Maryland Health Care System, Baltimore)	- Randomized controlled trial with 100 patients. - Intervention arm: Weekly self-testing with telemanagement system. - Control arm: Best available care with educational materials and written action plans. - Outcome measures: Disease activity (Seo index), quality of life (Inflammatory Bowel Disease Questionnaire), adherence (pharmacy refill data and Morisky Medication Adherence Scale), healthcare resource utilization. - Randomization: Permuted block design with	- Telemanagement system consisting of a home unit with a scale and laptop, a decision support server, and a web-based clinician portal. - Weekly self-testing with the telemanagement system. - Customized action plans based on self-testing responses. - Educational curriculum with daily tips and questions. - Ability to send electronic messages to the nurse coordinator at any time.	Not mentioned (the paper describes the design and challenges of the trial but does not provide results or conclusions)	- Disease activity (Seo index) - Quality of life (Inflammatory Bowel Disease Questionnaire) - Medical adherence rates	Can telemanagement improve symptoms, quality of life, adherence, and reduce healthcare costs in patients with ulcerative colitis compared to best available care?	- Results may not be generalizable due to the tertiary center setting. - Subjects are not blinded to the intervention, potentially introducing bias. - The study population may not be representative of the general UC patient population due to the inclusion of patients with moderate to severe disease and economic disadvantages. - Nonblinding design may result in bias due to patient perceptions.	1. Treatment adherence

				stratification by disease activity at baseline. - Blinding: Research staff blinded to treatment allocation. - Statistical analysis: T-tests and Wilcoxon signed rank tests for comparing outcomes.						
Inflammatory bowel disease patient profiles are related to specific information needs: A nationwide survey	Daher, Saleh [76]	- Conduct a national survey of IBD patients' information needs. - Identify gaps in the information received. - Relate unique patient characteristics to specific information needs. - Evaluate the scope of patient self-knowledge. - Define unmet needs in	Observational study, national survey, multi-site	- Conducted a national survey of IBD patients' information needs. - Collected demographic and clinical information through a questionnaire. - Questionnaire completed online or at hospital clinics. - Used exploratory factor analysis to group related items into domains. - Calculated	- Mesalamine - Corticosteroids - Thiopurines - Methotrexate - Biologic agents	- IBD patients experience a significant information deficit at diagnosis, with most receiving little or no information on important topics. - Patients lack self-knowledge about their disease, which varies based on individual profiles. - Personalized	- Identification of gaps in information received by IBD patients - Association of patient characteristics with specific information needs	What are the specific information needs of inflammatory bowel disease (IBD) patients, and how do these needs vary based on unique patient characteristics?	- Surveys may be biased by who completes and does not complete them. - The findings are limited by the questions asked. - Larger, population-based studies are needed to further explore the links between patient characteristics and information needs.	1. Treatment adherence

		current practice. - Explore whether information needs differ in relation to patients' clinical and demographic characteristics.		average ratings for each domain. - Analyzed associations using patient profiles. - Conducted univariate and multivariate analyses. - Statistical analysis performed using SPSS software.		education resources may improve patient compliance and outcomes.				
Developing Mobile Health Applications for Inflammatory Bowel Disease: A Systematic Review of Features and Technologies	Parvin Akbarian [79]	The study aimed to identify the features and technologies used in the development of IBD disease management applications.	Systematic review following PRISMA guidelines	- Conducted as a systematic review following PRISMA guidelines. - Searched PubMed, Scopus, and Web of Sciences databases using specific keywords. - Used Newcastle-Ottawa Scale for quality assessment. - Data extraction involved a structured form	- Education: Programs on healthy lifestyle habits, nutrition, exercise, relaxation, and mental health. - Monitoring: Direct connection to the IBD registry; collecting patient-generated health data. - Counseling and treatment: Receiving medical consultations and treatment plans through the app. -	- All IBD management applications were focused on treatment, with 83% aimed at self-management and 33% at diagnosis. - The applications were categorized into four main feature groups: education, monitoring, counseling, and diagnosis	Self-management, quality of life, psychological distress, medication adherence	What features and technologies are used in mobile health applications for managing inflammatory bowel disease (IBD)?	- Review of only three databases, potentially missing relevant studies - Unavailability of full-text articles - Limited role in early diagnosis due to challenges in accurate diagnosis without clinical intervention - Regulatory concerns and privacy issues - Lack of official development	1. Treatment adherence

				for application details. - Descriptive analysis for summarizing findings. - Hand searches in references and Google Scholar. - Focused on original English-language articles reviewing or introducing IBD health applications.	Diagnosis and treatment: Assessing disease severity; using home tests for calprotectin levels.	and treatment. - Current applications have limited diagnostic capabilities but empower patients through education and monitoring, with potential for enhancement through AI and decision support systems.			standards for chronic disease management applications - Need for improvements in design, security, and accessibility - Difficult implementation on all types of smartphones - Security risks due to lack of data encryption	
Impact of the Mobile HealthPROMISE Platform on the Quality of Care and Quality of Life in Patients With Inflammatory Bowel Disease: Study Protocol of a Pragmatic	Ashish Atreja [44]	- Determine the impact of the HealthPROMISE app on improving outcomes (quality of care, quality of life, patient adherence, disease control, and resource utilization) compared to a	- Pragmatic randomized controlled trial - Single-center - Allocation ratio: 1:1 - Controlled (with an education app as the control intervention) - Intention-to-treat (ITT) analysis -	- Pragmatic randomized controlled trial (RCT) to evaluate the impact of HealthPROMISE app on quality of care and quality of life. - Participants recruited during face-to-face visits and randomized into HealthPROMIS	- Use of the HealthPROMISE app for self-monitoring and collaborative decision support. - Update information and receive feedback on QOL and resource utilization every 2 weeks. - Complete SIBDQ and EQ-5D questionnaires	Not mentioned (the paper is a study protocol and does not include results or conclusions)	Number of quality indicators met in HealthPROMISE versus control arm	Can a patient-centric self-monitoring and collaborative decision support platform improve quality of care, quality of life, patient adherence, disease	- Estimated 30% attrition rate - Potential non-use of the app by some participants - Limited generalizability to other platforms or settings	1. Treatment adherence

Randomized Controlled Trial		patient education app. - Evaluate if the HealthPROMISE platform leads to sustainable improvement in overall quality of life for IBD patients. - Assess significant improvements in quality of care metrics, quality of life, and resource utilization compared to the control group.	Per-protocol analysis	E or control (education app) arms. - HealthPROMISE arm: patients update information, receive disease summary, quality metrics, and QOL trend graphs. - Control arm: data entry at baseline, office visits, and end of study without decision support. - Recruitment goal: up to 300 patients with IBD, 1:1 allocation ratio. - Primary endpoint: number of quality indicators met in HealthPROMISE vs control arm. - Secondary endpoints: emergency visits, hospitalizations, QOL score	every 2 weeks. - Providers use a Web-based dashboard integrated with EHRs to view patient data. - Receive HealthPROMISE app PIN. - Monetary incentive of $25 for completing questionnaires.			control, and resource utilization in patients with Inflammatory Bowel Disease compared to a patient education app?		

				changes, proportion meeting quality metrics and disease control. - Data collection: SIBDQ, EQ-5D, symptom updates, quality indicators. - Statistical analysis: SAS for data processing, Pearson's chi-square tests, ANCOVA. - Interim analysis at 150 patients followed up for week 52. - Intention-to-treat and per-protocol analyses.						

Table S3B Self-management behaviours

Title	Author	Study objectives	Study design	Methodology	Intervention	Main findings	Outcome measured	Research question	Limitations	Domain
Inflammatory bowel disease nurses' views on taking on a new role to support an online self-management programme for symptoms of fatigue, pain and urgency: a qualitative study to maximise intervention acceptance	Chanais Matthias [84]	- To explore with IBD nurses what type of support they deem necessary for an online intervention. - To elicit stakeholders' perceptions on incorporating a new complex intervention into clinical practice. - To refine the proposed intervention based on feedback to minimize additional workload and clarify roles.	Qualitative study using semi-structured focus group interviews	- Semi-structured focus group interviews with IBD clinical and research nurses. - Interviews conducted to gather thoughts on an online self-management intervention for IBD. - Participant information collected using paper forms; interviews audio-recorded and transcribed verbatim. - Thematic analysis using NVivo 12 software, following Braun and Clarke's method. - Analysis involved systematic reading, line-by-line coding, and theme refinement.	- 12-session online self-management intervention using cognitive behavioural therapy principles over 3 months. - One mandatory 30-minute phone call with an IBD nurse facilitator at the initial stages. - Online messaging system for communication between facilitator and patient. - Facilitator training in communication skills and risk management.	- The main findings highlighted the need to minimize additional workload for IBD nurses, which was a significant concern among participants. - The study led to refinements in the intervention, including reducing scheduled patient contact, clarifying the facilitator's role, and introducing an online messaging system. - The importance of stakeholder input in refining interventions for long-term implementation was emphasized.	Not mentioned (the paper does not specify the primary outcomes or endpoints being measured)	What are the practicalities and challenges faced by IBD nurses in supporting patients undertaking an online self-management intervention, and how can these challenges be addressed to facilitate effective implementation?	- Dominant members in focus groups may influence discussions, potentially silencing less dominant participants. - Initial thoughts and perspectives can significantly influence the direction of discussions, potentially missing some concerns or benefits.	2. Self-management behaviours

Evaluation of an Ongoing Psychoeducational Inflammatory Bowel Disease Support Group in an Adult Outpatient Setting	Kristin Mcmaster [48]	- Evaluate client-rated satisfaction of the support group as an indicator for ongoing attendance. - Assess perceived social support from the support group in comparison to other sources of social support. - Conduct qualitative analysis of members' expectations of group and perceived benefit.	- Exploratory study - Observational study - Non-controlled - Non-randomized - Single-site study	- Sample: 18 adults who attended more than two meetings of an ongoing open psychoeducational IBD support group. - Questionnaires: Client Satisfaction Questionnaire (CSQ-8), Multidimensional Support Scale (MDSS), demographic questions, and open-ended qualitative questions. - Methods: Quantitative (CSQ-8, MDSS) and qualitative (open-ended questions) analysis. - Data collection: Over a period of four months, with options to complete the questionnaire at the meeting or at home. - Inclusion criteria: Age 18+, English literacy, attendance at least two	- Intervention: Ongoing psychoeducational IBD support group - Frequency: Monthly meetings - Duration: Each meeting consists of a 1-hour informative presentation and a 1-hour discussion - Components: Educational presentations by expert guest speakers and discussion groups moderated by a clinical social worker - Topics: Diet/nutrition, complementary and alternative medicine, coping/psychological issues, medication side effects, traveling with IBD, surgery, bone health, and pain management	- Participants reported high satisfaction with the support group, rating peer support higher than support from family, friends, or professionals. - The psychoeducational structure of the group was effective in meeting participants' expectations for mutual support and education. - The study suggests that combining educational components with support discussions is an ideal structure for IBD patients and their caregivers.	Client-rated satisfaction (CSQ-8), Perceived social support (MDSS), Qualitative analysis of members' expectations and perceived benefits	What are the benefits of attending an ongoing psychoeducational IBD support group in an adult tertiary setting?	- Lack of diversity in the sample (predominantly female and Caucasian) - Potential for reporter bias due to lead facilitator involvement - Exploratory study design without a control group - Small sample size from one region, not representative of the broader IBD population	2. Self-management behaviours

				support group meetings.						
Exploring the foundations of a digital health information service for patients with inflammatory bowel disease: a mixed method study in Gravitate-Health	Sigurd Maurud [78]	- Identify key aspects required for digital promotion of self-management in IBD patients. - Explore health information needs and preferences of both patients and healthcare professionals. - Determine how digital health information can be prioritized to support self-management.	- Mixed methods study - Retrospective observational study - Multi-site study - Purposive sampling - Qualitative data collection (semi-structured interviews and focus group interviews) - Retrospective analysis of electronic health record (EHR) summaries	- Mixed methods study combining qualitative and quantitative data. - Qualitative data: Semi-structured interviews with 17 IBD patients and 2 focus group interviews with 11 healthcare professionals. - Quantitative data: Audit of 1,481 electronic health record summaries from the IBD help line. - Purposive sampling used for participant selection. - Reflexive thematic analysis (TA) for qualitative data; frequency distributions and Pearson's chi-squared test for quantitative data. - Conducted in a gastroenterology department at a large university	- Biological treatments: infliximab, adalimumab, vedolizumab, and ustekinumab (received by 10 participants) - 5-aminosalicylic acid treatment (received by 6 participants)	- The study identifies key aspects for developing a digital health information service for IBD patients, focusing on their needs for practical information about the disease, treatment, and self-management. - Both patients and healthcare professionals agree on the importance of increasing health data availability to patients. - The study provides recommendations for developing a digital tool that offers personalized and tailored health information to support self-management, ensuring	Health information needs and preferences of IBD patients and HCPs for digital health services; Prioritization of digital health information to support self-management	How can digital health information be prioritised and focussed to support everyday self-management activities among patients with IBD?	- Mixed methods approach has limited consensus on quality standards - Insufficient results for predicting patient needs - Skewed marginals affecting reliability - Limited transferability due to differences in healthcare funding systems - Data not publicly available due to privacy concerns - Need for additional studies to understand health information needs outside clinical settings	2. Self-management behaviours

				hospital in Norway.		accessibility, convenience, and usability without increasing healthcare professionals' workload.				
Development and Validation of an Educational Book on Self-Management in Inflammatory Bowel Disease Based on Patient Preferences and Expert Opinions: A Methodological Study	Narges Norouzkhani [83]	- Describe the development and evaluation process of educational material for self-management in IBD based on patient preferences and expert opinions. - Improve awareness of IBD patients by creating educational material based on patient preferences and expert opinions. - Validate the educational material using PEMAT, SAM, and patient perspectives. - Address the gap in practical, understandable, and appropriate self-management	- Two main phases: development and validation - Comprehensive literature review - Expert opinions for validation - Target audience feedback for validation - Stratified by disease type (UC and CD)	- Two main phases: development and validation - Development phase: - Identification of information needs - Content development through literature review - Validation phase: - Measuring face validity with expert opinions - Validation with experts using PEMAT and SAM tools - Validation with target audiences - Evaluation of understandability and actionability - Calculation of content validity index using SAM - High reliability indicated by Cronbach's	Educational material for self-management in IBD, consisting of 12 chapters, presented to 30 IBD patients (15 UC, 15 CD) for evaluation and use.	- The study developed and validated an educational book on self-management for IBD based on patient preferences and expert opinions. - The educational material was evaluated by a multidisciplinary expert panel and target audiences, with high suitability scores and satisfaction rates. - The study used three evaluation methods: PEMAT, SAM, and patient perspectives, indicating a comprehensive	Suitability score (79.5%), Patient satisfaction (80%), Understandability and actionability (measured by PEMAT and SAM)	How can we develop effective self-management educational resources for inflammatory bowel disease (IBD) that meet patient preferences and are validated by expert opinions?	- The book was written in Persian and only tested on Iranian patients without cross-cultural validation. - The effectiveness of the self-management book in improving knowledge or changing behaviors was not evaluated. - The book needs to be localized and adapted to different languages and cultures. - The best method for delivering educational material is unknown, with potential for electronic materials, games, or video clips.	2. Self-management behaviours

		education for IBD patients.		alpha and ICC coefficients		approach to validation.				
Faecal incontinence intervention study (FINS): self-management booklet information with or without nurse support to improve continence in people with inflammatory bowel disease: study protocol for a randomized controlled trial	Christine Norton [45]	- Determine if implementation of the UK NICE-recommended approach improves bowel control and quality of life in people with IBD. - Determine the effectiveness of using IBD nurses to deliver the algorithm of care compared to a self-management booklet. - Obtain detailed qualitative feedback from patients and staff on health-seeking, intervention experience, and suggestions for future service development.	- Randomized - Controlled - Parallel design - Multi-site - Stratified - Not double-blind (participants and IBD nurses not blinded, but trial statistician blinded) - Not placebo-controlled - Not retrospective - Not observational study - Not meta-analysis - Not systematic review	- Randomized controlled trial with two arms: booklet only vs. booklet plus nurse support. - Participants randomized with 1:1 allocation ratio. - Primary outcome: St Mark's incontinence score at 6 months. - Analysis: Intention-to-treat basis. - Qualitative interviews for participant and healthcare professional views. - Data collection: Self-completed questionnaires. - Study duration: 30 months.	- Self-management booklet: provided to all participants - Sessions with an IBD specialist nurse: 3-4 face-to-face sessions over 3 months, each session lasting 30 minutes (only for Group 1 participants)	- The study evaluates the effectiveness of a self-management booklet with or without nurse support in improving bowel control and quality of life for individuals with inflammatory bowel disease. - The primary outcome measure is the St Mark's incontinence score at 6 months. - The study aims to determine if additional nurse support is crucial for implementing the self-management advice.	St Mark's incontinence score (0-24 scale)	Does implementation of the UK nationally recommended guidance approach to stepwise management of faecal incontinence improve bowel control and quality of life in people with inflammatory bowel disease?	- Blinding to the behavioral intervention is not possible. - The study is not powered to detect significant differences in secondary outcome measures. - Lack of high-quality studies on conservative interventions in inflammatory bowel disease. - Dependence on nurse support for intervention effectiveness.	2. Self-management behaviours
Effects of a Pre-Conception Care Program in Women with Inflammatory Bowel Disease:	Lee, Young [38]	- Evaluate the effectiveness of a pre-conception care program for women with IBD in	- Convergent mixed-methods study design - Randomized	- Mixed-methods study design combining quantitative and qualitative approaches. -	- Pre-conception care program (PCCP-IBD) consisting of four sessions. - Methods: small-	- The pre-conception care program significantly improved self-efficacy for IBD	self-efficacy for IBD management, IBD-related pregnancy knowledge,	What are the effects of a pre-conception care program on self-efficacy for IBD management,	- Participants were recruited from a single hospital, limiting representation of Korean women	2. Self-management behaviours

A Mixed-Methods Study Including a Randomized Controlled Trial		improving self-efficacy for IBD management. - Examine improvements in IBD-related pregnancy knowledge. - Reduce IBD-related pregnancy anxiety.	controlled trial - Single-blind - Randomized controlled pre-posttest design for quantitative data - Focus group interviews and tele-coaching for qualitative data	Randomized controlled trial with intervention and control groups. - Intervention group participated in small-group sessions and individual tele-coaching. - Program consisted of four sessions focusing on IBD management and pre-conception care. - Use of a pre-conception care diary for participant management. - Quantitative data analysis using IBM SPSS Statistics. - Qualitative data analysis using deductive content analysis. - Expert validation and preliminary testing of the program.	group lectures, discussions, video clips, and individual tele-coaching. - Content: IBD management, pre-conception management, contraception, pregnancy management, delivery methods, breastfeeding, and IBD patients' pregnancy experiences. - Participants received a pre-conception care diary for health management and pregnancy preparation. - Duration: Four sessions with data collection before and at one and four weeks post-intervention.	management, IBD-related pregnancy knowledge, and reduced IBD-related pregnancy anxiety in women with IBD. - Qualitative analysis showed improvements in confidence in IBD management, awareness about IBD and pregnancy, and reduced anxiety about pregnancy. - The study is the first to develop and confirm the effectiveness of a pre-conception care program tailored for women with IBD.	IBD-related pregnancy anxiety	IBD-related pregnancy knowledge, and IBD-related pregnancy anxiety in women with inflammatory bowel disease?	with IBD. - The study did not examine differences in program effectiveness based on IBD type. - The study included women who did not intend to become pregnant, which may affect generalizability. - The effectiveness was only assessed over a short period (one and four weeks), necessitating longer-term follow-up studies.	
Self-management education for rehabilitation	Reusch, A [82]	- Primary objective: To evaluate whether the new	cluster-randomized, controlled,	- Cluster-randomized controlled trial - Participants	- A group-based psychoeducation al program consisting of eight	- The psychoeducatio nal self-management	Patient-reported IBD-related concerns	Can a group-based psychoeducatio nal program that	- Intervention and control groups were too similar, limiting	2. Self-manageme nt

inpatients suffering from inflammatory bowel disease: a cluster-randomized controlled trial		psychoeducational program is superior to the control group in reducing patient-reported IBD-related concerns. - Secondary objectives: To assess improvements in disease knowledge, coping, self-management skills, fear of progression, anxiety, depression, and quality of life.	prospective study	assigned to intervention or control group - Intervention group received psychoeducational program with medical and psychological self-management skills - Control group received same medical information but general psychosocial information in lecture format - Program consisted of eight modules (five medical, three psychological) - Large, open groups for medical modules; small, closed groups for psychological modules - Data analysis: analysis of covariance, multiple imputation for missing values - Assessments at baseline, end of rehabilitation, 3 months, and 12 months -	modules, each lasting 1.5 hours. - Five modules cover medical information: structure and function of the gastrointestinal tract, causes and course of IBD, diagnostic and treatment options, pharmacotherapy, complications, and childbearing issues. - Three modules cover psychological self-management skills: sharing personal experiences, coping with anxiety, and role-playing self-confident communication.	program did not prove superior to the control group in terms of primary or secondary outcomes. - Both groups showed positive changes over time in many outcomes, indicating some beneficial effects from both interventions. - The study's conservative design, with similar intervention and control groups, may have contributed to the lack of significant between-group effects.	(measured using PS-CEDE)	combines medical information and psychological self-management skills improve patient-reported IBD-related concerns and other psychosocial outcomes in rehabilitation inpatients with inflammatory bowel disease?	between-group effects. - Outcome measures may not have been sensitive to change. - Lack of a validated instrument to assess patients' knowledge. - Only patient-reported outcomes were used, missing physiological data. - Study was monocentric, raising generalizability concerns. - Lack of information on non-participants. - Potential participant bias towards younger, more educated, or healthier patients.	behaviours

				Conducted in accordance with ethical standards						
Structural equation modeling of the impact of disease activity on inflammatory bowel disease control: the mediating roles of self-efficacy and self-management behaviors	Yongli Zhu [6]	- Investigate how IBD activity status influences disease control through direct and indirect pathways. - Explore how disease activity, self-efficacy, and self-management behaviors contribute to disease control. - Quantify direct, indirect, and mediating effects to understand disease control in IBD patients.	Cross-sectional survey; observational study; single-site study	- Cross-sectional survey among 310 IBD patients. - Structural equation modeling (SEM) using AMOS26 software. - Use of specific questionnaires: IBD-Control Questionnaire, Disease Activity Index, Self-Efficacy to Manage Chronic Disease Scale, Inflammatory Bowel Disease Self-Management Behavior Scale. - Statistical analysis with SPSS version 26 and AMOS version 26.	Not mentioned (the paper does not specify any interventions that participants received)	- Disease activity negatively predicts IBD control, with a significant negative correlation (P < 0.01). - Self-efficacy and self-management behaviors are positively correlated with IBD control, with self-efficacy partially mediating the relationship between disease activity and control. - The study supports multifactorial intervention strategies that integrate self-management behavior and self-efficacy to improve disease control.	Disease control score (range: 0-16)	How does disease activity influence disease control in patients with inflammatory bowel disease (IBD), and what roles do self-efficacy and self-management behaviors play in this process?	- CD and UC were combined into a single IBD category without examining subtype differences. - The study did not fully capture the relationship between disease activity and control, missing factors like psychological well-being and social support. - Self-reported data may introduce social desirability bias. - Data was collected at a single time point, not allowing for observation of changes over time. - The sample was geographically restricted to Chongqing, potentially limiting generalizability.	2. Self-management behaviours

Limited Health Literacy Is Associated With Worse Patient-Reported Outcomes in Inflammatory Bowel Disease	Tormey, Lauren K [50]	- Assess the role of health literacy for patients with IBD. - Assess the prevalence and role of limited health literacy in an adult IBD population. - Examine the significance of medication self-efficacy in the relationship between health literacy and health outcomes.	- Observational cohort study - Cross-sectional analysis - Single-site study - Convenience sample - Non-randomized - Non-controlled - Non-double-blind - Non-placebo-controlled	- Prospective enrollment of adults with IBD from the Boston Medical Center. - Use of standardized questionnaires for health literacy, self-efficacy, quality of life, depression, and clinical disease activity. - Cross-sectional analysis of an observational cohort study. - In-person interviews conducted by trained study team members. - Descriptive statistics and univariate analysis to identify trends and covariates. - Use of regression models (simple linear and logistic) to adjust for confounders.	- 5-ASA (some participants) - Immunomodulator (some participants) - Biologic (some participants) - Combination therapy (some participants) - Steroids (some participants, used in the past 12 months)	- Limited health literacy was identified in 40% of patients with inflammatory bowel disease (IBD). - Patients with limited health literacy reported significantly worse overall health and more depressive symptoms. - Adequate health literacy was associated with higher rates of clinical remission in patients with Crohn's disease.	- Subjective health status (1–5 scale: Excellent to Poor) - Health-related quality of life (SIBDQ score) - Depression (PROMIS Short Form score) - Clinical disease activity (HBI for Crohn's disease, SCCAI for ulcerative colitis)	What is the role of health literacy in patients with inflammatory bowel disease (IBD), and how does it affect patient-reported outcomes?	- small sample size - recruitment method bias - exclusion of non-English speakers and those with significant cognitive impairment - measurement tools not validated across literacy levels - need for future studies to confirm findings and explore causal pathways - need for larger cohorts to evaluate mediating and moderating variables	2. Self-management behaviours
A cluster-randomised controlled trial of a patient-centred guidebook for	Anne Kennedy [34]	- Evaluate the impact of a patient-centred guidebook on knowledge, anxiety, and	- Randomized controlled trial - Cluster-randomized	- Randomized controlled trial (RCT) to evaluate the impact of a patient-centered	- Intervention: Provision of a patient-centred guidebook to patients with ulcerative colitis. -	- Patients receiving the guidebook showed significantly better	- Knowledge of ulcerative colitis (measured using a series of 16 factual questions) -	What is the impact of a patient-centred guidebook on knowledge, anxiety, and	- Underestimated intrahospital correlation may have reduced the power of the study to detect	2. Self-management behaviours

patients with ulcerative colitis: effect on knowledge, anxiety and quality of life		quality of life in patients with ulcerative colitis. - Assess the effect of the guidebook on knowledge, quality of life, and anxiety and depression. - Address the paucity of high-quality information available to patients with ulcerative colitis.	design - Multi-site study - Controlled - Non-double-blind - Non-placebo-controlled	guidebook on knowledge, anxiety, and quality of life in patients with ulcerative colitis. - Guidebook developed with patient involvement, focusing on their identified needs. - 240 patients from six hospitals participated: three control sites and three intervention sites where patients received the guidebook. - Outcomes measured at baseline, 1 month, and 9 months using questionnaires. - Anxiety measured using the Hospital Anxiety and Depression Scale (HADS). - Quality of life measured using the Inflammatory Bowel Disease Questionnaire (IBD QoL). - Knowledge	Description: Full-color guidebook with eight chapters, a section for personal details, and guided self-management sections. - Frequency and Duration: Patients were given the guidebook at the start of the study and asked to read it; effects measured at 1 month and 9 months.	knowledge of their ulcerative colitis at 1 month and 9 months compared to the control group. - Anxiety and quality of life scores remained unchanged throughout the study. - The guidebook provided patient-centered information without increasing anxiety, which is essential for patient involvement in chronic disease management.	Anxiety (measured using the Hospital Anxiety and Depression Scale) - Quality of life (measured using the Inflammatory Bowel Disease Questionnaire)	quality of life in patients with ulcerative colitis?	changes in knowledge. - The study did not assess the impact on doctor-patient interactions or usage frequency. - A larger study is needed to evaluate the guidebook's effectiveness in introducing self-management. - Different outcome measures could have provided better insights.	

				tested using 16 factual questions. - Data analysis performed on an intention-to-treat basis using multivariable analyses with Stata. - Logistic regression models used to assess response rates and missing data patterns.						
Inviting Patients with Inflammatory Bowel Disease to Active Involvement in Their Own Care: A Randomized Controlled Trial	Hueppe, Angelika [32]	- To support patients' self-responsibility in planning and initiating adequate health care through self-assessment and proactive information. - To improve health-related quality of life and social participation. - To test whether the activation and information program improves HRQoL and reduces social participation restrictions compared to	Randomized controlled trial, parallel design, intention-to-treat analysis	- Randomized controlled trial among adult patients with Crohn's disease or ulcerative colitis. - Intervention group received automated data analysis and personalized advice; control group received usual care. - Postal questionnaire assessed somatic and psychosocial problems. - Parallel group design with 1:1 randomization. - Intention-to-treat analysis	- Automated data analysis with individualized written advice on health services - Postal self-administered questionnaire assessing somatic and psychosocial problems - Written feedback within two weeks, including problem assessments and coping recommendations - Accompanying brochure summarizing IBD pathways - Encouragement to discuss feedback with	- The intervention group showed small but statistically significant improvements in health-related quality of life and social participation compared to the control group. - The intervention led to a reduction in disability days and fewer outpatient visits, with no increase in disease activity or hospital stays. - The study	- Health-related quality of life (HRQoL) measured by EuroQol visual analog scale (EQ-VAS) - Social participation measured by Index for measuring participation restriction (IMET) - Disability days (number of days unable to engage in usual activities due to IBD)	Can involving patients with inflammatory bowel disease in their own care through self-assessment and proactive information improve their health-related quality of life and social participation?	- Potential for selective reporting bias due to lack of participant blinding and reliance on patient-reported outcomes. - Sample from a single statutory health insurance may not be representative of the German population. - Reliability of ICD coding for participant recruitment. - Challenges in measuring HRQoL due to lack of	2. Self-manageme nt behaviour s

		standard care. - To support patients' individual responsibility in accessing appropriate health care services, especially for psychosocial problems.		comparing changes in health-related quality of life, social participation, and disability days.	healthcare professionals	concluded that the intervention was effective and beneficial, with a number needed to treat of 9.			satisfactory instruments.	
Life changes, self-prevention, knowledge and mental health among inflammatory bowel disease patients during COVID-19 pandemic: a cross-sectional study	He Shiwen [74]	- Investigate the occurrence of anxiety and depression in IBD patients during the COVID-19 pandemic. - Analyze the factors associated with mental health. - Assess the knowledge of COVID-19. - Assess public self-prevention measures. - Assess daily life changes. - Assess mental health in IBD patients.	Cross-sectional observational study, single-site, online investigation	- Enrolled patients registered at an IBD center. - Used electronic questionnaires to collect data on demographic information, COVID-19 knowledge, self-prevention measures, life changes, and mental health symptoms. - Utilized the Hospital Anxiety and Depression Scale (HADS) to measure anxiety and depression. - Analyzed data using SPSS software with descriptive statistics and non-parametric tests (Mann-	- Regular oral medicine administration (more than 50% of participants) - Timely periodic infusion of biological agents (more than 50% of participants) - Ensuring the supply of routine treatment and medication - Establishing systemic online IBD self-management programs - Telemedicine	- The main factors affecting IBD patients' mental health during the COVID-19 pandemic were diet changes, waiting time for admission, regular oral medicine administration, and timely periodic infusions of biological agents. - These factors were positively correlated with anxiety and depression, except for timely infusions of biological agents, which were negatively correlated. -	Anxiety (HADS-A scores ≥ 8), Depression (HADS-D scores ≥ 8)	What are the factors associated with anxiety and depression in inflammatory bowel disease patients during the COVID-19 pandemic?	- Cross-sectional study cannot predict future mental health developments - Reliance on self-reports may lead to misinterpretation - Lack of investigation into family support's impact on psychological status during isolation - Limited geographical representation (single hospital in central China) - Small sample size for diet changes	2. Self-management behaviours

				Whitney U, Kruskal-Wallis H). - Employed binary logistic regression to assess the impact of demographic backgrounds, COVID-19 knowledge, public health prevention, and life changes on anxiety and depression.		Ensuring routine treatment and medication supply, and establishing online self-management programs, are key strategies to address mental health issues in IBD patients.				
Identification and Evaluation of Mobile Applications for Self-Management of Diet and Lifestyle for Patients with Inflammatory Bowel Disease	Stephanie Gold [54]	- Evaluate current apps available in the US and Canada based on app quality. - Assess perceived impact on diet and mental health. - Evaluate comprehensiveness to support self-management.	Observational study; systematic search and evaluation of mobile apps using the Mobile App Rating Scale (MARS); descriptive statistics analysis.	- Systematic search of Apple iOS and Google Play app stores for IBD-related apps. - Inclusion criteria: apps targeting diet and lifestyle behaviors for IBD patients, available to the general public. - Exclusion criteria: not specific to IBD, not in English, no diet or lifestyle therapy, not available in US and Canada, no stand-alone self-management. - Evaluation using the Mobile App	Mobile health applications (apps) for self-management of diet and lifestyle for patients with IBD, specifically Lyfe MD and My IBD Care: Crohn's and Colitis, which include features such as behavior tracking, diet programs, physical activity programs, mental health self-management, and evidence-informed behavior-change programs. Lyfe MD provides comprehensive support for diet, physical activity,	- Only six out of 1,512 identified apps met the inclusion criteria for supporting self-management of diet and lifestyle for patients with IBD. - Lyfe MD and My IBD Care: Crohn's and Colitis were the top-rated apps, with Lyfe MD offering comprehensive support for diet, physical activity, and mental health, and My IBD Care excelling in mental health	App quality, perceived impact on diet, perceived impact on mental health, comprehensiveness of relevant app features	What are the quality, perceived impact on diet and mental health, and comprehensiveness of mobile health applications available in the US and Canada for self-management of inflammatory bowel disease?	- Only apps available in English were included. - Only apps available in both Canada and the US were evaluated. - Potential bias due to reviewers' connection with Lyfe MD. - Inherent limitations of the MARS tool in assessing content quality and handling missing features. - MARS tool does not assess actual impact or effectiveness of apps on health behaviors.	2. Self-management behaviours

				Rating Scale (MARS) for quality. - Assessment of eight features for comprehensiveness. - Descriptive statistics analysis. - Inter-rater reliability assessment using ICCs.	and mental health, including yoga, breathing, and mindfulness activities.	support. - These two apps were unique in providing behavior tracking and good-quality information, essential for effective self-management.				
Video-Based Educational Interventions for Patients With Chronic Illnesses: Systematic Review	Nikita Deshpande [51]	- Assess the current literature regarding the efficacy of video-based educational tools for patients in improving process and outcome measures across several chronic illnesses. - Confirm prior published evidence that video-based tools improve patient knowledge in the short term. - Systematically review the literature to determine whether patient-targeted video-based	Systematic review; includes superiority randomized controlled trials (66%), pre-post studies (22%), and noninferiority RCTs (12%); no meta-analysis due to heterogeneous outcomes; bias assessed using Cochrane risk-of-bias tools and ROBINS-I.	- Conducted a systematic review using CINAHL and PubMed databases. - Included intervention studies of video-based self-management patient education for various chronic conditions. - Extracted study characteristics, design, demographics, and results from eligible papers. - Assessed bias using Cochrane risk-of-bias tools and ROBINS-I. - Synthesized data using Stata SE and reported according to	- Video-based educational interventions - Often included additional components: printed materials, in-person counseling, interactive modules - Frequency: Some studies involved a single session, others involved repeated sessions - Duration: Follow-up periods ranged from immediately after the intervention to up to 16 months	- Video-based educational tools are most effective in improving patient knowledge, with significant improvements in 75% of knowledge outcomes. - These tools are less consistent in improving disease severity and health care use outcomes, with only about a third of these outcomes showing improvement. - Repeated exposure to video-based interventions may lead to increased	Disease severity, health behavior, patient knowledge, health care use, quality of life (QOL), symptom outcomes, cost outcomes, patient self-confidence	What is the effectiveness of video-based educational tools in improving knowledge, health behaviors, disease severity, and health care use for patients with major chronic illnesses?	- Heterogeneity of factors reviewed makes drawing specific conclusions difficult and precludes meta-analysis. - Enrollment in studies may not reflect real-world behaviors due to higher health literacy. - Few studies targeted populations with lower health literacy or eHealth literacy. - Underrepresentation of cost measures due to need for longer follow-ups and complex data collection. - Further research needed to study diverse	2. Self-management behaviours

		educational self-management tools for chronic illnesses are effective interventions to improve self-management and health outcomes.		PRISMA checklist. - Included a mix of RCTs and pre-post studies.		success, but this was not statistically significant.			populations and collect data on cost effects over longer periods.	
Designing the Essential Informational Needs of a Smartphone Application for Self-Management of Patients With Inflammatory Bowel Disease	Farkhondeh Asadi [65]	- Identify the informational needs of patients with IBD. - Design the essential informational needs for a smartphone application for the self-management of IBD.	- Applied descriptive study - Conducted in two stages - Literature review - Questionnaire-based survey - Single-site study (conducted in one hospital in Tehran) - Observational study	- Conducted a comprehensive literature review. - Designed and validated a questionnaire with three sections: demographic and clinical information, essential informational needs, and required capabilities of a mobile application. - Administered the questionnaire to 180 patients with IBD (120 UC, 60 CD). - Used a 5-point Likert scale to measure importance. - Calculated Cronbach's alpha coefficient for reliability. -	Not mentioned (the paper is about designing informational needs for a smartphone application and does not mention any specific intervention)	- The study identifies the most important informational needs for IBD patients as knowledge of the disease, medication, educational information, complications, diet and nutrition, and lifestyle habits. - Patients with UC need more information on disease knowledge and complications, while patients with CD need more information on diet and nutrition. - The findings can be used to develop and evaluate self-	Informational needs for IBD patients (domains: Knowledge of the disease, Medication, Educational information, Complications, Diet & Nutrition, Lifestyle habits)	What are the informational needs of patients with Inflammatory Bowel Disease (IBD) for effective self-management?	- Limited to a single hospital in Tehran, restricting generalizability. - Findings do not apply to individuals younger than 18. - Smaller sample size of participants with CD compared to UC.	2. Self-management behaviours

				Analyzed data using descriptive statistics and SPSS software.		management applications for IBD patients based on their informational needs.				
Mobile Phone Apps for Inflammatory Bowel Disease Self-Management: A Systematic Assessment of Content and Tools	Con, De Cruz [57]	- Explore the content and tools of existing IBD apps to identify functionalities that may facilitate patient self-management. - Systematically study the content and functions of commercially available apps for IBD patients in the context of current clinical practice guidelines and discuss their utility in assisting patient self-management.	Systematic review; observational study	- Systematic assessment of IBD apps from Google and Apple app stores. - Pre-defined inclusion and exclusion criteria based on language, target audience, and availability. - Evaluation of functionalities, professional medical involvement, consistency with international guidelines, data entry comprehensiveness, and pricing. - Independent assessment by two assessors. - Detailed evaluation of diary apps and disease information apps against	Not applicable (the paper is a systematic assessment of existing IBD apps and does not involve administering interventions to participants)	- Over half of the assessed IBD apps have diary functionalities, and over a third provide health information, which are key features for patient self-management. - None of the apps offer decision support for self-initiation of medical therapy, indicating a significant gap in functionality. - Only 19% of the apps have professional medical involvement, and they cover only 38% of international consensus statements, highlighting major limitations in	Functionality and content of IBD apps in relation to patient self-management	What functionalities do existing IBD apps offer to facilitate patient self-management, and how do they align with current clinical practice guidelines?	- The assessment method for the apps was not validated. - The review was limited to English-language apps and only considered Android and iOS platforms. - Further clinical testing is required to ensure safety and efficacy. - Future studies should explore the perceived needs of patients and clinicians.	2. Self-management behaviours

				international guidelines.		current IBD apps.				
A Systematic Review of Self-Management Interventions for Inflammatory Bowel Disease	Conley, Samantha [77]	- Describe self-management skills in the interventions. - Describe the effects of the interventions on the health-related outcomes measured.	Systematic review of randomized controlled trials; not double-blind; not placebo-controlled; not multi-site; not retrospective; not stratified; not crossover or parallel design; not observational study or meta-analysis.	- Systematic review using PRISMA guidelines - Search conducted using Medline, CINAHL, Embase, Proquest databases - Search terms: inflammatory bowel disease OR Crohn* disease OR ulcerative colitis AND self-management - Additional limits: age ≥18 years, published in English - Single researcher reviewed titles, abstracts, and full texts - Data extraction organized into tables - Self-management skills categorized using Lorig and Holman's framework - Quality assessment using Cochrane risk of bias tool -	- Personalized self-management plans, education on prednisone use, and open-access clinics (Elkjaer et al., 2010; Kennedy et al., 2004; Robinson et al., 2001) - Project management framework, cognitive behavioral therapy, and social learning theory (Keefer et al., 2012) - Gut-directed self-hypnosis (Keefer et al., 2013) - Patient empowerment through tailored advice (Hueppe et al., 2014)	- The interventions reviewed varied in approaches, theoretical perspectives, self-management skills, and outcomes measured. - Four studies demonstrated positive effects of self-management on disease activity. - Two interventions revealed positive effects on disease-specific health-related quality of life (HRQOL), and one intervention revealed positive effects on generic HRQOL.	Health-related quality of life (HRQOL), disease activity, symptoms (anxiety, depression, bowel symptoms, and systemic symptoms)	What are the self-management skills and their effects on health-related outcomes in adults with inflammatory bowel disease?	- Lack of blinding in outcome assessments - Insufficient statistical power analysis - Limited scope of self-management skills covered - Need for further research to identify active components of self-management - Need for more diverse samples in terms of disease activity - Need for more research on symptom outcomes - Limited geographical scope of trials (mostly in Europe) - Single-author review process - Only English-language articles included - Potential for missing grey literature due to limited database searching	2. Self-management behaviours

				Included only randomized controlled trials with diverse characteristics						

Table S3C Quality of Life and Psychological Well-Being

Title	Author	Study objectives	Study design	Methodology	Intervention	Main findings	Outcome measured	Research question	Limitations	Domain
Ulcerative Colitis Narrative Global Survey Findings: Communication Gaps and Agreements between Patients and Physicians	Rubin, D.T. [35]	- Examine patient and physician perspectives on living with UC - Identify gaps in optimal care - Explore patient-physician interactions - Investigate UC management goals - Investigate resources for improving communication	Observational study, multi-site, survey-based	- Questionnaires were conducted across 10 countries. - Covered aspects of UC including diagnosis, treatment, and impact on patient quality of life. - Included standard demographic information. - Descriptive statistics were calculated. - Surveyed 2100 patients and 1254 physicians from August 2017 to February 2018.	Not mentioned (the paper does not discuss any specific medical interventions or treatments)	- A significant majority of patients (85%) are satisfied with their communication with physicians, particularly regarding symptoms and medication options. - Despite this satisfaction, patients express a desire for more information and support at diagnosis, and many do not feel comfortable discussing emotional concerns. - There is an unmet need for better information, materials, and support to enhance patient-physician communication and patient engagement in UC management.	- Patient satisfaction with communication - Patient-physician interactions - UC management goals - Desire for more information at diagnosis - Comfort in discussing emotional concerns - Desire for longer appointments - Importance and discussion of patient advocacy organizations	What are the gaps in optimal care for ulcerative colitis patients, particularly in terms of patient-physician communication and support?	Not mentioned (the paper does not explicitly list any methodological limitations or suggestions for further research)	3. Quality of life & psychological wellbeing

Notable gaps between patients' and physicians' perspectives on communication and disease management in Japan: multifaceted ad hoc analyses of the global Ulcerative Colitis Narrative Survey for further optimal care	Kenji Watanabe [46]	- To reveal practical issues that can be addressed to improve care for patients with UC in Japan. - To improve outcomes for patients living with UC internationally by identifying common and country-specific barriers to better care and proposing solutions.	Observational study; fact-finding survey; non-controlled; non-randomized; non-blinded; non-retrospective; not a meta-analysis or systematic review	- The study used a survey-based methodology with two related surveys: one for patients and one for physicians. - Surveys were conducted online by the Harris Poll between November 2017 and January 2018. - Patients were recruited from online databases and had to meet specific eligibility criteria (e.g., endoscopy-confirmed diagnosis of UC). - Physicians were recruited from online databases and had to meet specific criteria (e.g., seeing ≥5	Not mentioned (the paper does not discuss any specific medical interventions or treatments)	- The study revealed that most patients with ulcerative colitis (UC) in Japan considered themselves in remission despite ongoing impacts on their daily lives. - Physicians focused more on treatment-related topics than quality of life issues, leading to a mismatch in priorities between patients and physicians. - The study emphasizes the importance of patient-physician communication in addressing patient concerns and fears about their condition.	Not mentioned (the paper does not specify a primary outcome or endpoint)	What are the gaps in communication and understanding between patients with ulcerative colitis and their physicians, and how can these gaps be addressed to improve patient care?	- The survey was conducted online, using a self-reported system for disease severity. - Underrepresentation of female physicians in the survey. - Participating physicians were not the same as those treating the participating patients.	3. Quality of life & psychological wellbeing

				UC patients per month). - Survey responses were analyzed using descriptive statistics.						
Patient perspectives on digital patient reported outcomes in routine care of inflammatory bowel disease	Amalie Søgaard Nielsen [14]	- Explore patients' perspectives on the use and non-use of digital patient reported outcomes. - Understand the mechanisms underpinning patient reluctance to engage with this health technology. - Explore IBD patients' experiences and perspectives on an implemented digital PRO system.	Observational study; Mixed-methods action research; Qualitative interviews; Single-site study; Purposive sampling	- Mixed-methods approach with a focus on qualitative interviews. - Qualitative interviews conducted with 16 patients. - Participants recruited based on survey responses. - Interviews conducted in various settings (hospital, home, phone, public space). - Abductive approach used for data analysis. - ReadHy	Digital Patient Reported Outcomes (PROs) system used as a clinical follow-up, preparation for consultations, detection of symptom exacerbation, and prompting contact with the clinic. The system consists of a 44-question questionnaire addressing intestinal and extra-intestinal symptoms, health-related quality of life, and need for patient-clinician contact.	- Patients with sufficient digital literacy perceived digital patient reported outcomes as a useful replacement for face-to-face consultations. - Concerns about digital PROs included their impact on patient-clinician relationships and symptom detection, which can be mitigated by good relationships and direct telephone contact. - The study highlights the importance of the patient-clinician relationship in implementing	- Patients' perspectives on the use and non-use of digital patient reported outcomes - Self-monitoring and insight into symptoms - Emotional distress - Understanding of health concepts and language - Communication with clinicians - Disease control	What are the perspectives of patients with inflammatory bowel disease on the use and non-use of digital patient reported outcomes, and what are the underlying mechanisms of patient reluctance to engage with this technology?	- Recruitment of only one patient who was not using the digital PRO system. - Lack of participants who had actively declined or dropped out of the digital PRO system. - Use of an a priori framework limits the scope of the analysis.	3. Quality of life & psychological wellbeing

				framework used for analysis, focusing on eHealth literacy and readiness dimensions.	Received by 77% of IBD clinic patients since 2017.	digital PROs and notes that patients trust resources saved can be used to improve treatment.				
Information Needs and Preferences of Recently Diagnosed Patients with Inflammatory Bowel Disease	Kylie I Bernstein [56]	- Assess the information needs and experiences of patients recently diagnosed with IBD. - Evaluate what patients view as important information at diagnosis and for new treatments. - Assess satisfaction with previous information and sources of information. - Evaluate preferences for future	Observational study, multi-site	- Recruited 74 patients with Crohn's disease or ulcerative colitis from gastroenterology practices in Winnipeg, Canada. - Participants completed an information needs survey. - Assessed disease status using the Manitoba IBD Index (MIBDI) and the Inflammatory Bowel Disease Quality of Life index (IBDQ). - Rated importance and amount of information	Not mentioned (the paper does not describe a specific intervention administered to participants)	- The most frequent sources of information for recently diagnosed IBD patients were gastroenterologists and the Internet. - A significant portion of patients were dissatisfied or only moderately satisfied with the information received at diagnosis. - Patients preferred receiving information from medical specialists, and supplementing verbal consultations with written materials or website recommendations could improve	- Patient satisfaction with information received at diagnosis - Perceived importance of information topics - Preferences for information sources - Desire for information (measured by Health Opinion Survey Information subscale) - Desire for participation in self-care (measured by	What are the information needs and experiences of patients recently diagnosed with inflammatory bowel disease (IBD)?	- Difficulty in communicating complex information during typical consultation visits - Poor patient memory for oral information - Inadequacy of written materials - Challenges in communicating medical treatment information due to potential disagreements among experts - Lack of consensus on recommendations by physicians - Need for prospective research with substantial	3. Quality of life & psychological wellbeing

		information. - Cover a broader range of information topics than previous research.		received in various areas. - Assessed preferences for receiving information about new treatments. - Ranked sources of information. - Used the Krantz Health Opinion Survey (HOS) to assess desire for information and participation in self-care.		education and satisfaction.	Behavioral Involvement subscale)		sample sizes - Suggestion for a future trial to assess comprehensive information delivery versus usual care	
Knowledge, Quality of Life, and Use of Complementary and Alternative Medicine and Therapies in Inflammatory Bowel Disease: A Comparison of Chinese	Rupert W L Leong [57]	- Compare IBD-related knowledge, QOL, and use of CAMT between Chinese and Caucasian IBD patients. - Examine the relationship between knowledge	Prospective comparative study, observational, multi-site, non-controlled	- Recruited 162 IBD patients (81 Chinese, 81 Caucasian) from ambulatory clinics. - Used a 21-question IBD knowledge questionnaire and the validated Inflammatory Bowel Disease	- Chinese/herbal medicine: used by 41.0% of Chinese and 28.4% of Caucasian subjects - Acupuncture: used by 10.3% of Chinese and 7.4% of Caucasian subjects - Chiropractics:	- Caucasian IBD patients had a higher knowledge score about their condition compared to Chinese patients. - Quality of life was higher in Chinese patients but not significantly different after adjusting for disease activity. - The use of	composite knowledge score, quality of life (IBDQ), disease activity (CDAI and Colitis Activity Index)	How do Chinese and Caucasian patients with inflammatory bowel disease (IBD) differ in terms of IBD-related knowledge, quality of life, and use of	- Previous studies have used nondiscriminating knowledge scoring systems or unvalidated QOL measurements. - Limited consultation time and undifferentiated treatment approach in	3. Quality of life & psychological wellbeing

and Caucasian Patients		and QOL in these populations. - Explore how culture and disease-related knowledge influence the use of CAMT.		Questionnaire (IBDQ) for QOL assessment. - Diagnoses were based on established international criteria. - Nonparametric statistical methods were used for data analysis. - Questionnaire was self-administered and translated into Chinese.	used by 2.6% of Chinese and 2.5% of Caucasian subjects - Reflexology: used by 0% of Chinese and 6.2% of Caucasian subjects - Aromatherapy: used by 0% of Chinese and 2.5% of Caucasian subjects	complementary and alternative medicines and therapies was similar in both groups.		complementary and alternative medicines and therapies, and how do these differences impact their quality of life?	Hong Kong clinics. - Need for prospective review to determine if improving knowledge affects QOL. - Underlying rationale for CAMT use may differ between Chinese and Caucasian patients and requires further study.	
Unmet Communication and Information Needs for Patients with IBD: Implications for Mobile Health Technology	Khan, Sameer [58]	- Explore the major hurdles of living with IBD - Identify the information needs of patients with Crohn's or Ulcerative Colitis - Determine how application technology can be used	Qualitative study using focus groups with 15 IBD patients; non-controlled, observational study design	- 15 IBD patients participated in two focus groups of 120 minutes each. - Data collection methods included focus groups, surveys, and direct observation of patients looking at HealthPROMISE app	Not mentioned (the study did not involve a specific medical intervention or treatment)	- The main needs of IBD patients center around communication issues related to information needs and social impacts. - There is a significant doctor-patient communication divide, including a lack of goal setting and objectivity in disease control. - Patients often misperceive their	Quality of life (QOL) as measured by the Short IBD Questionnaire (SIBDQ), and perceived versus actual control of IBD	What are the major hurdles and information needs of patients with Inflammatory Bowel Disease (IBD), and how can application technology improve	- Limited focus on specific aspects of IBD patient needs and technology use - Use of only two focus groups - Reliance on patient self-reporting and perceptions - Dependence on physician involvement and feedback - Format for integrating	3. Quality of life & psychological wellbeing

		to improve patient quality of life		screenshots. - Surveys collected demographic information, computer literacy, and quality of life data using the Short IBD Questionnaire (SIBDQ). - Qualitative inductive analysis was used to analyze focus group data.		disease control and quality of life, highlighting a need for better information and communication.		their quality of life?	patient education still being refined - Further research or development needed for certain features	
Knowledge and attitudes towards pregnancy in females with Inflammatory Bowel Disease -an international, multi-centre study	Laube, Robyn [40]	- Primary objective: Assess knowledge about pregnancy in females with IBD per clinic-type and per-geographical region. - Secondary objectives: Evaluate VC rates and assess attitudes	Prospective cross-sectional study, multi-site, observational study	- Prospective cross-sectional study design - Recruitment from dedicated IBD-pregnancy clinics, multidisciplinary IBD clinics, and general IBD clinics - Use of the CCPKnow tool to assess pregnancy knowledge - Assessment of	- Dedicated IBD-pregnancy clinics: Comprehensive care including pre-conception counseling, education on risk reduction, fertility, medication management, and delivery options. - Multidisciplinary IBD clinics: Care from dedicated IBD-	- Dedicated IBD-pregnancy clinics had the highest knowledge levels, followed by multidisciplinary IBD clinics and then general IBD clinics. - Geographical regions showed significant differences in knowledge levels, with Western clinics having higher scores than Asian and Middle	CCPKnow score; Voluntary childlessness (VC) rate	What is the relationship between clinic type, geographical region, and knowledge about pregnancy in females with Inflammatory Bowel Disease, and how does this impact voluntary	- Causality cannot be proven between predictors and outcomes. - Practicality of implementing dedicated obstetricians in all settings is limited. - Unknown whether improving IBD knowledge leads to better pregnancy outcomes. -	3. Quality of life & psychological wellbeing

		towards pregnancy among women with IBD.		patient attitudes using a Likert scale - Statistical analysis using non-parametric tests and regression models - Use of SPSS software for analysis - Informed consent obtained from participants	trained gastroenterologists, IBD nurse consultants, dietitians, and psychologists; verbal, written, and online educational materials. - IBD organization membership: Educational support and programs to improve health literacy.	Eastern clinics. - Factors like dedicated IBD-pregnancy clinics, IBD support organization membership, childbearing after IBD diagnosis, and employment predicted higher knowledge levels. - Voluntary childlessness rates varied significantly across clinic types and geographical regions, with dedicated IBD-pregnancy clinics having the lowest rate. - Better knowledge and access to dedicated clinics were associated with decreased voluntary childlessness.		childlessness?	Future prospective studies are needed to assess impact of interventions.	
Patient education in inflammatory bowel disease; a patient-	Edel Mcdermott [62]	- To assess patients' education needs in IBD to facilitate design of a	- Mixed methods approach - Qualitative component: Focus	- Mixed methodology approach combining qualitative and quantitative	- Focus groups with 12 patients to generate hypotheses. - Quantitative questionnaire	- Patients with IBD are most interested in education on medications, future expectations,	Quality of life (Short Health Scale), Knowledge scale,	What are the education needs and preferences of patients with	- Confined to patients attending hospital services - Anonymous data lacks	3. Quality of life & psychological wellbeing

centred, mixed methodology study		patient education programme. - To examine patients' education needs and preferences using a mixed methods approach. - To explore patient education in IBD.	groups with 12 patients - Quantitative component: Questionnaire disseminated to 327 patients - Multi-site: Conducted across<br><br>3 different centers - Observational study	methods. - Qualitative analysis: Focus groups with 12 IBD patients using a grounded theory approach. - Quantitative analysis: Questionnaire disseminated to 327 IBD patients across three centers. - Statistical analysis performed using SPSS. - Questionnaire reviewed for face validity by experts.	disseminated to 327 patients to assess education preferences and needs.	living with IBD, and diet. - They prefer to receive this information from specialist doctors or nurses, believing it can improve their quality of life. - The internet is not preferred for IBD education due to concerns about credibility and reliability.	Perceived improvement in quality of life, Perceived reduction in stress and worry	inflammatory bowel disease (IBD)?	matched disease activity information - Does not include community populations - Needs international validation for generalizability	
Relationship between fear of progression and quality of life in inflammatory bowel disease: Mediating role of health	Xin Hu [31]	- Explore the relationship between fear of progression and health-related quality of life in inflammatory bowel disease	Cross-sectional study; observational study; convenience sampling; single-site study; followed	- Cross-sectional study design - Convenience sampling method at a tertiary hospital in Shenyang, China - Questionnaires used for data	Not mentioned (the study is observational and does not involve administering an intervention)	- Fear of progression is negatively associated with quality of life and directly predicts it. - Health literacy and self-care independently mediate the relationship between fear of	Fear of progression, Health-related quality of life, Health literacy, Self-care	How does fear of progression affect health-related quality of life in inflammatory bowel disease patients, and what are the	- The study is cross-sectional, which limits the ability to establish causal relationships. - Participants were only recruited from one city in northwestern China, which	3. Quality of life & psychological wellbeing

literacy and self-care		patients. - Investigate the sequential mediating roles of health literacy and self-care in this relationship.	STROBE guidelines	collection: Health Literacy Scale for Chronic Disease, Fear of Progression Questionnaire Short Form, Appraisal of the Self-Care Agency Scale-Revised, Inflammatory Bowel Disease Questionnaire - Data analysis using SPSS 25.0 and PROCESS plug-ins - Statistical methods: one-way ANOVA, independent t-tests, Pearson's correlation coefficients, hierarchical regression analysis - Confirmatory factor analysis for construct validity		progression and quality of life. - There is a significant chain mediating effect of health literacy and self-care in this relationship.		mediating roles of health literacy and self-care in this relationship ?	may not be representative of all IBD patients in China. - Self-reported surveys may introduce potential response bias. - A longitudinal follow-up investigation is suggested for future research to explore correlations between variables.	

Assessment of disease specific knowledge and health-related quality of life among United States military veterans with inflammatory bowel disease	Hou, Jason K [68]	- Evaluate the association between patient disease knowledge of IBD and HRQoL. - Identify patient and disease-related predictors of patient knowledge of IBD. - Quantify disease-related knowledge among United States military veterans with IBD receiving care from the VA health system. - Assess the association between knowledge and HRQoL.	Cross-sectional study, observational study	- Cross-sectional study design - Self-administered questionnaires (CCKNOW and SIBDQ) - Demographic and disease characteristics abstracted from electronic medical records - Linear regression and ANOVA for statistical analysis - Patient recruitment from a specific clinic with defined inclusion criteria - Medical chart review using a standardized data collection form - Statistical analysis performed using Stata	- CCKNOW: A 30-item questionnaire assessing patient knowledge of IBD, completed at the time of clinic visit. - SIBDQ: A 10-question questionnaire assessing HRQoL, completed at the time of clinic visit.	- Caucasian race, younger age at diagnosis, and having a college or post-graduate degree were significantly associated with higher knowledge about IBD. - Patient knowledge of IBD was not correlated with health-related quality of life (HRQoL). - Patients with Crohn's disease had higher CCKNOW scores compared to those with ulcerative colitis and unclassified IBD.	- Patient disease knowledge of IBD (measured by CCKNOW) - Health-related quality of life (HRQoL) (measured by SIBDQ)	What is the association between patient knowledge of inflammatory bowel disease (IBD) and health-related quality of life (HRQoL), and what patient and disease-related factors predict patient knowledge of IBD?	- Level of education was missing in 49% of patients. - Self-administered surveys may be impacted by health literacy. - CCKNOW questions were too difficult for half of the patients. - Predominantly male population limits generalizability regarding gender. - Further studies are needed to explore effects on other clinical outcomes.	3. Quality of life & psychological wellbeing

				version 11 software						
Providing Disease-Related Information Worsens Health-Related Quality of Life in Inflammatory Bowel Disease	Mark R Borgaonkar [66]	To assess the effect of an educational intervention on health-related quality of life (HRQOL) in patients with IBD.	- Randomized - Controlled - Single-blind (physicians blinded) - Non-placebo-controlled - Single-site - Prospective - Non-stratified - Parallel design	- Consecutive ambulatory IBD patients were randomized into two groups: one receiving four IBD-specific educational booklets and the other receiving usual care. - Patients completed HRQOL questionnaires (IBDQ and QuICC) at baseline and after 2 weeks. - The educational intervention covered topics such as IBD symptoms, treatments, and lifestyle issues. - Baseline data included demographic	- Intervention: Four IBD-specific educational booklets - Topics covered: General information about IBD, medications, surgery, sexuality, fertility, and pregnancy - Duration: Within the next 2 weeks - Frequency: Not explicitly mentioned - Amount: Four booklets - Participants: 34 patients in the education group	- The addition of educational booklets to IBD patients did not improve and may have worsened short-term HRQOL. - Mean IBDQ scores significantly decreased in the education group compared to controls, indicating a worsening of HRQOL. - Patients without prior educational material showed a trend towards improvement in HRQOL.	Mean change in HRQOL scores (IBDQ and QuICC) between enrollment and 2 weeks after receiving the educational booklets	What is the effect of providing educational materials on health-related quality of life in patients with inflammatory bowel disease?	- The intervention was too simple. - Subjects may not have read the brochures. - Isolation of providing information without discussion with healthcare providers. - Contamination of control group due to external information sources. - No formal assessment of knowledge transfer. - Incomplete responses to questions about information usefulness. - Need for further studies to assess impact on disease activity and HRQOL. - Need to stratify patients by	3. Quality of life & psychological wellbeing

				information and disease activity indices. - Follow-up data was collected via postal return of questionnaires. - Statistical analysis used chi-squared tests for categorical data and Student t tests or Mann-Whitney U tests for continuous data.					disease duration or prior education. - Need for interaction with healthcare professionals to address questions and fears.	
Physical Activity and IBD: State of Art and Knowledge, Patients and Healthcare Professionals Points of View, A French Multicenter Cross	Lea Derbey [75]	- Primary objectives: - Characterize PA habits of IBD patients - Explore limitations of PA - Identify patient knowledge on PA - Secondary objective: -	- Observational study - Cross-sectional study - Multi-site study - Retrospective component	- Conducted a French cross-sectional study from November 2020 to January 2022. - Recruited 298 IBD patients from 4 French hospitals. - Used the International Physical	Not mentioned (the study did not implement any specific interventions)	- Inflammatory bowel disease (IBD) significantly impacts physical activity levels, with a decrease in training frequency and a shift towards lower intensity activities. - Higher physical activity intensity is correlated with better quality of	Physical activity (PA) intensity (measured by IPAQ-SF), Quality of life (QoL) (measured by IBDQ-32)	How does inflammatory bowel disease (IBD) impact physical activity and quality of life in patients, and what are the challenges and limitations	- Retrospective nature of the data prior to IBD diagnosis, leading to possible information bias due to patients' recall of physical activity levels. - Lack of validation of patient and	3. Quality of life & psychological wellbeing

Sectional Study		Explore correlation between PA and QoL - Additional objective: - Assess healthcare professionals' knowledge to improve IBD patient management		Activity Questionnaire short form (IPAQ-SF) and the Inflammatory Bowel Disease Quality of Life Questionnaire (IBDQ). - Patients completed written questionnaires on sociodemographic data and physical activity. - Statistical analysis included χ 2 tests, Student t tests, and ANOVA tests. - Included healthcare professionals to assess their knowledge and practices.		life, regardless of symptom intensity. - Patients face barriers such as fatigue and fear of diarrhea, and feel insufficiently informed about physical activity, while healthcare professionals lack knowledge and support.		faced by these patients in terms of physical activity?	caregiver questionnaires.	
The specialized educational and psychologica	Otilia Gavrilescu [40]	- Evaluate whether cognitive behavioral therapy, in	Randomized controlled trial, parallel	- Randomized controlled trial with 60 IBD patients. - Experimental	- Educational counseling: 1 session per month for 6 months -	- Specialized educational and psychological counseling (SEPC) did not improve	Disease activity (measured by fecal calprotectin	How does cognitive behavioral therapy, in combination	- small group of patients involved - short follow-up period - need for future	3. Quality of life & psychologi

l counseling in inflammatory bowel disease patients -a target or a challenge?		combination with educational counseling, influences disease activity among patients with IBD. - Evaluate whether cognitive behavioral therapy, in combination with educational counseling, influences quality of life (QoL) among patients with IBD.	design, controlled, non-double-blind, non-placebo-controlled, non-multi-site, non-retrospective, non-stratified, non-crossover design, non-observational study, non-meta-analysis, non-systematic review	group received specialized educational and psychological counseling (SEPC). - Control group followed standard medical practice. - IBDQ-32 and BFI questionnaires were used to assess quality of life and personality traits. - Disease activity was measured using fecal calprotectin levels. - Educational counseling was provided monthly for 6 months. - Psychological counseling was based on cognitive behavioral therapy (CBT) techniques,	Psychological counseling: Weekly sessions for 6 months, 2 hours per session, based on cognitive behavioral techniques	disease activity as measured by fecal calprotectin levels. - SEPC significantly improved the quality of life (QoL) of patients, particularly in terms of emotional and social functions. - The improvement in QoL was most pronounced among patients with personality traits of openness to experience or neuroticism.	levels), Quality of life (measured by IBDQ-32)	with educational counseling, influence disease activity and quality of life among patients with inflammatory bowel disease?	studies to assess long-term effects	cal wellbeing

				conducted weekly for 6 months. - Follow-up assessment after 12 months.						
Health Literacy and Quality of Life in Young Adults From The Belgian Crohn's Disease Registry Compared to Type 1 Diabetes Mellitus	Constance Carels [67]	- Determine health literacy (HL), quality of life (QoL), and clinical outcomes in young adults with Crohn's disease compared to type 1 diabetes mellitus. - Assess HL, QoL, work productivity impairment (WPI), and activity impairment (AI) in young Belgian adults with Crohn's disease compared to	Prospective, observational, comparative, multi-site	- Prospective and observational study design - Use of validated questionnaires: HLS-EU-Q16 for health literacy, EQ-5D-5L for quality of life, WPAI for work productivity and activity impairment - Non-parametric statistical analyses (Mann-Whitney U) for group comparisons - Spearman correlations for assessing relationships	Not mentioned (the study is observational and does not involve specific interventions or treatments)	- Young Belgian adults with Crohn's Disease have similar health literacy to those with Type 1 Diabetes Mellitus, but those requiring hospitalization or surgery have lower health literacy. - Health literacy is positively correlated with quality of life in both Crohn's Disease and Diabetes Mellitus patients. - Crohn's Disease patients with active disease or recent hospitalization/surgery have lower health literacy and quality of life	Health Literacy (HL), Quality of Life (QoL), Work Productivity Impairment (WPI), Activity Impairment (AI)	What are the levels of health literacy and quality of life in young Belgian adults with Crohn's disease compared to those with type 1 diabetes mellitus, and how do these factors relate to disease activity and clinical outcomes?	- CD patients came from different centers across Belgium while DM patients were from one center in Brussels. - High dropout rate among BELCRO patients. - Potential bias due to higher education levels among participants. - Different markers for disease activity used for CD and DM patients. - Exclusion of psychological distress as a factor. - Lack of subanalyses based on disease characteristics	3. Quality of life & psychological wellbeing

		young diabetics. - Investigate the relationship between HL, QoL, WPI, and AI with patient characteristics and clinical outcomes, including hospitalizations and surgery in CD patients.		between variables - Institutional review board approval and informed consent from participants - Inclusion of two populations: CD patients from BELCRO registry and type 1 DM patients as a control group - Use of specific language versions of questionnaires (Dutch and French) - Data collection and analysis using Prism version 8 software		compared to those without.			and treatments. - Small sample size affecting statistical reliability. - Potential for self-report bias due to self-assessment tests.	

Table S3D Clinical and healthcare outcomes

Title	Author	Study objectives	Study design	Methodology	Intervention	Main findings	Outcome measured	Research question	Limitations	Domain
IBD LIVE Research sponsored by the Crohn's & Colitis Foundation. Social Media Use and Preferences in Patients With Inflammatory Bowel Disease	Reich, Jason [10]	- Assess social media usage in a large sample of patients with inflammatory bowel disease (IBD). - Assess patient preferences and barriers to social media use in a large internet cohort of IBD patients to target future educational interventions.	Cross-sectional study; observational study; internet-based cohort	- Cross-sectional study within the Crohn's and Colitis Foundation's IBD Partners' internet-based cohort. - Bivariate analyses to compare patient characteristics and social media utilization. - Logistic regression models to determine independent factors associated with social media use for IBD-related information. - 31-question survey to assess patterns and barriers to social media use. - Statistical	Not applicable (the study is observational and does not involve any intervention)	- A large sample of 1960 IBD patients showed significant interest in using social media for disease management, with most spending 30-60 minutes on social media daily. - Factors independently associated with social media use included female gender, younger age, active disease status, and a diagnosis of Crohn's disease. - Concerns about privacy and trust of online information were prevalent, with many patients unsure about the quality of IBD information posted online.	- Social media usage among patients with IBD - Factors independently associated with using social media to obtain IBD-related information: - Female gender - Age - Remission status - Diagnosis type (Crohn's disease vs. ulcerative colitis)	What are the patterns of social media use, patient preferences, and barriers to use among patients with inflammatory bowel disease (IBD)?	- Cross-sectional design - Recruitment from an internet-based cohort not representative of the broader IBD population - Did not assess the type of IBD information patients are seeking - Demographic bias: participants more likely to be younger and female - Need for further research on quality of IBD information on social media and its	4. Clinical & healthcare outcomes

				analyses performed using STATA version 14.0. - Response rate of 69.3%.					effectiveness in improving patient education and outcomes	
Communicating Information Regarding IBD Remission to Patients: Evidence From a Survey of Adult Patients in the United States	Wood, Dallas [71]	- How do patients currently understand remission, do they think remission is possible, and have they ever discussed remission with their healthcare professionals? - Do patients currently face any barriers in communicating with their healthcare professional, and do they have preferences for how information should be	- Observational study - Web-based survey - Randomized assignment within the survey	- Web-based survey sent to adult patients with IBD in the United States. - Survey informed by online focus groups with 43 adult IBD patients. - Survey sections: demographic information, medical history, understanding of remission, and responses to educational material. - Statistical analysis: descriptive statistics, Chi-square tests, unpaired t-tests. - Educational experiment with random assignment to	- Updated Control Version: Educational material explaining IBD remission, designed to improve upon current practice, exposure for at least 30 seconds. - T2T Version: Educational material explaining IBD remission, designed to improve upon current practice, exposure for at least 30 seconds.	- Most patients believe remission is possible in IBD and define it in terms of symptom reduction or absence. - Patients generally report good communication with healthcare professionals, indicating few barriers to discussing remission. - Improved educational materials did not significantly enhance patients' preparedness to discuss their care, but could help resolve the discrepancy in understanding remission.	- Ease of understanding score (scale: 1-5) - Preparedness score (scale: 1-5) - Willingness score (scale: 1-5)	1. How do patients currently understand remission, do they think remission is possible, and have they ever discussed remission with their healthcare professionals? 2. Do patients currently face any barriers in communicating with their healthcare professional, and do they have preferences for how information should be	- The study used a convenience sample from an opt-in online panel, which may not be representative of the broader IBD population. - Risk of inaccurate responses due to self-reporting. - Over-representation of patients with severe IBD. - Selection bias due to systematic differences in individuals who join opt-in panels. - Reliance on	4. Clinical & healthcare outcomes

		received? - Can existing educational material be improved to help patients understand remission better and make them feel more prepared to discuss remission and treatment goals with their healthcare professional?		different educational material versions.				received? 3. Can existing educational material be improved to help patients understand remission better and make them feel more prepared to discuss remission and treatment goals with their healthcare professional?	hypothetical questions about preparedness and willingness to undergo treatments.	
Social Media Use and Preferences in Patients with Inflammatory Bowel Disease	Jason Reich [10]	- Assess social media usage and preferences in patients with IBD. - Investigate patient patterns of use, barriers to use, and preferences for clinicians, hospitals, and IBD-related	Cross-sectional survey, multi-site, self-administered survey, observational study	- Cross-sectional survey design - Survey administered to 118 patients with IBD at Boston Medical Center and the University of Southern Alabama - 41-question survey piloted in 20 patients - Demographic data collected:	Not applicable (the study involved a survey, not an intervention)	- The CCFA website was the most frequently used IBD-specific social media platform among patients. - High-frequency social media users found social media more useful for managing their IBD compared to low-frequency users. - Patients were interested in using social media for	- Social media usage patterns - Barriers to social media use - Preferences for educational information through social media - Disease severity in relation to social media	What are the patterns of social media use, barriers to use, and preferences for educational content among patients with Inflammatory Bowel Disease (IBD)?	- Self-administered survey design - Limited to English-speaking participants - Reliance on self-reported data - Small sample size - Exclusion of participants under 18 years old -	4. Clinical & healthcare outcomes

		organizations to contribute disease-specific educational information through social media.		age, sex, race, education, disease type, severity, duration - Social media use quantified by hours spent on social networking sites - Likert scale responses dichotomized - Univariate and multivariable logistic regression models used for analysis		education and following their gastroenterologist's accounts, but privacy concerns were a significant barrier.	use - Beliefs about the quality of IBD information online		Need for future research to improve generalizability and include younger participants	
Disease-related knowledge and smoking behavior of patients with Crohn's disease	Li Guanwei [19]	- Investigate the status of patients' knowledge on Crohn's disease in China. - Explore the relationship between patients' knowledge and smoking behavior.	Observational, cross-sectional, web-based	- Web-based questionnaires were used to collect data. - Participants were recruited through email invitations and internet advertising. - Sociodemographic data, disease characteristics, and smoking status were recorded. - Follow-up was	Not mentioned (the paper does not describe any specific interventions administered to participants)	- Disease duration and educational status are independent predictors of patients' knowledge about Crohn's disease. - Low knowledge scores are associated with smoking behavior and smoking relapse, emphasizing the importance of patient education. -	CCKNOW total score, smoking rate, smoking relapse	What is the status of patients' knowledge about Crohn's disease in China, and how does this knowledge relate to smoking behavior?	- The study is web-based, which may lead to difficulties in assessing less-educated patients with limited internet access. - The study is observational and cross-sectional, which means it cannot	4. Clinical & healthcare outcomes

				conducted with current and ex-smokers after 6 months. - Statistical methods included independent t-tests, one-way ANOVA, chi-square tests, Pearson correlation analysis, and multiple linear regression.		Patient education is crucial, especially for those from rural areas or with low family income and educational achievement, due to their more defective disease-related knowledge.			establish causality between disease-related knowledge and smoking behavior.	
Assessment of disease-related knowledge and possible factors associated with the knowledge level among Chilean patients with inflammatory bowel disease	Daniela Simian [43]	- To assess disease-related knowledge among patients with IBD. - To identify the factors that are possibly associated with the knowledge level.	Observational, cross-sectional study; prospective; non-probabilistic consecutive enrollment; single-site; not randomized, double-blind, controlled, or placebo-controlled; not a meta-analysis or systematic	- Observational, cross-sectional study design - Use of a Spanish-translated 24-item Crohn's and Colitis Knowledge score to assess disease-related knowledge - Completion of demographic and clinical questionnaires by patients - Ethical approval and informed	- 5-ASA - Immunosuppressant - Steroids - Biological Therapy - Antibiotics	- The median disease-related knowledge score among Chilean IBD patients was low, with only 29% answering more than 50% of questions correctly. - Lower knowledge was observed in areas related to pregnancy/fertility and surgery/complications. - Factors associated with lower knowledge included being older than 50 years,	Disease-related knowledge (measured by CCKNOW score), Factors associated with the level of knowledge	What is the level of disease-related knowledge among Chilean patients with inflammatory bowel disease, and what factors are associated with this knowledge level?	- The study was conducted in a private tertiary care hospital, which may limit the generalizability of the results to other populations. - The Spanish version of the CCKNOW questionnaire was not officially validated. -	4. Clinical & healthcare outcomes

			review; not retrospective; not stratified, crossover, or parallel in design.	consent from patients - Statistical analysis using chi-square, Mann-Whitney, and Kruskal-Wallis tests		having ulcerative colitis, and having a disease duration of less than 5 years.			The reliability of the questionnaire was tested, but not its validity, sensitivity, or cut-off points. - The study's cross-sectional design means that it cannot assess changes in knowledge over time or the impact of educational programs.	
IBD-INFO Questionnaire: A Multicenter French Up-to-Date Survey of Patient Knowledge in Inflammatory Bowel Disease	Pauline Danion [55]	- Develop an updated self-questionnaire to assess patients' level of knowledge of IBD. - Validate a novel self-administered questionnaire composed of translated CCKNOW and	Prospective multicenter cross-sectional study, observational study, multi-site	- Developed and validated a new self-administered questionnaire (IBD-INFO) by translating existing questionnaires (CCKNOW and CCPKNOW) and adding new questions. - Validated the questionnaire	- Anti-TNFα agents: 85.4% of patients - Immunomodulatory drugs: 77.5% of patients - 5-ASA: 71.4% of patients - Steroids: 65.1% of patients - Other biotherapies: 20.1% of patients - Surgery: 31% of patients	- The study developed and validated the IBD-INFO questionnaire to assess patient knowledge about IBD, finding a median total score of 27/64. - Risk factors for poor knowledge included lack of a university degree, not being a member of a patient	- Total score of the IBD-INFO questionnaire (out of 64) - Scores for Q1 (out of 23) - Scores for Q2 (out of 24) - Scores for Q3 (out of 17)	What is the current level of knowledge about inflammatory bowel disease among patients, and what areas of knowledge are most deficient?	- The length of the IBD-INFO questionnaire is a limitation due to its time-consuming nature. - Further study is needed in nontertiary centers to	4. Clinical & healthcare outcomes

		CCPKNOW and new items. - Evaluate the new tool in a population of in-and outpatients to assess their level of knowledge about IBD and identify areas for improvement in education programs.		using non-IBD volunteers with different knowledge levels. - Used statistical tests like Kruskal-Wallis and Cronbach's α to assess reliability and discriminatory ability. - Administered the validated questionnaire to IBD patients in four French university hospitals. - Conducted statistical analyses using tests like Fisher exact test, Mann-Whitney test, and chi-square test to identify factors associated with low knowledge scores.		association, not receiving anti-TNFα treatment, short duration of IBD, male sex, and age over 38 years. - Areas of least knowledge were vaccination, IBD-related cancers, treatments, and pregnancy.			assess the questionnaire's effectiveness. - The study did not involve patients in systematic therapeutic education programs, limiting analysis. - The study is an initial evaluation, suggesting further research is needed to assess long-term effects.	

Table S3E Predictors barrier determinants

Title	Author	Study objectives	Study design	Methodology	Intervention	Main findings	Outcome measured	Research question	Limitations	Domain
Patients With Crohn's Disease Are Unaware of the Risks That Smoking Has on Their Disease	Ryan, William R A [52]	- To assess awareness among patients with Crohn's disease (CD) of the risks of smoking on their disease. - To determine if patients with CD recognize that smoking has an exacerbating effect on their disease.	Observational study; Self-administered questionnaire; Stratified by patient characteristics; Non-controlled; Non-randomized; Non-double-blind; Non-placebo-controlled; Non-retrospective; Non-meta-analysis; Non-systematic review; Non-crossover; Non-parallel design	- Distributed self-administered questionnaires to 714 CD patients. - Included multiple-choice questions about smoking effects on health and CD. - Collected demographic, medical, and smoking history information. - Assessed smoking habits through detailed questions. - Stratified responses by patient characteristics. - Calculated relative proportions and confidence intervals. - Response rate: 43.6% with 312 patients completing the questionnaire.	Not applicable (the study involved administering a questionnaire to assess awareness, not a therapeutic intervention)	- Patients with Crohn's disease are generally aware of the health risks associated with smoking, such as lung cancer and cardiovascular disease. - There is a significant lack of awareness among these patients about the specific risks of smoking related to Crohn's disease, including its development and recurrence. - The study highlights the need for increased	Awareness of smoking risks related to overall health, lung cancer, lung disease, cardiovascular disease, development of CD, reoperation for CD, quantity of cigarettes smoked, and duration of smoking	What is the level of awareness among patients with Crohn's disease about the risks of smoking on their disease?	- Low response rate (43.6%) leading to potential selection bias - Inability to follow up with non-respondents due to time and staff constraints - Questionnaire distribution during a holiday period affecting response rate - Gender bias in the target population (more women than men) - Ethnicity bias in the target population (mostly white) - Surgical nature of the	5. Predictors & barriers / determinants

						patient education on the effects of smoking on Crohn's disease.			patient cohort potentially biasing responses - Self-administered questionnaire subject to recall bias, lack of patient knowledge, and guilty feelings - Sequence of questions potentially influencing respondents' answers regarding CD	
Web-Based Video Education to Improve Uptake of Influenza Vaccination and Other Preventive Health Recommendations in Adults With Inflammatory Bowel Disease: Randomized	Long, Millie D [60]	- Primary objective: To test the impact of preventive health videos on the receipt of the influenza vaccine. - Secondary objectives: To assess the intention to receive other	Randomized controlled trial, stratified by prior year receipt of the influenza vaccine, parallel design, conducted within a web-based cohort, retrospectiv	- Developed animated videos and text-based messages for preventive health education. - Conducted a randomized controlled trial comparing video and text-based interventions. - Participants were randomly assigned to groups, stratified by prior influenza vaccination. -	- Animated videos: 5 videos, 1-2 minutes long, delivered via IBD Partners survey platform, tailored to individual participants' medical and demographic profiles. - Text-only electronic health	- There was no significant difference in the rate of influenza vaccine receipt between video-based and text-based educational interventions among patients with IBD. - Prior	Receipt of the influenza vaccine	How can we improve the uptake of preventive health measures, specifically influenza vaccination, in adults with inflammatory bowel disease?	- Ceiling effect due to high baseline vaccination rate - Lack of racial diversity among participants - High education level among participants - Study not powered for subgroup	5. Predictors & barriers / determinants

Controlled Trial of Project PREVENT		preventive health services and the actual receipt of these services.	ely registered	Primary outcome: patient-reported receipt of influenza vaccine. - Secondary outcomes: intention to receive other preventive services. - Used descriptive statistics and tests of significance for analysis. - Conducted stratified analyses for intervention-effect heterogeneity. - Used logistic regression to adjust for confounding variables.	recommendations: delivered via IBD Partners survey platform, tailored to individual participants' medical and demographic profiles.	receipt of the influenza vaccine was the strongest predictor of both intention to receive and actual receipt of the vaccine. - Older age was associated with a higher likelihood of both intention to receive and actual receipt of the influenza vaccine.			analyses or other preventive health measures - Interventions may not change knowledge or attitudes - Need for future research on patients without prior vaccinations - Further investigation needed for optimal educational methods	
Understanding Health Literacy and its Impact on Delivering Care to Patients with Inflammatory Bowel Disease	Tormey, Lauren K [62]	- Address the impact of health literacy on health outcomes for people with chronic disease, specifically in IBD. - Highlight issues related to	Observational study; cohort study; retrospective analysis; clinical trials mentioned but not specified as part of the	Not mentioned (the paper does not describe a specific methodology for a new study conducted by the authors)	Not mentioned (the paper discusses the need for interventions but does not specify any particular intervention tested in the study)	- Limited health literacy is a significant risk factor for poor health outcomes and communication barriers in chronic diseases, including IBD. - Limited HL affects patients'	Health outcomes in patients with inflammatory bowel disease (IBD), including understanding of screening tests, chronic	How does limited health literacy impact health outcomes in patients with inflammatory bowel disease, and what are effective	- Lack of training, education, and support for healthcare providers to address limited health literacy - Need for more research on the impact of limited health literacy	5. Predictors & barriers / determinants

		patient education and shared decision making in IBD. - Identify communication strategies for clinicians to address these concerns. - Emphasize the need for more research to understand the impact of limited health literacy on IBD health outcomes and to develop effective interventions.	study design			understanding of screening tests, chronic disease management, and their ability to engage in shared decision-making. - Understanding and addressing limited HL is crucial for improving patient-provider communication and implementing effective patient education in IBD.	disease management, and shared decision making	strategies to address these impacts?	on health outcomes in IBD - Need for further studies on the role of health literacy in cancer screening and treatment decisions for IBD patients - Limited formal health literacy education in residency training programs - Lack of confidence among trainees in addressing limited health literacy - Resource limitations and lack of reimbursement for patient education programs	
Internet and Electronic Resources for	Kyle J Fortinsky [47]	- Address important issues of	Systematic review	- Reviewed medical literature on online information quality	- Web-based educational program (no	- The paper reviews the quality and	Quality of online information	What are the challenges and	- Presence of substandard websites -	5. Predictors & barriers

Inflammatory Bowel Disease: A Primer for Providers and Patients		online IBD-related health information and social media activity, such as quality, reliability, objectivity, and privacy. - Review the medical literature on the quality of online information provided to IBD patients. - Summarize the most commonly accessed Websites related to IBD. - Assess the activity on popular social media sites (such as Facebook, Twitter, and YouTube). - Evaluate currently available		for IBD patients. - Searched top three search engines (Google, Yahoo, Bing) using a specific strategy to identify commonly accessed websites. - Assessed activity on social media platforms (Facebook, Twitter, YouTube). - Evaluated mobile applications for IBD patients and providers.	specific frequency or duration mentioned) - myIBD application (no specific frequency or duration mentioned) - Focus Medica Understanding Disease series for IBD ($9.80, Android) - IBD application (text educational material adapted from the CCFA Website)	reliability of online information for IBD patients, highlighting the variability in quality and the lack of correlation between search engine rankings and quality. - It discusses the challenges of navigating online resources due to misinformation and unregulated advertising. - The paper also explores the role of social media in patient empowerment and its potential impact on medical research policy.	, Readability, Integrity (including disclosure of author information and conflicts of interest)	opportunities in using online resources for Inflammatory Bowel Disease (IBD) information, and how can healthcare providers guide patients to reliable online resources?	Uncertainty about search engine algorithm changes - Proprietary nature of search engine algorithms - Lack of regulation on advertisements - Unclear impact of promoting reliable sites - Lack of involvement from healthcare providers in mobile applications - Lack of evidence on application effectiveness - Rapid obsolescence of the paper's content - Need for further research on reliable information, privacy,	/ determinants

		applications for use by IBD patients and providers on mobile phones and tablets. - Develop a list of recommended online resources to strengthen patient participation in their care by providing reliable, comprehensive educational material.							healthcare provider engagement, and impact of online resources	
Inadequate food literacy is related to the worst health status and limitations in daily life in subjects with inflammatory bowel disease	Fiorindi, Camilla [18]	- To measure the degree of food literacy in IBD patients. - To analyze the correlation between food literacy and health and socio-demographi	Observational prospective study	- Observational prospective study involving 450 IBD subjects from A.M.I.C.I. ONLUS association. - Measured food literacy using FLS-IT questionnaire, including NVS for label interpretation. - Analyzed	Not mentioned (the study is observational and does not involve any specific intervention)	- A significant majority (69.78%) of IBD patients had inadequate food literacy, which is associated with poorer health status and more	Degree of Food Literacy (FL) in IBD patients, measured by FLS-IT questionnaire and NVS test; Correlation with health	What is the level of food literacy in patients with Inflammatory Bowel Disease (IBD), and how does it correlate with their	- Online administration during COVID-19 may have limited older patients' participation. - Lengthy questionnaire led to significant	5. Predictors & barriers / determinants

		c variables. - To identify factors related to inadequate food literacy to guide future nutritional strategies.		relationships between food literacy and health/sociodemographic variables using Mann Whitney test, chi-square test, and Spearman's rank correlation. - Used stepwise linear regression to identify predictors of good food literacy skills. - Data analysis performed using GraphPad Prism 7 and Statgraphics Centurion XVI.		limitations in daily life. - Higher food literacy levels were correlated with better health conditions, less daily activity limitations, higher physical activity, and less alcohol consumption. - Women and younger subjects tended to have higher NVS scores, indicating better ability to interpret food labels.	and socio-demographic variables (subjective health conditions, limitations in daily living activities, physical activity, alcohol consumption)	health status and socio-demographic variables?	dropouts, reducing sample size. - Study is limited to Italian patients, restricting generalizability. - Further research is needed to extend findings to other cohorts and develop new guidelines.	
Social Media Use for Inflammatory Bowel Disease in a Rural Appalachian Population	Chowdhary, Tarika Sejal [81]	- Evaluate social media use among patients with IBD in West Virginia. - Examine how social	Observational study	- Survey-based study using REDCap for data collection. - 17-question survey sent to 2,131 patients with IBD in West Virginia. - Survey active for 4 weeks with weekly reminders. -	Not mentioned (the study involved a survey and incentives, not a specific intervention	- Approximately 30% of patients used Facebook for IBD-related information, while 4.3% used	- Use of social media for IBD-related information - Reliability of information obtained	How do patients with Inflammatory Bowel Disease in rural Appalachian areas use	- Exclusion of patients under 18 and over 65 - Perceived reliability influenced by education level and	5. Predictors & barriers / determinants

		media affects patients with IBD in rural Appalachian areas. - Investigate multiple aspects of patients, including demographics and social media use.		Incentivized with a chance to win a $50 gift card. - Data analysis included contingency tables and statistical tests like chi-squared tests. - Patient information obtained using SlicerDicer in EPIC's Electronic Medical Record. - Inclusion criteria: patients aged 18-65 with IBD diagnosis in West Virginia.	for IBD management)	Instagram. - Most patients believed social media had no impact on disease management, but 30.3% believed it had a positive impact. - Almost 45% of patients wished their physician had a social media account for IBD.	from social media - Preference for information sources (social media vs. physicians) - Impact of social media on disease management - Desire for physicians to have social media accounts	social media to gather information about their disease, and what is the perceived impact and reliability of this information on their disease management?	gender - Limited generalizability due to survey being conducted only in West Virginia - Lack of questions about internet use frequency and its impact on behavior - Failure to guide patients to reliable sources - Potential exclusion of patients with lower health literacy due to electronic survey method	
Low Health Literacy Exists in the Inflammatory Bowel Disease (IBD) Population and Is Disproportionately Prevalent	Isabel C Dos, Santos Marques [17]	- Assess the prevalence of low health literacy in a diverse IBD population. - Identify risk factors for	- Single institution - Observational study - Retrospective design - Cross-sectional design - Non-	- Health literacy assessment using the Newest Vital Sign (NVS) - Collection of demographic and socioeconomic data - Extraction of social determinants of health from zip codes - Review of	Not mentioned (the paper does not specify any interventions implemented in the study)	- Low health literacy is present in 24% of the IBD population, with African Americans having a significantly	prevalence of low health literacy (NVS scores 0-3)	What is the prevalence of low health literacy in a diverse IBD population, and what are the risk factors	- Conducted at a single institution, potentially lacking generalizability - Retrospective and cross-sectional	5. Predictors & barriers / determinants

in Older African Americans		low health literacy.	controlled - Non-randomized - Non-blinded	procedure-level data from electronic medical records - Descriptive statistics and multivariable regression analysis using SAS software		higher prevalence (47.5%) compared to white patients (17.0%). - Older age and African American race are associated with low health literacy. - Older African American patients with IBD are at the highest risk of having low health literacy.		associated with it, particularly in terms of racial/ethnic disparities?	design may introduce selection biases - Small study population may lead to type 2 errors in clinical outcome analysis - Limited generalizability to ulcerative colitis due to focus on Crohn disease - Focus on surgical outcomes, not other IBD-specific medical outcomes - Low survey response rate among patients with low health literacy - Need for future studies to build upon findings	

Development of inflammatory bowel disease patient education and medical information sheets: serving an unmet need	Al-Ani, Aysha [68]	- Develop concise, comprehensive patient information sheets for IBD patients in Australia. - Create written evidence-based information resources for patients. - Address an unmet need for IBD patients and their families in Australia. - Empower and engage patients with their healthcare providers.	Not applicable (the paper describes a development project for patient education materials rather than a research study)	- Formation of a multidisciplinary working group. - Series of online meetings for discussion and drafting. - Use of the Evaluative Linguistic Framework for language balance. - Distribution of topics among experts. - Collaboration via a shared electronic drive. - Final approval by the GESA board.	Development of comprehensive patient education and medical information sheets covering various topics related to inflammatory bowel disease (IBD), including diet, travel, vaccinations, sexual health, mental health, surgery, life with a stoma, and pharmaceutical agents. The resources are available online for clinicians and the public.	- A multidisciplinary working group was formed to develop comprehensive patient information sheets for inflammatory bowel disease (IBD) in Australia. - The resources include information sheets on various topics such as diet, travel, vaccinations, and medications, now available online for clinicians and the public. - The development of these resources aims to empower patients and enhance their engagement	Not mentioned (the paper does not specify any measured outcomes or endpoints)	How can we address the unmet need for comprehensive patient education and information resources for individuals with inflammatory bowel disease in Australia?	- Limitations due to COVID-19 pandemic restrictions on face-to-face interactions	5. Predictors & barriers / determinants

						with healthcare providers, addressing a significant gap in patient education and support.				
Inflammatory Bowel Disease Telemedicine Clinical Trial: Impact of Educational Text Messages on Disease-Specific Knowledge Over 1 Year	Abutaleb, Ameer [36]	- Determine if delivery of educational messages through a mobile telemedicine system for IBD can effectively improve patients' disease-specific knowledge. - Compare disease-specific knowledge of IBD over 1 year in patients receiving either standard care or telemedicine intervention	Randomized controlled trial, multi-site, longitudinal design, parallel design	- The study was a randomized controlled trial (RCT) with three arms: two intervention arms receiving educational text messages either weekly or every other week, and a standard care arm. - Participants were randomly assigned to these arms at a 1:1:1 ratio. - The primary outcome measured was the change in disease-specific knowledge over 1 year, assessed using the Crohn's and Colitis Knowledge (CCKNOW) survey at baseline, 6 months, and 12 months. - The study	- TELE-IBD EOW: 1 educational text message per week for 1 year - TELE-IBD W: 2 educational text messages per week for 1 year - Additional "pragmatic" educational messages sent periodically at the discretion of the principal investigator	- Participants in the telemedicine arms showed greater improvement in disease-specific knowledge compared to standard care, but this difference was not significant after adjusting for confounding variables. - The improvement in knowledge was not additive with greater frequency of text messages. - Participants	Change in CCKNOW score over 1 year (range: 0 to 24)	Can delivery of educational messages through a telemedicine system improve disease-specific knowledge in patients with inflammatory bowel disease?	- Patients came from IBD referral centers, which may not be representative of the general IBD population. - Standard care arm may not reflect typical community care due to multidisciplinary teams. - Incomplete assessment of disease knowledge at baseline and follow-up could introduce bias. - Lower sample size led to differences in	5. Predictors & barriers / determinants

		s. - Hypothesize that educational messages through a telemedicine system would improve patients' knowledge of their disease over time and that higher frequency interactions would augment this improvement.		used a mobile telemedicine system for delivering educational messages, with an educational curriculum based on materials from the Crohn's and Colitis Foundation. - Statistical analysis included one-way ANOVA and Pearson's chi-square to compare covariates, and linear regression to adjust for confounding variables.		with lower baseline knowledge scores showed the greatest improvement.			participant characteristics. - Effectiveness of text messaging may vary depending on setting and multidisciplinary teams. - Speculation about whether the intervention's effect was accurately captured by the knowledge survey. - Need for further research to assess effectiveness in different settings and define meaningful changes in disease knowledge.	

References

1. Saez, A.; Herrero-Fernandez, B.; Gomez-Bris, R.; Sánchez-Martinez, H.; Gonzalez-Granado, J.M. Pathophysiology of Inflammatory Bowel Disease: Innate Immune System. *Int. J. Mol. Sci.* **2023**, *24*, 1526. https://doi.org/10.3390/ijms24021526.
2. Khan, S.; Sebastian, S.A.; Parmar, M.P.; Ghadge, N.; Padda, I.; Keshta, A.S.; Minhaz, N.; Patel, A. Factors Influencing the Quality of Life in Inflammatory Bowel Disease: A Comprehensive Review. *Dis. Mon.* **2024**, *70*, 101672. https://doi.org/10.1016/j.disamonth.2023.101672.
3. Napolitano, D.; Bozzetti, M.; Lo Cascio, A.; De Stefano, G.; Orgiana, N.; Lopetuso, L.R.; D'Onofrio, A.M.; Camardese, G.; Papa, A.; Scaldaferri, F.; et al. Resilience and Self-Care in Patients with Inflammatory Bowel Disease: A Multicentre Cross-Sectional Study in Outpatient Settings. *J. Clin. Med.* **2025**, *14*, 3868. https://doi.org/10.3390/jcm14113868.
4. Iizawa, M.; Hirose, L.; Nunotani, M.; Nakashoji, M.; Tairaka, A.; Fernandez, J. A Systematic Review of Self-Management Interventions for Patients with Inflammatory Bowel Disease. *Inflamm. Intest. Dis.* **2023**, *8*, 1–12. https://doi.org/10.1159/000530021.
5. Jackson, B.D.; Gray, K.; Knowles, S.R.; De Cruz, P. EHealth Technologies in Inflammatory Bowel Disease: A Systematic Review. *J. Crohns Colitis* **2016**, *10*, 1103–1121. https://doi.org/10.1093/ecco-jcc/jjw059.
6. Zhu, Y.; Liu, K.; Jiang, J.; Cheng, X.; Wang, H.; Long, F.; Li, K.; Mu, C.; Cui, L. Structural Equation Modeling of the Impact of Disease Activity on Inflammatory Bowel Disease Control: The Mediating Roles of Self-Efficacy and Self-Management Behaviors. *BMC Gastroenterol.* **2025**, *25*, 30. https://doi.org/10.1186/s12876-025-03623-6.
7. De Jong, M.J.; Van Der Meulen-de Jong, A.E.; Romberg-Camps, M.J.; Becx, M.C.; Maljaars, J.P.; Cilissen, M.; Van Bodegraven, A.A.; Mahmmod, N.; Markus, T.; Hameeteman, W.M.; et al. Telemedicine for Management of Inflammatory Bowel Disease (myIBDcoach): A Pragmatic, Multicentre, Randomised Controlled Trial. *The Lancet* **2017**, *390*, 959–968. https://doi.org/10.1016/S0140-6736(17)31327-2.
8. Bailey, S.C.; Serper, M.; Opsasnick, L.; Persell, S.D.; O'Conor, R.; Curtis, L.M.; Benavente, J.Y.; Wismer, G.; Batio, S.; Eifler, M. Changes in COVID-19 Knowledge, Beliefs, Behaviors, and Preparedness among High-Risk Adults from the Onset to the Acceleration Phase of the US Outbreak. *J. Gen. Intern. Med.* **2020**, *35*, 3285–3292.
9. Maurud, S.; Lunde, L.; Moen, A.; Opheim, R. Mapping Conditional Health Literacy and Digital Health Literacy in Patients with Inflammatory Bowel Disease to Optimise Availability of Digital Health Information: A Cross-Sectional Study. *Scand. J. Gastroenterol.* **2025**, *60*, 536–547. https://doi.org/10.1080/00365521.2025.2497952.
10. Reich, J.; Guo, L.; Groshek, J.; Weinberg, J.; Chen, W.; Martin, C.; Long, M.D.; Farraye, F.A. Social Media Use and Preferences in Patients with Inflammatory Bowel Disease. *Inflamm. Bowel Dis.* **2019**, *25*, 587–591. https://doi.org/10.1093/ibd/izy280.
11. Poureslami, I.; Nimmon, L.; Rootman, I.; Fitzgerald, M.J. Health Literacy and Chronic Disease Management: Drawing from Expert Knowledge to Set an Agenda. *Health Promot. Int.* **2016**, *32*, daw003. https://doi.org/10.1093/heapro/daw003.
12. Nutbeam, D.; Lloyd, J.E. Understanding and Responding to Health Literacy as a Social Determinant of Health. *Annu. Rev. Public Health* **2021**, *42*, 159–173. https://doi.org/10.1146/annurev-publhealth-090419-102529.
13. Weiss, B.D. Health Literacy and Patient Safety: Help Patients Understand. In *Manual for Clinicians*; American Medical Association Foundation: Chicago, IL, USA, 2007.
14. Sørensen, K.; Pelikan, J.M.; Röthlin, F.; Ganahl, K.; Slonska, Z.; Doyle, G.; Fullam, J.; Kondilis, B.; Agrafiotis, D.; Uiters, E. Health Literacy in Europe: Comparative Results of the European Health Literacy Survey (HLS-EU). *Eur. J. Public Health* **2015**, *25*, 1053–1058.
15. Hawkins, R.L.; Zia, M.; Hind, D.; Lobo, A.J. Inequalities in Healthcare Access, Experience and Outcomes in Adults with Inflammatory Bowel Disease: A Scoping Review. *Inflamm. Bowel Dis.* **2024**, *30*, 2486–2499. https://doi.org/10.1093/ibd/izae077.
16. Damas, O.M.; Kuftinec, G.; Khakoo, N.S.; Morillo, D.; Quintero, M.A.; Leavitt, J.; Lopez, J.; Kerman, D.H.; Abreu, M.T.; Deshpande, A.R.; et al. Social Barriers Influence Inflammatory Bowel Disease (IBD) Outcomes and Disproportionally Affect Hispanics and Non-Hispanic Blacks with IBD. *Ther. Adv. Gastroenterol.* **2022**, *15*, 17562848221079162. https://doi.org/10.1177/17562848221079162.
17. Dos Santos Marques, I.C.; Theiss, L.M.; Baker, S.J.; Liwo, A.; Wood, L.N.; Cannon, J.A.; Morris, M.S.; Kennedy, G.D.; Fouad, M.N.; Davis, T.C.; et al. Low Health Literacy Exists in the Inflammatory Bowel Disease (IBD) Population and Is Disproportionately Prevalent in Older African Americans. *Crohns Colitis 360* **2020**, *2*, otaa076. https://doi.org/10.1093/crocol/otaa076.
18. Fiorindi, C.; Coppolino, G.; Leone, S.; Previtali, E.; Cei, G.; Luceri, C.; Ficari, F.; Russo, E.; Giudici, F. Inadequate Food Literacy Is Related to the Worst Health Status and Limitations in Daily Life in Subjects with Inflammatory Bowel Disease. *Clin. Nutr. ESPEN* **2022**, *52*, 151–157. https://doi.org/10.1016/j.clnesp.2022.10.016.

19. Li, G.; Ren, J.; Wang, G.; Hu, Q.; Wu, Q.; Wu, X.; Li, R.; Guo, K.; Li, Y.; Gu, G.; et al. Disease-Related Knowledge and Smoking Behavior of Patients with Crohn's Disease. *Dig. Liver Dis.* **2016**, *48*, 1006–1011. https://doi.org/10.1016/j.dld.2016.05.015.
20. Tae, C.H.; Jung, S.-A.; Moon, H.S.; Seo, J.-A.; Song, H.K.; Moon, C.M.; Kim, S.-E.; Shim, K.-N.; Jung, H.-K. Importance of Patients' Knowledge of Their Prescribed Medication in Improving Treatment Adherence in Inflammatory Bowel Disease. *J. Clin. Gastroenterol.* **2016**, *50*, 157–162. https://doi.org/10.1097/MCG.0000000000000431.
21. Selinger, C.P.; Eaden, J.; Selby, W.; Jones, D.B.; Katelaris, P.; Chapman, G.; McDonald, C.; McLaughlin, J.; Leong, R.W.L.; Lal, S. Patients' Knowledge of Pregnancy-related Issues in Inflammatory Bowel Disease and Validation of a Novel Assessment Tool ('CCPKNOW'). *Aliment. Pharmacol. Ther.* **2012**, *36*, 57–63. https://doi.org/10.1111/j.1365-2036.2012.05130.x.
22. Wang, L.; Fan, R.; Zhang, C.; Hong, L.; Zhang, T.; Wang, Z.; Zhong, J. Patients' Educational Program Could Improve Azathioprine Adherence in Crohn's Disease Maintenance Therapy. *Gastroenterol. Res. Pract.* **2020**, *2020*, 6848293. https://doi.org/10.1155/2020/6848293.
23. Miglioretto, C.; Beck, E.; Lambert, K. What Do People with Inflammatory Bowel Disease Want to Know about Diet? The Dietary Information Needs of People with Inflammatory Bowel Disease and Perceptions of Healthcare Providers. *J. Hum. Nutr. Diet.* **2024**, *37*, 706–716. https://doi.org/10.1111/jhn.13297.
24. Rubin, D.C.; Shaker, A.; Levin, M.S. Chronic Intestinal Inflammation: Inflammatory Bowel Disease and Colitis-Associated Colon Cancer. *Front. Immunol.* **2012**, *3*, 107. https://doi.org/10.3389/fimmu.2012.00107.
25. Page, M.J.; McKenzie, J.E.; Bossuyt, P.M.; Boutron, I.; Hoffmann, T.C.; Mulrow, C.D.; Shamseer, L.; Tetzlaff, J.M.; Akl, E.A.; Brennan, S.E.; et al. The PRISMA 2020 Statement: An Updated Guideline for Reporting Systematic Reviews. *BMJ* **2021**, *372*, n71. https://doi.org/10.1136/bmj.n71.
26. Haddaway, N.R.; Grainger, M.J.; Gray, C.T. Citationchaser: A Tool for Transparent and Efficient Forward and Backward Citation Chasing in Systematic Searching. *Res. Synth. Methods* **2022**, *13*, 533–545. https://doi.org/10.1002/jrsm.1563.
27. Briscoe, S.; Bethel, A.; Rogers, M. Conduct and Reporting of Citation Searching in Cochrane Systematic Reviews: A Cross-sectional Study. *Res. Synth. Methods* **2020**, *11*, 169–180. https://doi.org/10.1002/jrsm.1355.
28. Harrison, R.; Jones, B.; Gardner, P.; Lawton, R. Quality Assessment with Diverse Studies (QuADS): An Appraisal Tool for Methodological and Reporting Quality in Systematic Reviews of Mixed- or Multi-Method Studies. *BMC Health Serv. Res.* **2021**, *21*, 144. https://doi.org/10.1186/s12913-021-06122-y.
29. Al-shaari, H.; J, F.; R, M.; Cj, H. A Systematic Review of Repeatability and Reproducibility Studies of Diffusion Tensor Imaging of Cervical Spinal Cord. *Br. J. Radiol.* **2023**, *96*, 20221019. https://doi.org/10.1259/bjr.20221019.
30. McDermott, E.; Healy, G.; Mullen, G.; Keegan, D.; Byrne, K.; Guerandel, A.; Forry, M.; Moloney, J.; Doherty, G.; Cullen, G.; et al. Patient Education in Inflammatory Bowel Disease: A Patient-Centred, Mixed Methodology Study. *J. Crohns Colitis* **2018**, *12*, 419–424. https://doi.org/10.1093/ecco-jcc/jjx175.
31. Rubin, D.T.; Hart, A.; Panaccione, R.; Armuzzi, A.; Suvanto, U.; Deuring, J.J.; Woolcott, J.; Cappelleri, J.C.; Steinberg, K.; Wingate, L.; et al. Ulcerative Colitis Narrative Global Survey Findings: Communication Gaps and Agreements Between Patients and Physicians. *Inflamm. Bowel Dis.* **2021**, *27*, 1096–1106. https://doi.org/10.1093/ibd/izaa257.
32. Hueppe, A.; Langbrandtner, J.; Raspe, H. Inviting Patients with Inflammatory Bowel Disease to Active Involvement in Their Own Care: A Randomized Controlled Trial. *Inflamm. Bowel Dis.* **2014**, *20*, 1057–1069. https://doi.org/10.1097/MIB.0000000000000044.
33. Cross, R.K.; Finkelstein, J. Feasibility and Acceptance of a Home Telemanagement System in Patients with Inflammatory Bowel Disease: A 6-Month Pilot Study. *Dig. Dis. Sci.* **2007**, *52*, 357–364. https://doi.org/10.1007/s10620-006-9523-4.
34. Kennedy, A.; Robinson, A.; Hann, M.; Thompson, D.; Wilkin, D. the North-west Region Gastrointestinal Research Group A Cluster-Randomised Controlled Trial of a Patient-Centred Guidebook for Patients with Ulcerative Colitis: Effect on Knowledge, Anxiety and Quality of Life: Information for Patients with Ulcerative Colitis. *Health Soc. Care Community* **2003**, *11*, 64–72. https://doi.org/10.1046/j.1365-2524.2003.00399.x.
35. Elkjaer, M.; Shuhaibar, M.; Burisch, J.; Bailey, Y.; Scherfig, H.; Laugesen, B.; Avnstrøm, S.; Langholz, E.; O'Morain, C.; Lynge, E.; et al. E-Health Empowers Patients with Ulcerative Colitis: A Randomised Controlled Trial of the Web-Guided 'Constant-Care' Approach. *Gut* **2010**, *59*, 1652–1661. https://doi.org/10.1136/gut.2010.220160.
36. Abutaleb, A.; Buchwald, A.; Chudy-Onwugaje, K.; Langenberg, P.; Regueiro, M.; Schwartz, D.A.; Tracy, J.K.; Ghazi, L.; Patil, S.A.; Quezada, S.M.; et al. Inflammatory Bowel Disease Telemedicine Clinical Trial: Impact of Educational Text Messages on Disease-Specific Knowledge Over 1 Year. *Inflamm. Bowel Dis.* **2018**, *24*, 2191–2197. https://doi.org/10.1093/ibd/izy149.
37. Hu, X.; Xu, L. Relationship between Fear of Progression and Quality of Life in Inflammatory Bowel Disease: Mediating Role of Health Literacy and Self-care. *J. Adv. Nurs.* **2024**, *80*, 4147–4160. https://doi.org/10.1111/jan.16138.

38. Lee, Y.J.; Kim, H.W.; Kim, Y.H.; Yang, S.-K.; Kim, J.-Y. Effects of a Pre-Conception Care Program in Women with Inflammatory Bowel Disease: A Mixed-Methods Study Including a Randomized Controlled Trial. *J. Korean Acad. Nurs.* **2024**, *54*, 386. https://doi.org/10.4040/jkan.24010.
39. Pittet, V.; Rogler, G.; Mottet, C.; Froehlich, F.; Michetti, P.; De Saussure, P.; Burnand, B.; Vader, J.-P.; The Swiss IBD Cohort Study Group. Patients' Information-Seeking Activity Is Associated with Treatment Compliance in Inflammatory Bowel Disease Patients. *Scand. J. Gastroenterol.* **2014**, *49*, 662–673. https://doi.org/10.3109/00365521.2014.896408.
40. Gavrilescu, O.; Prelipcean, C.C.; Dranga, M.; Soponaru, C.; Mihai, C. The Specialized Educational and Psychological Counseling in Inflammatory Bowel Disease Patients—A Target or a Challenge? *Turk. J. Gastroenterol.* **2020**, *31*, 760–768. https://doi.org/10.5152/tjg.2020.19669.
41. Eaden, J.A.; Abrams, K.; Mayberry, J.F. Does Patient Knowledge Affect the Colorectal Cancer Risk in Ulcerative Colitis? *Postgrad. Med. J.* **2002**, *78*, 615–618. https://doi.org/10.1136/pmj.78.924.615.
42. Park, J.; Yoon, H.; Shin, C.M.; Park, Y.S.; Kim, N.; Lee, D.H. Higher Levels of Disease-Related Knowledge Reduce Medical Acceleration in Patients with Inflammatory Bowel Disease. *PLoS ONE* **2020**, *15*, e0233654. https://doi.org/10.1371/journal.pone.0233654.
43. Simian, D.; Flores, L.; Quera, R.; Kronberg, U.; Ibáñez, P.; Figueroa, C.; Lubascher, J. Assessment of Disease-related Knowledge and Possible Factors Associated with the Knowledge Level among Chilean Patients with Inflammatory Bowel Disease. *J. Clin. Nurs.* **2017**, *26*, 1508–1515. https://doi.org/10.1111/jocn.13436.
44. Atreja, A.; Khan, S.; Rogers, J.D.; Otobo, E.; Patel, N.P.; Ullman, T.; Colombel, J.F.; Moore, S.; Sands, B.E.; Health PROMISE Consortium Group. Impact of the Mobile HealthPROMISE Platform on the Quality of Care and Quality of Life in Patients with Inflammatory Bowel Disease: Study Protocol of a Pragmatic Randomized Controlled Trial. *JMIR Res. Protoc.* **2015**, *4*, e23. https://doi.org/10.2196/resprot.4042.
45. Norton, C.; Dibley, L.B.; Hart, A.; Duncan, J.; Emmanuel, A.; Knowles, C.H.; Stevens, N.; Terry, H.; Verjee, A.; Kerry, S.; et al. Faecal Incontinence Intervention Study (FINS): Self-Management Booklet Information with or without Nurse Support to Improve Continence in People with Inflammatory Bowel Disease: Study Protocol for a Randomized Controlled Trial. *Trials* **2015**, *16*, 444. https://doi.org/10.1186/s13063-015-0962-0.
46. Watanabe, K.; Gardiner, S.; Arai, S. Notable Gaps between Patients' and Physicians' Perspectives on Communication and Disease Management in Japan: Multifaceted Ad Hoc Analyses of the Global Ulcerative Colitis Narrative Survey for Further Optimal Care. *Ther. Adv. Gastroenterol.* **2022**, *15*, 17562848221095372. https://doi.org/10.1177/17562848221095372.
47. Fortinsky, K.J.; Fournier, M.R.; Benchimol, E.I. Internet and Electronic Resources for Inflammatory Bowel Disease: A Primer for Providers and Patients: *Inflamm. Bowel Dis.* **2012**, *18*, 1156–1163. https://doi.org/10.1002/ibd.22834.
48. McMaster, K.; Aguinaldo, L.; Parekh, N.K. Evaluation of an Ongoing Psychoeducational Inflammatory Bowel Disease Support Group in an Adult Outpatient Setting. *Gastroenterol. Nurs.* **2012**, *35*, 383–390. https://doi.org/10.1097/SGA.0b013e3182747940.
49. Napolitano, D.; Vellone, E.; Iovino, P.; Scaldaferri, F.; Cocchieri, A. Self-Care in Patients Affected by Inflammatory Bowel Disease and Caregiver Contribution to Self-Care (IBD-SELF): A Protocol for a Longitudinal Observational Study. *BMJ Open Gastroenterol.* **2024**, *11*, e001510. https://doi.org/10.1136/bmjgast-2024-001510.
50. Tormey, L.K.; Reich, J.; Chen, Y.S.; Singh, A.; Lipkin-Moore, Z.; Yu, A.; Weinberg, J.; Farraye, F.A.; Paasche-Orlow, M.K. Limited Health Literacy Is Associated with Worse Patient-Reported Outcomes in Inflammatory Bowel Disease. *Inflamm. Bowel Dis.* **2019**, *25*, 204–212. https://doi.org/10.1093/ibd/izy237.
51. Deshpande, N.; Wu, M.; Kelly, C.; Woodrick, N.; Werner, D.A.; Volerman, A.; Press, V.G. Video-Based Educational Interventions for Patients with Chronic Illnesses: Systematic Review. *J. Med. Internet Res.* **2023**, *25*, e41092. https://doi.org/10.2196/41092.
52. Ryan, W.R.; Ley, C.; Allan, R.N.; Keighley, M.R.B. Patients with Crohn's Disease Are Unaware of the Risks That Smoking Has on Their Disease. *J. Gastrointest. Surg.* **2003**, *7*, 706–711. https://doi.org/10.1016/S1091-255X(03)00066-0.
53. Xie, H.; Zhang, J.; Liu, C.; Yang, B.; Dong, W. Development and Validation of a Questionnaire to Test Chinese Patients' Knowledge of Inflammatory Bowel Disease. *Sci. Rep.* **2023**, *13*, 7061. https://doi.org/10.1038/s41598-023-34286-6.
54. Gold, S.L.; Chiew, B.A.; Rajagopalan, V.; Lavallee, C.M. Identification and Evaluation of Mobile Applications for Self-Management of Diet and Lifestyle for Patients with Inflammatory Bowel Disease. *J. Can. Assoc. Gastroenterol.* **2023**, *6*, 186–195. https://doi.org/10.1093/jcag/gwad029.
55. Danion, P.; Buisson, A.; Roblin, X.; Mathieu, N.; Charlois, A.-L.; Borgerding, J.N.; Williet, N.; Del Tedesco, E.; Flourié, B.; Nancey, S.; et al. IBD-INFO Questionnaire: A Multicenter French Up-to-Date Survey of Patient Knowledge in Inflammatory Bowel Disease. *Inflamm. Bowel Dis.* **2018**, *24*, 943–952. https://doi.org/10.1093/ibd/izx073.
56. Cross, R.K.; Jambaulikar, G.; Langenberg, P.; Tracy, J.K.; Collins, J.F.; Katz, J.; Regueiro, M.; Schwartz, D.A.; Quinn, C.C. TELEmedicine for Patients with Inflammatory Bowel Disease (TELE-IBD): Design and Implementation of Randomized Clinical Trial. *Contemp. Clin. Trials* **2015**, *42*, 132–144. https://doi.org/10.1016/j.cct.2015.03.006.

57. Con, D.; De Cruz, P. Mobile Phone Apps for Inflammatory Bowel Disease Self-Management: A Systematic Assessment of Content and Tools. *JMIR MHealth UHealth* **2016**, *4*, e13. https://doi.org/10.2196/mhealth.4874.
58. Knowles, S.R.; Alex, G. Medication Adherence Across the Life Span in Inflammatory Bowel Disease: Implications and Recommendations for Nurses and Other Health Providers. *Gastroenterol. Nurs.* **2020**, *43*, 76–88. https://doi.org/10.1097/SGA.0000000000000467.
59. Cross, R.K.; Finkelstein, J. Challenges in the Design of a Home Telemanagement Trial for Patients with Ulcerative Colitis. *Clin. Trials* **2009**, *6*, 649–657. https://doi.org/10.1177/1740774509346978.
60. Long, M.D.; Van Deen, W.K.; Weisbein, L.; Khalil, C.; Appel, K.L.; Zhang, X.; Chen, W.; Zubrod, L.; Maris, R.; Ghafari, A.; et al. Web-Based Video Education to Improve Uptake of Influenza Vaccination and Other Preventive Health Recommendations in Adults with Inflammatory Bowel Disease: Randomized Controlled Trial of Project PREVENT. *J. Med. Internet Res.* **2023**, *25*, e42921. https://doi.org/10.2196/42921.
61. Gordon, M.; Sinopoulou, V.; Ibrahim, U.; Abdulshafea, M.; Bracewell, K.; Akobeng, A.K. Patient Education Interventions for the Management of Inflammatory Bowel Disease. *Cochrane Database Syst. Rev.* **2023**, *2023*, CD013854. https://doi.org/10.1002/14651858.CD013854.pub2.
62. Tormey, L.K.; Farraye, F.A.; PaascheOrlow, M.K. Understanding Health Literacy and Its Impact on Delivering Care to Patients with Inflammatory Bowel Disease: *Inflamm. Bowel Dis.* **2016**, *22*, 745–751. https://doi.org/10.1097/MIB.0000000000000622.
63. Hou, J.K.; Turkeltaub, J.A.; Iii, T.R.M.; El-Serag, H.B. Assessment of Disease Specific Knowledge and Health-Related Quality of Life among United States Military Veterans with Inflammatory Bowel Disease. *World J. Gastroenterol.* **2015**, *21*, 6001–6007. https://doi.org/10.3748/wjg.v21.i19.6001.
64. Ajibulu, L.; Chappell, K.D.; Seow, C.H.; Goodman, K.J.; Wong, K. "Simpleness": A Qualitative Description Study Exploring Patient Perspectives on the Barriers and Facilitators of Using Digital Health Tools to Self-Manage Inflammatory Bowel Disease. *Ther. Adv. Gastroenterol.* **2025**, *18*, 17562848251319807. https://doi.org/10.1177/17562848251319807.
65. Asadi, F.; Hosseini, A.; Daeechini, A.H. Designing the Essential Informational Needs of a Smartphone Application for Self-Management of Patients with Inflammatory Bowel Disease. *Health Sci. Rep.* **2024**, *7*, e70186. https://doi.org/10.1002/hsr2.70186.
66. Borgaonkar, M.R.; Townson, G.; Donnelly, M.; Irvine, E.J. Providing Disease-Related Information Worsens Health-Related Quality of Life in Inflammatory Bowel Disease: *Inflamm. Bowel Dis.* **2002**, *8*, 264–269. https://doi.org/10.1097/00054725-200207000-00005.
67. Carels, C.; Wauters, L.; Outtier, A.; Baert, F.; Bossuyt, P.; Colard, A.; De Looze, D.; Ferrante, M.; Goegebuer, A.; Hauser, B.; et al. Health Literacy and Quality of Life in Young Adults from The Belgian Crohn's Disease Registry Compared to Type 1 Diabetes Mellitus. *Front. Pediatr.* **2021**, *9*, 624416. https://doi.org/10.3389/fped.2021.624416.
68. Al-Ani, A.; GESA IBD Patient Information Materials Working Group; Garg, M. Development of Inflammatory Bowel Disease Patient Education and Medical Information Sheets: Serving an Unmet Need. *Intern. Med. J.* **2022**, *52*, 1272–1275. https://doi.org/10.1111/imj.15840.
69. Laube, R.; Yau, Y.; Selinger, C.P.; Seow, C.H.; Thomas, A.; Wei Chuah, S.; Hilmi, I.; Mao, R.; Ong, D.; Ng, S.C.; et al. Knowledge and Attitudes Towards Pregnancy in Females with Inflammatory Bowel Disease: An International, Multi-Centre Study. *J. Crohns Colitis* **2020**, *14*, 1248–1255. https://doi.org/10.1093/ecco-jcc/jjaa047.
70. Lesnovska, K.P.; Börjeson, S.; Hjortswang, H.; Frisman, G.H. What Do Patients Need to Know? Living with Inflammatory Bowel Disease. *J. Clin. Nurs.* **2014**, *23*, 1718–1725. https://doi.org/10.1111/jocn.12321.
71. Wood, D.W.; Treiman, K.; Rivell, A.; Van Deen, W.K.; Heyison, H.; Mattar, M.C.; Power, S.; Strauss, A.; Syal, G.; Zullow, S.; et al. Communicating Information Regarding IBD Remission to Patients: Evidence from a Survey of Adult Patients in the United States. *Inflamm. Bowel Dis.* **2025**, *31*, 1605–1615. https://doi.org/10.1093/ibd/izae201.
72. Bernstein, K.I.; Promislow, S.; Carr, R.; Rawsthorne, P.; Walker, J.R.; Bernstein, C.N. Information Needs and Preferences of Recently Diagnosed Patients with Inflammatory Bowel Disease: *Inflamm. Bowel Dis.* **2011**, *17*, 590–598. https://doi.org/10.1002/ibd.21363.
73. Khan, S.; Dasrath, F.; Farghaly, S.; Otobo, E.; Riaz, M.; Rogers, J.; Castillo, A.; Atreja, A. Unmet Communication and Information Needs for Patients with IBD: Implications for Mobile Health Technology. *Br. J. Med. Med. Res.* **2016**, *12*, 1–11. https://doi.org/10.9734/BJMMR/2016/21884.
74. He, S.; Xiao, T.; Xia, Y. Life Changes, Self-Prevention, Knowledge and Mental Health among Inflammatory Bowel Disease Patients during COVID-19 Pandemic: A Cross-Sectional Study. *Front. Public Health* **2024**, *12*, 1416880. https://doi.org/10.3389/fpubh.2024.1416880.
75. Derbey, L.; Charlois, A.L.; Buisson, A.; Roblin, X.; Mathieu, N.; Danion, P.; Gay, C.; Nancey, S.; Boschetti, G. Physical Activity and IBD: State of Art and Knowledge, Patients and Healthcare Professionals Points of View, A French Multicenter Cross Sectional Study. *Inflamm. Bowel Dis.* **2024**, *30*, 2306–2313. https://doi.org/10.1093/ibd/izae009.

76. Daher, S.; Khoury, T.; Benson, A.; Walker, J.R.; Hammerman, O.; Kedem, R.; Naftali, T.; Eliakim, R.; Ben-Bassat, O.; Bernstein, C.N.; et al. Inflammatory Bowel Disease Patient Profiles Are Related to Specific Information Needs: A Nationwide Survey. *World J. Gastroenterol.* **2019**, *25*, 4246–4260. https://doi.org/10.3748/wjg.v25.i30.4246.
77. Conley, S.; Redeker, N. A Systematic Review of Self-Management Interventions for Inflammatory Bowel Disease. *J. Nurs. Scholarsh.* **2016**, *48*, 118–127. https://doi.org/10.1111/jnu.12189.
78. Maurud, S.; Lunde, L.; Moen, A.; Opheim, R. Exploring the Foundations of a Digital Health Information Service for Patients with Inflammatory Bowel Disease: A Mixed Method Study in Gravitate-Health. *BMC Gastroenterol.* **2024**, *24*, 184. https://doi.org/10.1186/s12876-024-03272-1.
79. Akbarian, P.; Asadi, F.; Sabahi, A. Developing Mobile Health Applications for Inflammatory Bowel Disease: A Systematic Review of Features and Technologies. *Middle East J. Dig. Dis.* **2024**, *16*, 211–220. https://doi.org/10.34172/mejdd.2024.394.
80. Nielsen, A.S.; Appel, C.W.; Larsen, B.F.; Kayser, L.; Hanna, L. Patient Perspectives on Digital Patient Reported Outcomes in Routine Care of Inflammatory Bowel Disease. *J. Patient-Rep. Outcomes* **2021**, *5*, 92. https://doi.org/10.1186/s41687-021-00366-2.
81. Chowdhary, T.S.; Thompson, J.; Gayam, S. Social Media Use for Inflammatory Bowel Disease in a Rural Appalachian Population. *Telemed. E-Health* **2021**, *27*, 402–408. https://doi.org/10.1089/tmj.2020.0014.
82. Reusch, A.; Weiland, R.; Gerlich, C.; Dreger, K.; Derra, C.; Mainos, D.; Tuschhoff, T.; Berding, A.; Witte, C.; Kaltz, B.; et al. Self-Management Education for Rehabilitation Inpatients Suffering from Inflammatory Bowel Disease: A Cluster-Randomized Controlled Trial. *Health Educ. Res.* **2016**, *31*, cyw042. https://doi.org/10.1093/her/cyw042.
83. Norouzkhani, N.; Bahari, A.; Faramarzi, M.; Shokri Shirvani, J.; Eslami, S.; Tabesh, H. Development and Validation of an Educational Book on Self-Management in Inflammatory Bowel Disease Based on Patient Preferences and Expert Opinions: A Methodological Study. *J. Clin. Med.* **2023**, *12*, 7659. https://doi.org/10.3390/jcm12247659.
84. Matthias, C.; Fawson, S.; Yan, L.; Sweeney, L.; Moss-Morris, R.; Norton, C. Inflammatory Bowel Disease Nurses' Views on Taking on a New Role to Support an Online Self-Management Programme for Symptoms of Fatigue, Pain and Urgency: A Qualitative Study to Maximise Intervention Acceptance. *Gastrointest. Nurs.* **2021**, *19*, 28–35. https://doi.org/10.12968/gasn.2021.19.9.28.
85. Moradkhani, A.; Kerwin, L.; Dudley-Brown, S.; Tabibian, J.H. Disease-Specific Knowledge, Coping, and Adherence in Patients with Inflammatory Bowel Disease. *Dig. Dis. Sci.* **2011**, *56*, 2972–2977. https://doi.org/10.1007/s10620-011-1714-y.
86. Patil, S.A.; Cross, R.K. Current Landscape of Telemedicine Practice in Inflammatory Bowel Disease. *Inflamm. Bowel Dis.* **2018**, *24*, 1910–1917. https://doi.org/10.1093/ibd/izy113.
87. Reich, J.; Guo, L.; Hall, J.; Tran, A.; Weinberg, J.; Groshek, J.; Rowell, T.E.; DiPalma, J.A.; Farraye, F.A. A Survey of Social Media Use and Preferences in Patients with Inflammatory Bowel Disease: *Inflamm. Bowel Dis.* **2016**, *22*, 2678–2687. https://doi.org/10.1097/MIB.0000000000000951.
88. Leong, R.W.L.; Lawrance, I.C.; Ching, J.Y.L.; Cheung, C.M.Y.; Fung, S.S.L.; Ho, J.N.C.; Philpott, J.; Wallace, A.R.; Sung, J.J.Y. Knowledge, Quality of Life, and Use of Complementary and Alternative Medicine and Therapies in Inflammatory Bowel Disease: A Comparison of Chinese and Caucasian Patients. *Dig. Dis. Sci.* **2004**, *49*, 1672–1676. https://doi.org/10.1023/B:DDAS.0000043384.26092.f4.
89. Kaps, L.; Omogbehin, L.; Hildebrand, K.; Gairing, S.J.; Schleicher, E.M.; Moehler, M.; Rahman, F.; Schattenberg, J.M.; Wörns, M.-A.; Galle, P.R.; et al. Health Literacy in Gastrointestinal Diseases: A Comparative Analysis between Patients with Liver Cirrhosis, Inflammatory Bowel Disease and Gastrointestinal Cancer. *Sci. Rep.* **2022**, *12*, 21072. https://doi.org/10.1038/s41598-022-25699-w.
90. Urstad, K.H.; Andersen, M.H.; Larsen, M.H.; Borge, C.R.; Helseth, S.; Wahl, A.K. Definitions and Measurement of Health Literacy in Health and Medicine Research: A Systematic Review. *BMJ Open* **2022**, *12*, e056294. https://doi.org/10.1136/bmjopen-2021-056294.
91. Van Deen, W.K.; Khalil, C.; Dupuy, T.; Bonthala, N.; Almario, C.; Spiegel, B. S0813 The Effect of Educational Videos on Inflammatory Bowel Disease Patients' Engagement and Their Friends' and Family Members' Level of Empathy. *Am. J. Gastroenterol.* **2020**, *115*, S417–S418. https://doi.org/10.14309/01.ajg.0000705300.61083.d8.
92. Bacsur, P.; Ivány, E.; Farkas, B.; Bálint, A.; Fábián, A.; Bor, R.; Rutka, M.; Bősze, Z.; Szepes, Z.; Molnár, T.; et al. P0963 Nationwide Analysis of Targeted Telemedicinal Patient Education in IBD—A Randomized-Controlled Trial. *J. Crohns Colitis* **2025**, *19*, i1793–i1795. https://doi.org/10.1093/ecco-jcc/jjae190.1137.
93. Blunck, D.; Kastner, L.; Nissen, M.; Winkler, J. The Effectiveness of Patient Training in Inflammatory Bowel Disease Knowledge via Instagram: Randomized Controlled Trial. *J. Med. Internet Res.* **2022**, *24*, e36767. https://doi.org/10.2196/36767.
94. Mercuri, C.; Giordano, V.; Bosco, V.; Serra, N.; Spagnuolo, R.; Nocerino, R.; Rea, T.; Colaci, C.; Guillari, A.; Doldo, P.; et al. Impact of Nursing Interventions via Telephone and Email on the Quality of Life of Patients with Inflammatory Bowel Disease: Preliminary Results of a Comparative Observational Study. *Healthcare* **2024**, *12*, 2538. https://doi.org/10.3390/healthcare12242538.
95. Axelrad, J.; Long, M.; Horst, S.; Afzali, A.; Sapir, T.; Fajardo, K.; De Felice, K.; Sandler, R.; Cross, R. A Novel Remote Patient and Medication Monitoring Solution to Improve Adherence and Persistence With Inflammatory Bowel Disease Therapy (ASSIST Study): Protocol for a Randomized Controlled Trial. *JMIR Res. Protoc.* **2022**, *11*, e40382. https://doi.org/10.2196/40382.

96. Hajlaoui, A.; Sabbah, M.; Bibani, N.; Jlassi, H.; Bellil, N.; Lassoued, K.; Trad, D.; Gargouri, D. P403 Therapeutic Compliance in Patients with Chronic Inflammatory Bowel Disease. *J. Crohns Colitis* **2023**, *17*, i533. https://doi.org/10.1093/ecco-jcc/jjac190.0533.
97. Pintér, H.K.; Nagy, V.A.; Csobod, É.C.; Cseh, Á.; Béres, N.J.; Prehoda, B.; Dezsőfi-Gottl, A.; Veres, D.S.; Pálfi, E. Health Literacy and Nutrition of Adolescent Patients with Inflammatory Bowel Disease. *Nutrients* **2025**, *17*, 2458. https://doi.org/10.3390/nu17152458.
98. Yin, T.; Tu, W.; Li, Y.; Yang, M.; Huang, L.; Zhang, S.; Xu, G. Risk of Avoidant/Restrictive Food Intake Disorder in Patients with Inflammatory Bowel Disease: Predictive Value of Disease Phenotype, Disease Activity and Food Literacy. *J. Eat. Disord.* **2023**, *11*, 211. https://doi.org/10.1186/s40337-023-00936-3.
99. Norouzkhani, N.; Faramarzi, M.; Ghodousi Moghadam, S.; Karimi, M.A.; Shokri Shirvani, J.; Bahari, A.; ShojaeiBaghini, M.; Eslami, S.; Tabesh, H. Identification of the Informational and Supportive Needs of Patients Diagnosed with Inflammatory Bowel Disease: A Scoping Review. *Front. Psychol.* **2023**, *14*, 1055449. https://doi.org/10.3389/fpsyg.2023.1055449.
100. Nam, H.J.; Yoon, J.Y. Pathways Linking Health Literacy to Self-Care in Diabetic Patients with Physical Disabilities: A Moderated Mediation Model. *PLoS ONE* **2024**, *19*, e0299971. https://doi.org/10.1371/journal.pone.0299971.
101. Lee, Y.-J.; Shin, S.-J.; Wang, R.-H.; Lin, K.-D.; Lee, Y.-L.; Wang, Y.-H. Pathways of Empowerment Perceptions, Health Literacy, Self-Efficacy, and Self-Care Behaviors to Glycemic Control in Patients with Type 2 Diabetes Mellitus. *Patient Educ. Couns.* **2016**, *99*, 287–294. https://doi.org/10.1016/j.pec.2015.08.021.
102. Berding, A.; Witte, C.; Gottschald, M.; Kaltz, B.; Weiland, R.; Gerlich, C.; Reusch, A.; Kruis, W.; Faller, H. Beneficial Effects of Education on Emotional Distress, Self-Management, and Coping in Patients with Inflammatory Bowel Disease: A Prospective Randomized Controlled Study. *Inflamm. Intest. Dis.* **2016**, *1*, 182–190. https://doi.org/10.1159/000452989.
103. Stichler, J.F.; Pelletier, L.R. Applying the Patient Empowerment, Engagement, and Activation Survey to Improve Patient Outcomes. *JONA J. Nurs. Adm.* **2023**, *53*, 668–674. https://doi.org/10.1097/NNA.0000000000001364.
104. Muñoz-Villaverde, S.; Martínez-García, M.; Serrano-Oviedo, L.; Gómez-Romero, F.J.; Sobrado-Sobrado, A.M.; Cidoncha-Moreno, M.Á.; Riesgo-Martín, J.; Pedreira-Robles, G.; Garcimartin, P. Impact of Telenurse-Led Intervention in Clinical Trials on Health Literacy, Empowerment, and Health Outcomes in Patients with Solid Tumours: A Pilot Quasi-Experimental Study. *BMC Nurs.* **2024**, *23*, 86. https://doi.org/10.1186/s12912-023-01641-x.
105. Ali Ibrahim, R.; Fathy Mohamed, R.; Mohamed Allam, H. Effect of Educational Program on Quality of Life and Health Promoting Lifestyle Behaviors for Patients with Inflammatory Bowel Disease. *J. Nurs. Sci. Benha Univ.* **2024**, *5*, 55–73. https://doi.org/10.21608/jnsbu.2024.362179.
106. Seo, J.; Goodman, M.S.; Politi, M.; Blanchard, M.; Kaphingst, K.A. Effect of Health Literacy on Decision-Making Preferences among Medically Underserved Patients. *Med. Decis. Mak.* **2016**, *36*, 550–556. https://doi.org/10.1177/0272989X16632197.
107. Ahmed, S.; Newton, P.D.; Ojo, O.; Dibley, L. Experiences of Ethnic Minority Patients Who Are Living with a Primary Chronic Bowel Condition: A Systematic Scoping Review with Narrative Synthesis. *BMC Gastroenterol.* **2021**, *21*, 322. https://doi.org/10.1186/s12876-021-01857-8.
108. Saeed, S.A.; Masters, R.M. Disparities in Health Care and the Digital Divide. *Curr. Psychiatry Rep.* **2021**, *23*, 61. https://doi.org/10.1007/s11920-021-01274-4.
109. Heponiemi, T.; Jormanainen, V.; Leemann, L.; Manderbacka, K.; Aalto, A.-M.; Hyppönen, H. Digital Divide in Perceived Benefits of Online Health Care and Social Welfare Services: National Cross-Sectional Survey Study. *J. Med. Internet Res.* **2020**, *22*, e17616. https://doi.org/10.2196/17616.
110. Aguas Peris, M.; Del Hoyo, J.; Bebia, P.; Faubel, R.; Barrios, A.; Bastida, G.; Valdivieso, B.; Nos, P. Telemedicine in Inflammatory Bowel Disease: Opportunities and Approaches. *Inflamm. Bowel Dis.* **2015**, *21*, 392–399. https://doi.org/10.1097/MIB.0000000000000241.
