# Supplementary figures and images for: Health Literacy in Inflammatory Bowel Disease: A Systematic Review of Health Outcomes, Predictors and Barriers
Source: J Clin Med. 2025 Dec 3;14(23):8577. doi: 10.3390/jcm14238577 (PMC12693092; doi:10.3390/jcm14238577)

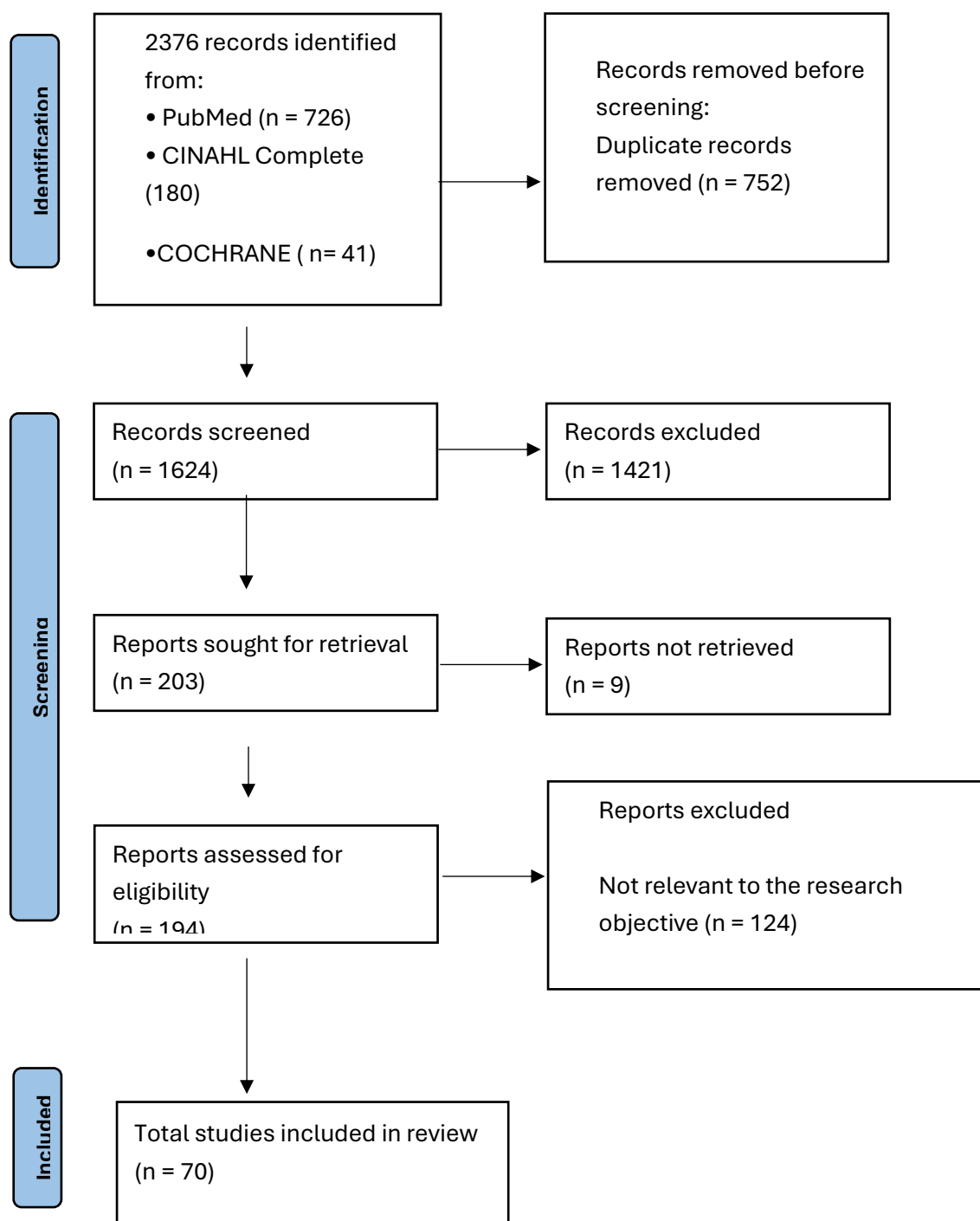

Figure S1. PRISMA 2020 flow diagram model for systematic reviews

Supplement: Supplementary file 1 [file jcm-14-08577-s001.zip › Figure S1. PRISMA 2020 flow diagram model for systematic reviews.pdf]

Figure S2. Distribution of QuADS percentage scores

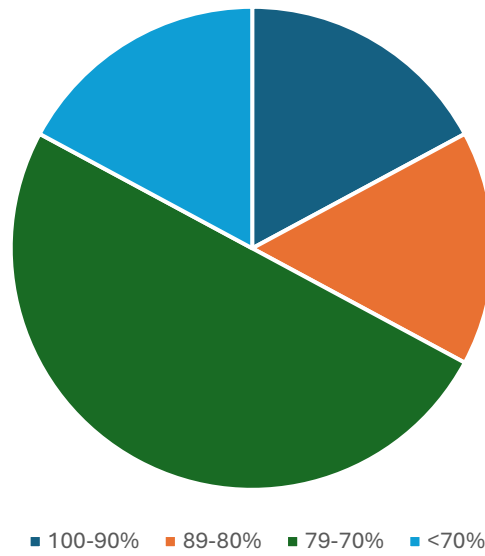

Supplement: Supplementary file 1 [file jcm-14-08577-s001.zip › Figure S2 Distribution of percentage score.pdf]

Figure S3 Predictors and Barriers HL

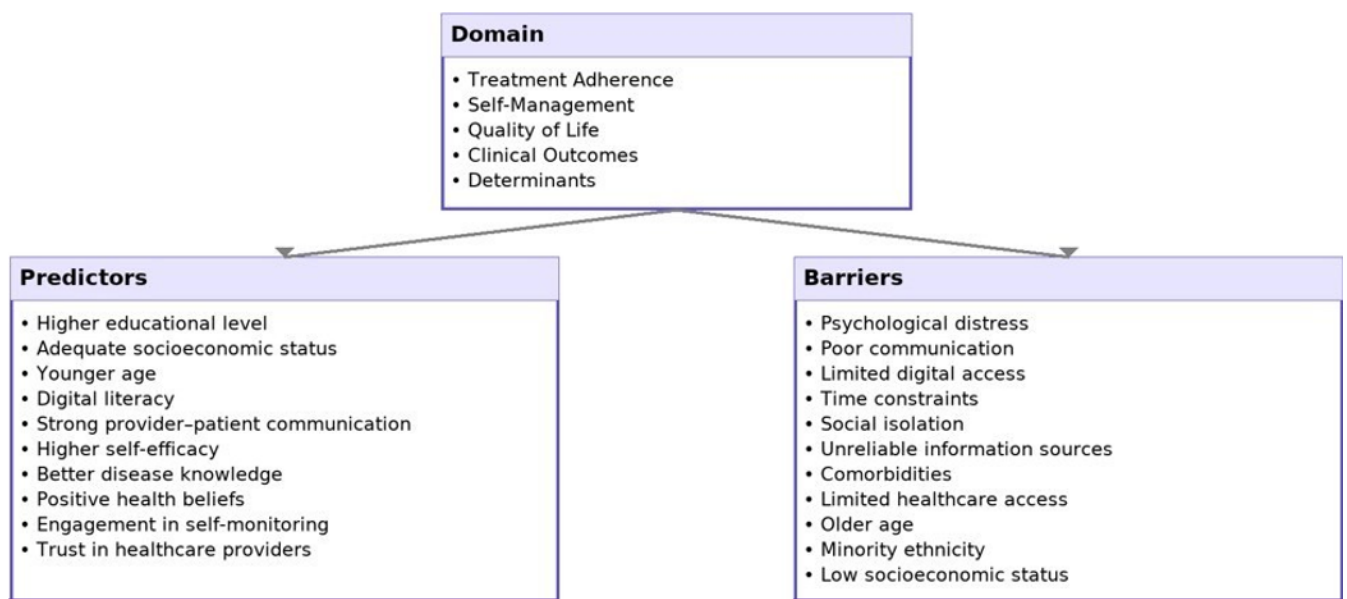

Supplement: Supplementary file 1 [file jcm-14-08577-s001.zip › Figure S3 Predictors and Barriers HL.pdf]
